# Unsupervised EEG preictal interval identification in patients with drug-resistant epilepsy

**DOI:** 10.1038/s41598-022-23902-6

**Published:** 2023-01-16

**Authors:** Adriana Leal, Juliana Curty, Fábio Lopes, Mauro F. Pinto, Ana Oliveira, Francisco Sales, Anna M. Bianchi, Maria G. Ruano, António Dourado, Jorge Henriques, César A. Teixeira

**Affiliations:** 1 Centre for Informatics and Systems of the University of Coimbra, Department of Informatics Engineering, University of Coimbra, Coimbra, Portugal; 2 Epilepsy Centre, Medical Centre, Department of Neurosurgery, University of Freiburg, Freiburg, Germany; 3 Refractory Epilepsy Reference Centre, Centro Hospitalar e Universitário de Coimbra, EPE, Coimbra, Portugal; 4 Department of Electronics, Information and Bioengineering, Politecnico di Milano, Milan, Italy; 5 Department of Electronics and Informatics Engineering, University of Algarve, Faro, Portugal

**Keywords:** Epilepsy, Predictive markers

## Abstract

Typical seizure prediction models aim at discriminating interictal brain activity from pre-seizure electrographic patterns. Given the lack of a preictal clinical definition, a fixed interval is widely used to develop these models. Recent studies reporting preictal interval selection among a range of fixed intervals show inter- and intra-patient preictal interval variability, possibly reflecting the heterogeneity of the seizure generation process. Obtaining accurate labels of the preictal interval can be used to train supervised prediction models and, hence, avoid setting a fixed preictal interval for all seizures within the same patient. Unsupervised learning methods hold great promise for exploring preictal alterations on a seizure-specific scale. Multivariate and univariate linear and nonlinear features were extracted from scalp electroencephalography (EEG) signals collected from 41 patients with drug-resistant epilepsy undergoing presurgical monitoring. Nonlinear dimensionality reduction was performed for each group of features and each of the 226 seizures. We applied different clustering methods in searching for preictal clusters located until 2 h before the seizure onset. We identified preictal patterns in 90% of patients and 51% of the visually inspected seizures. The preictal clusters manifested a seizure-specific profile with varying duration (22.9 ± 21.0 min) and starting time before seizure onset (47.6 ± 27.3 min). Searching for preictal patterns on the EEG trace using unsupervised methods showed that it is possible to identify seizure-specific preictal signatures for some patients and some seizures within the same patient.

## Introduction

Epilepsy research spans different areas, with researchers directing great efforts towards discovering electrophysiological biomarkers that may enable the design of seizure prediction models^1^. The ability to predict when a seizure will occur has been linked to the potential of either stopping that seizure or preventing adverse effects stemming from its occurrence^1^. Specifically, available nonpharmacological treatments aiming at seizure controlling, e.g., the use of intervention devices that enable electrical stimulation or administration of acute medication, rely on the correct prediction of seizures. Improving the efficacy of these treatments can become of great usefulness for people diagnosed with drug-resistant epilepsy (DRE)^1,2^. Patients with DRE represent about one-third of all patients with epilepsy and have their lives limited due to the recurrent spontaneous nature of seizures that cannot be prevented by delivering chronic antiseizure medication^3^.

Seizure prediction models have been developed for over 40 years with the aim of discriminating between periods of normal, seizure-free brain activity (interictal state) and pre-seizure changes (preictal state)^4^. However, despite initial encouraging results, only recently have researchers proved that prospective seizure prediction is possible, at least for some patients^5^. Comprehensive reviews have provided guidelines for performance assessment and statistical validation of seizure prediction studies^4,6^. Adopting these guidelines has demonstrated that seizure prediction models generally perform poorly, being successful for only some patients. The heterogeneity of the ictogenesis mechanisms among seizures (intra- and inter-patient) can contribute to the unsatisfactory performance of current seizure prediction models^7,8^. As such, understanding the transition from interictal to ictal states can greatly influence the prediction performance, demanding for a proper characterisation of the preictal interval.

Literature shows evidence of the preictal interval firstly by observing changes in the electroencephalography (EEG) recordings seconds to hours before the seizure onset and, additionally, by the reported predictability of seizures^4,9^. Starting in the early 1990s, with the application of the mathematical theory of nonlinear dynamics, the preictal interval became associated with the state during which the brain activity evolves deterministically towards the seizure^7,10^. In other words, once the brain enters this state, a “point of no return” has been passed, meaning that the seizure will occur^6,7,11^. In practice, integrating preictal information in supervised seizure prediction models consists of using preictal interval annotations to perform a prediction within a given prediction horizon. If an alarm is raised during this interval, it is counted as a true prediction; otherwise, it is considered a false prediction^4,6,8,11^. As there is no clinical definition of the preictal interval, several studies present seizure prediction models using a fixed interval (typically a value in the range of 2–90 min)^6,12^. Since 2005, concerns regarding preictal’s starting time have been addressed through the evaluation of different preictal interval durations^13–22^. There are statistical approaches that typically compare the distributions of interictal and preictal intervals and there are algorithmic approaches performing a grid-search on the preictal interval^6^. In statistical approaches, a range of different preictal intervals is compared with the corresponding interictal intervals, with a conclusion being drawn for the most discriminating preictal interval. In algorithmic approaches, a range of intervals is considered when training algorithms and the one leading to the best prediction performance is integrated into the final prediction algorithm. In the literature, preictal intervals ranging from 2 to 240 min have been inspected. The highest discrimination (in the case of statistical analysis) or best performances (in the case of a seizure prediction algorithm) correspond to an average preictal interval in the range of 28–60 min^13–19,21,22^. However, in some cases, considering preictal intervals longer than 60 min could have changed this average, given that for some patients, this was the longest analysed interval yielding the best performance^14,16,19^. The statistical approaches assessing preictal intervals with different duration indicate that the selected preictal interval varies among univariate and bivariate features and among seizures experienced by the same patient. Mormann et al.^13^ reported that bivariate features were associated with longer preictal intervals, possibly indicating a higher sensitivity to long-term seizure dynamics. Bandarabadi et al.^18^ reported seizure-specific preictal alterations in 70% of seizures. Despite being an improvement to the use of a fixed preictal interval for an entire group of patients, performing a patient grid-search on a user-defined range of preictal intervals still does not accurately address the variability of the seizure generation process.

Additionally, given that prediction models try to correctly classify interictal and preictals samples, it is natural that the prediction performance heavily relies on feeding the models with accurate preictal information. It follows that using unsupervised learning methods seems to have the potential to provide precise insight into the existence of preictal activity and possibly mitigate the limitation of a user-defined preictal interval. Moreover, it introduces the possibility of dealing with the heterogeneity of the ictogenesis mechanisms within the same patient. A proper characterisation will likely be reflected in obtaining more accurate, seizure-specific annotations of the preictal intervals that can be further integrated into the prediction algorithms during the training phase.

Unsupervised learning methods have been scarcely employed to automatically identify preictal activity. The first study was conducted in 2005 by Le Van Quyen et al.^23^ whom reported the use of K-means clustering algorithm to build a library of interictal patterns based on the analysis of the degree of phase synchronisation. The interictal recordings were found to generally fit into 5 to 10 clusters, suggesting the existence of recurrent patterns of interictal activity. The results varied widely among patients^23^. From 2019 onward, the use of unsupervised learning methods for preictal determination was resumed^24–26^. Results across studies indicate that the preictal interval may manifest in human electrographic data only for some seizures (in 41% of seizures in ECG data^26^ and ranging from 38% to 70%^23–25^ in EEG data). The preictal heterogeneity observed within seizures for the same patient supports exploring seizure-specific preictal profiles^8,27–29^.

Our study explores the existence of preictal intervals in EEG data using unsupervised learning methods. First, we extracted univariate and multivariate features from 4.5 h of EEG data recorded before seizure onset. Second, we applied four clustering methods to each seizure’s feature data obtained after dimensionality reduction. Then, we performed a visual inspection in search of any pattern that could be distinguishable from interictal activity in the 2 h preceding seizure onset. When those patterns were identified, we characterised them in terms of duration, density, and starting time.

## Methods

**Figure 1:**
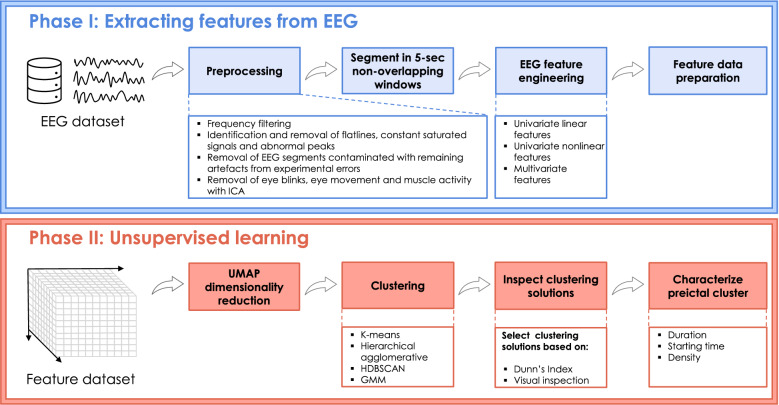
Block diagram of the proposed methodology. The study’s first phase corresponded to preprocessing and feature engineering and preparation. The second phase encompassed dimensionality reduction of each feature group (univariate linear, univariate nonlinear and multivariate), followed by the application of unsupervised learning methods and the preictal interval inspection.

The following sections describe each step performed to explore the preictal interval in EEG data from patients with DRE (see Fig. 1). We started by preprocessing the EEG recordings to minimise the effects of possible confounding artefacts. Then, we extracted handcrafted features from the preprocessed EEG data. Afterwards, given the obtained high-dimensional feature space, we performed dimensionality reduction and applied four clustering methods to the reduced three-dimensional feature space. We visually inspected each seizure’s data distribution and clustering solutions in search of preictal activity. When a cluster has been discovered for a given seizure in the 120 min before onset, we considered it evidence of the preictal state, and we gathered information on its starting time, duration, and density.

### Database

The European Epilepsy Database, also known as the EPILEPSIAE database, provided the dataset used in this study. Data were recorded in patients with DRE under presurgical monitoring at three hospitals: Epilepsiezentrum, Universitätsklinikum Freiburg (Germany), Centro Hospitalar e Universitário de Coimbra (Portugal), and Hôpital de la Pitié-Salpêtrière, Paris (France)^30,31^. During the hospital stay, seizure frequency was increased by reducing the number and dose of antiepileptic medication^30^. The local ethics committees of the three hospitals involved in the database development (Ethik-Kommission der Albert-Ludwigs-Universität Freiburg; Comité consultatif sur le traitement de l’information en matière de recherche dans le domaine de la santé, Hôpital de la Pitié-Salpêtrière; and Comité de Ética do Centro Hospitalar e Universitário de Coimbra) approved the recording and use of data for research purposes. Informed consent was obtained from patients and the parents and/or legal guardians of patients under 18 years of age.

We inspected a group of 41 patients (24 male; age range: 13-67 years; mean age: $41 \pm 16$ years) for whom only seizures occurring in the temporal lobe have been annotated. Patient data contains scalp EEG, and ECG signals recorded simultaneously. Data from 19 EEG electrodes (Fp1, Fp2, F7, F3, Fz, F4, F8, T7, C3, Cz, C4, T8, P7, P3, Pz, P4, P8, O1, and O2), sampled at 256 Hz, were analysed. Refer to Supplementary Section 2 for more details on patient and seizure metadata. This dataset, previously analysed by Leal et al.^26^, has also been analysed here for comparison purposes.

This study concerns the inspection of EEG data acquired for lead seizures, i.e., for seizures preceded by at least 4.5 h of seizure-free interval, therefore considered as independent events^22,32^. Given this criterion, 162 seizures separated by less than 4.5 h were discarded from a total of 388 seizures, leading to the 226 seizures considered herein.

### EEG signal preprocessing

EEG preprocessing is crucial to allow a meaningful application of clustering methods. But it must be performed in a way that appropriately preserves the useful, brain-related information contained in the EEG. One important step is to remove artefacts naturally occurring in the non-controlled environment of presurgical monitoring. This step was performed using the algorithm developed by Lopes et al.^33^ described below. First, the scalp EEG signals were filtered using a 0.5–100 Hz bandpass 4th-order Butterworth filter and a 50 Hz 2nd-order notch filter. Secondly, segments containing flatlines, constant saturated signals and abnormal peaks were automatically identified and discarded. Then, the 4.5-h signals were divided into 10-min segments.

After automatically removing the remaining experimental errors, the EEG segments were re-referenced to average reference and decomposed using extended infomax independent component analysis. Some of the obtained independent components (ICs) would still contain artefacts, including eye blinks, eye movements and muscle activity. As such, a deep neural network (DNN) model was used to classify the ICs as brain-related or artefact. The model was trained on 61092 ICs from 20 patients and tested on 16334 ICs from 5 patients. Two trained experts labelled each IC as brain or noise based on three plots: the IC time series, the IC power spectral densities (PSDs) and the IC topographic maps. Given the low number of EEG channels (19), some ICs would contain both brain and artefact information. To avoid the loss of valuable brain information, these ICs were still labelled brain-related. The experts’ labelling then favoured retaining the maximum of brain information at the cost of also keeping some physiological artefacts such as muscle activity. Sensitivity and specificity of 93% and 94%, respectively, were reported after applying the DNN model to the test dataset.

The EEG signals used in the feature engineering phase then resulted from the signal reconstruction using the brain-related independent components automatically classified with the DNN model.

### EEG feature engineering

Several features were extracted from EEG data. It is common to group features into (i) univariate linear, capturing, for example, the characteristics of the frequency spectrum in different frequency bands, (ii) univariate nonlinear, capturing the nonlinear behaviour of the EEG, and (iii) multivariate measures, measuring brain connectivity patterns (refer to Supplementary Section 3 for more details). The frequency bands considered in univariate linear and multivariate feature extraction comprise delta (0.5 to <4 Hz), theta (4 to <8 Hz), alpha (8 to < 13 Hz), beta (13 to <30 Hz), and gamma (30 to < 47 Hz)^6,12,14,34^.

We computed each value of the features from 5-second non-overlapping windows of EEG^12^. The final feature dataset comprised 42 univariate linear features per channel, 29 univariate nonlinear features per channel and 495 multivariate features.

### Feature data preparation

We removed information contained in the 10 min before the seizure onset and the 30 min after the previous seizure offset, in the case of subsequent seizures (refer to Supplementary Fig. S1 for a detailed explanation). The 30-min interval corresponds to the period following the ictal discharge, known as postictal, that may be captured in the electrographic trace^13,35–39^. The 10-min interval corresponds to the seizure prediction horizon (SPH)^21,22,40–42^. Accordingly, we searched for preictal patterns until a maximum of 4.5 h of EEG data before seizure onset and a minimum of 4 h (in case of subsequent seizures separated by exactly 4.5 h and excluding the postictal interval). After excluding the SPH interval (and the postictal interval when necessary), we effectively analysed a maximum and a minimum of 4.33 and 3.88 h of EEG data (mean±std: 4.31 ± 0.07 h) per seizure.

Inspection of the feature dataset resulted in identifying constant and quasi-constant features in the three feature groups (univariate linear, univariate nonlinear, and multivariate). Constant features correspond to features for which all values are equal. Quasi-constant features correspond to features for which more than half of the values are equal. Constant and quasi-constant features were discarded from the analysis (refer to Supplementary Section 4 for further details).

Afterwards, we applied the z-score normalisation to each feature group dataset.

### Dimensionality reduction

The feature dataset of each seizure contains 741 univariate linear, 532 univariate nonlinear, and between 235 and 329 multivariate features. Dimensionality reduction was applied to obtain the three-dimensional space where clusters are further drawn. This way, it was possible to visually inspect and interpret the clustering results.

Uniform Manifold Approximation and Projection for Dimension Reduction (UMAP), a recently proposed nonlinear manifold dimensionality reduction method, was used in this study. This method produces low-dimensional datasets while preserving the local and global structure of the original data^43^. The basic principle of this graph algorithm is to keep similar points close and dissimilar points apart^44^. Although we have tried other feature reduction methods (principal component analysis and t-distributed Stochastic Neighbour Embedding), we concluded that UMAP more consistently presented separated rounded or elongated clusters. The UMAP’s superiority over the other state-of-the-art methods has also been widely reported in the literature^43–46^. As such, we decided to conduct the unsupervised learning task on the UMAP-reduced data.

UMAP starts by building a high dimensional weighted graph representation of the data where the edge weights correspond to the likelihood that two points are connected. Then a cost function is used to optimise a low-dimensional graph while maintaining the original structural similarity. UMAP has two main input parameters that control the trade-off between local and global structures: the number of nearest neighbours and the minimum distance. The former defines the number of nearest neighbours required to obtain the initial high-dimensional graph. The latter corresponds to the minimum distance between points in low-dimensional space^44^.

**Figure 2:**
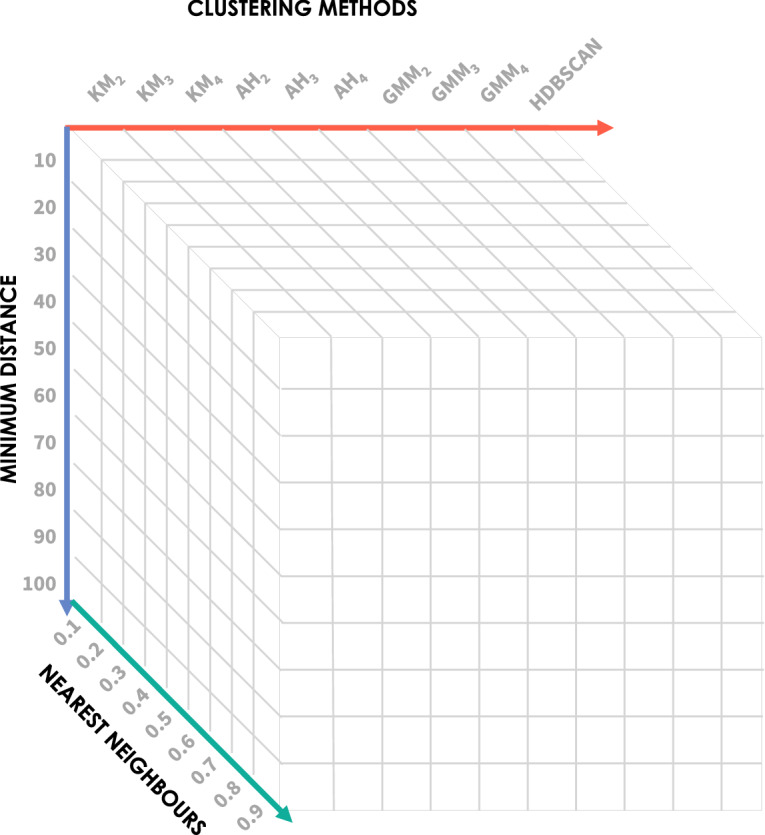
Representation of the number of clustering solutions obtained for each seizure and feature group. Four clustering methods were applied to UMAP-reduced three-dimensional datasets (KM: K-means clustering for $k=2, 3, 4$, AH: agglomerative hierarchical clustering for $k=2, 3, 4$, GMM: Gaussian mixture models for $k=2, 3, 4$ and HDBSCAN). Parameter tuning was performed for UMAP (ten values of nearest neighbours and nine values of minimum distance). The final reduced data obtained before each seizure’s onset and for each feature reduction method corresponds to the maximum DI obtained among the computed clustering solutions.

We performed hyperparameter tuning for UMAP (see Fig. 2). Namely, we applied UMAP considering different values of nearest neighbours (ten values in the range of [10, 100]) and minimum distance (nine values in the range of [0.1, 0.9]). In the next section, we elaborate on finding the best parameters.

### Unsupervised learning (clustering)

Four clustering methods were applied to the three-dimensional datasets obtained for each seizure, resulting from applying UMAP (for each hyperparameter combination): k-means, agglomerative hierarchical, hierarchical density-based spatial clustering of applications with noise (HDBSCAN) and Expectation-maximisation clustering using Gaussian mixture models^26^.

With the previous clustering methods (described in detail in Supplementary Section 5), we aimed to maximise the possibility of identifying preictal signatures across the different data spatial distributions (observed after dimensionality reduction for each seizure). The different clustering methods were chosen according to each method’s ability to identify a specific shape of clusters, whether it be round, elongated, or other arbitrary shapes. Whenever a clustering method was applied, we evaluated the obtained clustering solution using the Dunn’s Index (DI) cluster evaluation metric^47^. Specifically, for each seizure, we selected the best parameter combination for UMAP by searching for the maximum DI value among the values obtained for each clustering method (see Fig. 2).

After parameter tuning, we obtained the final UMAP-reduced data for each seizure, yielding 226 three-dimensional representations for each feature group. For each seizure’s reduced data, the final clustering solution would be given by the clustering method yielding the maximum value of DI. A visual inspection was then performed to determine if the clustering method selected with the DI matched the observed clusters in each seizure’s reduced data. There were a few cases for which no match was achieved. We visually inspected the results obtained in those cases by applying the described clustering methods. If none of these methods could capture the observed clusters, we would increase the number of clusters in agglomerative hierarchical, K-means and Gaussian mixture models in trying to fit visual inspection. When a good fit was not achieved by increasing the number of clusters, we would try DBSCAN for different values of $\varepsilon$, as performed in another study^26^.

### Searching for preictal patterns

Having selected the best parameters for UMAP, we inspected the reduced datasets and the respective clustering solutions in the search for preictal alterations. Published works^14,16,19^ report changes 1 h before seizures, with significant inter- and intra-patient variability. To take that into account, we considered that a preictal interval would start 120 min before seizure onset. We assumed that a putative preictal activity would manifest as abnormal fluctuations in the EEG feature dataset with a higher probability of occurrence starting at the 120 min before the seizure onset until the seizure. In addition, in the case of identifying a preictal pattern in a clustering solution with multiple clusters, that state was typically associated with the cluster that locates, in time, nearer the seizure onset, but not necessarily extending to the onset time (refer to Supplementary Fig. S1, for some examples).

**Figure 3:**
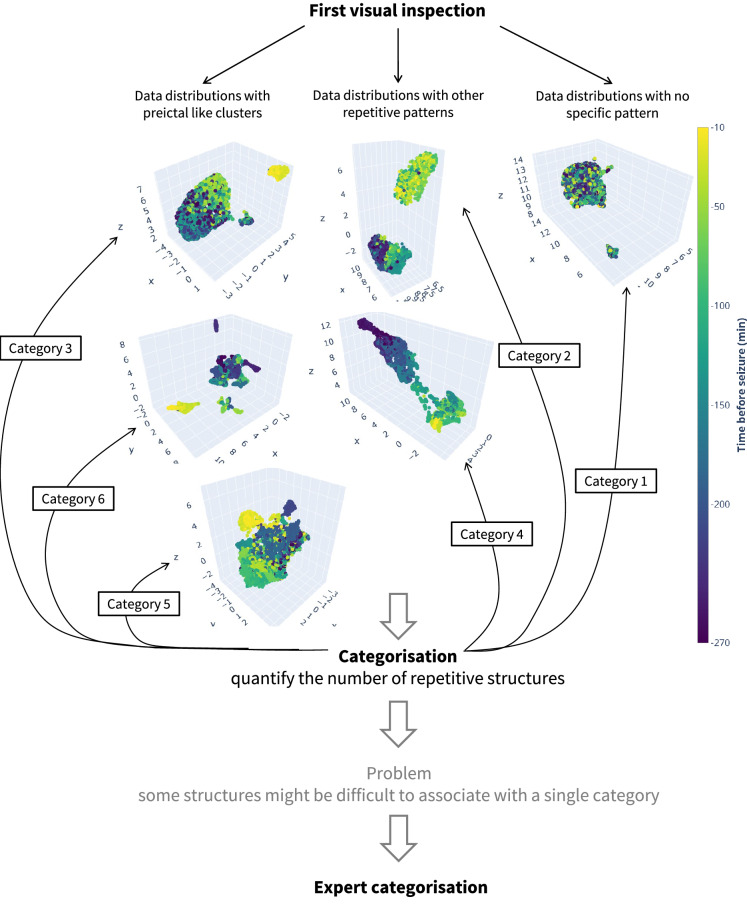
Process of searching for the preictal interval. Each example of the six data distribution categories is represented by the projected UMAP components (labelled x, y, and z). The seizure occurs at 0 min. Category 1 was obtained from patient 21902, seizure 4, reduced multivariate features. Category 2 was obtained from patient 98102, seizure 5, reduced univariate nonlinear features. Category 3 was obtained from patient 58602, seizure 4, reduced multivariate features. Category 4 was obtained from patient 110602, seizure 3, reduced univariate linear features. Category 5 was obtained from patient 98202, seizure 2, reduced univariate nonlinear features. Category 6 was obtained from patient 123902, seizure 2, reduced univariate linear features.

After a first visual inspection of the three-dimensional data distributions, it was possible to identify six data distributions that repeatedly showed across seizures and groups of features. Each category is described in Table 1 and exemplified in Fig. 3. Each seizure’s three-dimensional representation was then assigned to one of the six categories of distributions. This categorisation enabled quantification of the different patterns arising before the seizure onset.

Most data distributions would easily fit one of those six categories. However, there were a few seizures for which the corresponding data distribution could be difficult to associate with a single category. Consequently, we assembled a group of five team members conducting research in the context of seizure prediction to perform this categorisation independently. Each member categorised the clustering solutions into one of the six categories.

After each expert has voted, the final category would correspond to the one gathering three or more votes. If three or more votes were not assigned to a given category, the team would discuss over the reduced data, and, through knowledge exchange, the team would agree on a final category. It is important to note that none of the team members suggested removing an existing category or adding a new category.

**Table 1:** Data distribution categories defined after data reduction and clustering solution inspection.

Category	Definition
Category 1	There is no evidence of a preictal structure. There might be a clear separation into a smaller cluster, but that either comprises samples separated in time or comprises samples strictly located previously to the 120 min before seizure onset
Category 2	Separation into two evenly distributed clusters that might indicate some external interference, such as the transition of the sleep-wake cycle
Category 3	Clear separation into two differently sized clusters, the smaller one resembling a preictal interval located within the 120 min before seizure onset
Category 4	Data distribution indicating progression over time, with samples following a temporal trajectory
Category 5	It seems that a smaller cluster can be identified, but it would be difficult to isolate it in a cluster using clustering methods
Category 6	Category assigned when the clustering solution comprises more than two clusters that may indicate the existence of brain multistates and even progression over time. The preictal interval is represented by the cluster located within 120 min before seizure onset and nearest to the onset. It might be possible to observe evidence of sleep stage transition, preictal interval aside

**Figure 4:**
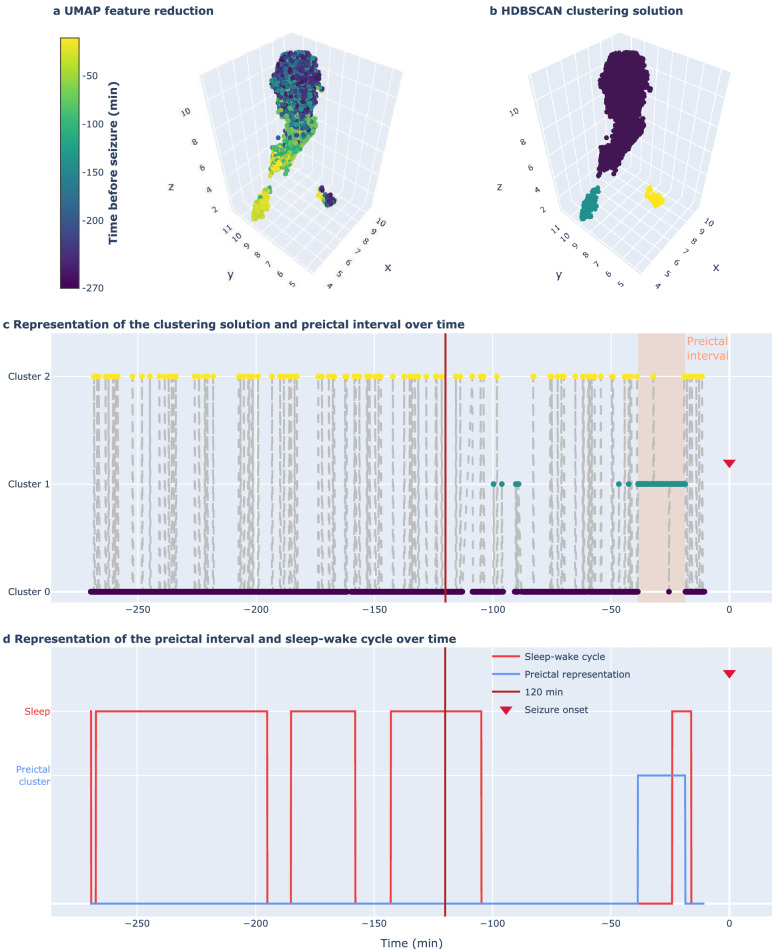
Example of clustering solution inspection. Data was acquired for patient 112802 before the onset of seizure 6. (**a**) UMAP dimensionality reduction was performed on multivariate features. (**b**) The clustering solution was obtained using HDBSCAN. (**c**) Representation of the clustering solution and the preictal interval categorised as category 3. The preictal interval started 38.6 min before seizure onset, lasted for 19.9 min and verified 98.7% density. (**d**) Representation of the preictal interval and sleep-wake cycle, with a *phi* coefficient of 0.18.

Evidence of preictal interval was more reliably observed in the data distribution from categories 3 and 6. After discarding noisy samples, we registered the preictal interval starting and ending samples (see Fig. 4). With this information, we computed three preictal characteristics: interval starting time before seizure onset, interval duration, and density. The preictal density is merely an indicator of the number of preictal samples within the preictal interval defined by the starting and ending times. It corresponds to the number of preictal samples divided by the total number of samples comprised in that interval.

### Sleep-wake cycle detection

After performing a visual inspection of the reduced datasets and corresponding clustering solutions, particularly category 2 solutions, we observed that, in some cases, the clusters’ separation would occur during day-to-night transitions and vice-versa. We are aware that the oscillations observed in the EEG features during the unsupervised study may reflect the copresence of other confounders rather than preictal activity, such as the sleep-wake cycle and/or other internal body circadian cycles. These normal oscillations translate to changes in EEG data distribution over time called concept drifts. We considered that one of the most frequent types of concept drifts present in our data is the sleep-wake cycle. Based on this, we used a model to detect the sleep-wake cycle for each patient and confirm the effect of the sleep-wake cycle on the analysis^48^.

Then, we computed the *phi* coefficient (also known as Matthews correlation coefficient)^49,50^ between the binary sleep-wake vector and a binary vector representing a given cluster distribution for categories 2, 3, and 6. As such, for the case of categories 3 and 6, the binary vector contains ones corresponding to the cluster samples indicating preictal alterations and zeros corresponding to the remaining samples (see Fig. 4). For category 2, as the clustering solution always comprised two equivalently sized clusters, the binary vector would contain zeros and ones corresponding to the samples in each cluster.

Figure 4, depicting reduced data, the clustering solution, and the sleep-wake cycle before onset, was obtained for each seizure’s data and each feature group, for all categories. All figures and developed code are publicly available on GitHub via adrianaleal/eeg-preictal-identification-epilepsy.git. To ensure reproducible results, we set a random seed state on the following Python functions: UMAP, k-means and Gaussian mixture models.

### Comparison with control intervals

The methodology described in the previous sections was repeated for control intervals. These intervals, of 4.5 h duration, ended at the corresponding seizure EEG onset hour but on the day before the seizure. This way, we could compare the results for the 4.5-h interval before the seizure onset with the results for the seizure-free intervals occurring at the same time of the 24-h day. If similar data distributions occur in both intervals, it means that the clusters observed in the 2 h before the seizure onset are not related to preictal activity but instead to another unknown variable.

By selecting the control intervals from the exact same time of the 24-h day, on the previous day, we are controlling for the effect of circadian rhythms on the data distribution. Additionally, given that we are considering a seizure-specific approach throughout the study, the control intervals are located within the interictal time before the onset of the seizure under analysis (see Supplementary Fig. S8 for some examples). Accordingly, a minimum of 33 h of seizure-free signal is required before the onset. In other words, the control intervals start and end at 28.5 and 24 h before the seizure onset, respectively, and are separated by at least 4.5 h from the previous seizure. According to these criteria, we analysed 47 control intervals. These control intervals were only analysed for the univariate linear features, which require less computational time to extract.

### Metadata analysis

We quantified the association between each of the four seizure variables (vigilance state, seizure type, EEG onset hour, and percentage of noise) and the preictal characteristics (starting time, duration, and density) of the seizures for which preictal was found. Notice that we included the percentage of noise determined for each seizure’s 4.5 h of data in order to discard the effect of obtaining a clear cluster separation due to missing feature values introduced by preprocessing. This metadata analysis was performed for each group of features.

## Results

By looking at the examples in Fig. 3, it is possible to conclude that finding evidence of the preictal interval would correspond to obtaining reduced data and clustering solutions categorised as either category 3 or 6. In the case of category 3, we can see a smaller cluster clearly separated from the remaining samples. For the case of category 6, we have data distributions comprising small clusters clearly separated from each other, possibly indicating the existence of different brain states. We assume that some of these states are associated with pre-seizure alterations. To quantify the possible presence of preictal activity, we considered that the preictal would correspond to the cluster located near the seizure. Additionally, category 5 data distributions also inform about clusters of data containing samples that are close in time. However, contrarily to categories 3 and 6, category 5 clusters could not be automatically distinguished and isolated by the clustering methods.

The results for the categorisation performed by each of the five experts are presented in Supplementary Section 6.1. A consensus was not achieved for 8%, 7%, 8%, and 11% of the seizures for the univariate linear, univariate nonlinear, and multivariate, and control univariate linear feature groups, respectively.

**Figure 5:**
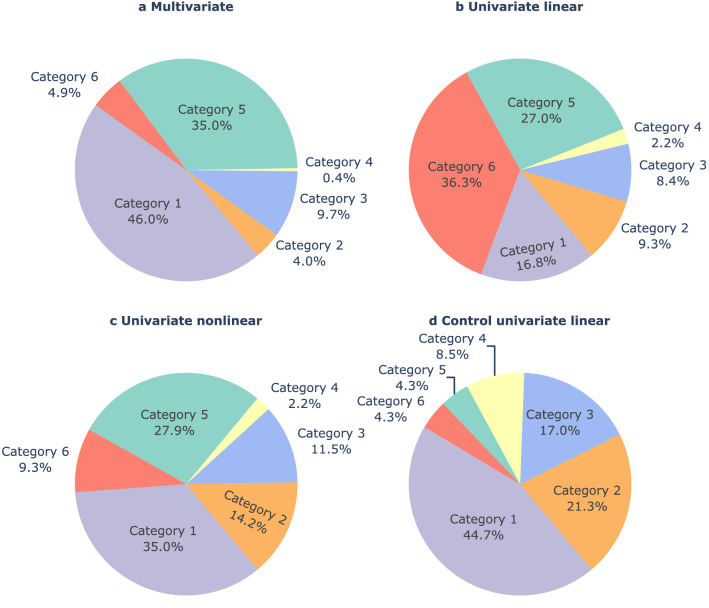
Results for data distribution categorisation. The categorisation of data distributions after experts voting and discussion of doubtful data distributions is presented for each feature group: (**a**) multivariate, (**b**) univariate linear, (**c**) univariate nonlinear, and (**d**) control univariate linear.

Figure 5 presents the prevalence of each category in the three groups of features after analysing the doubtful seizures. Evidence of the preictal interval represented by category 3 was found for all feature groups extracted from the 4.5 h preceding the seizure onset, with similar prevalence (8.4% in univariate linear, 11.5% in univariate nonlinear, and 9.7% in multivariate).

Additionally, data distributions showing several small and structured clusters over time (represented by category 6) were widely seen for the group of univariate linear features. Univariate nonlinear features were the largest source of category 2 data distributions. For this group of features, the clustering methods often could separate two major, evenly sized clusters. This data distribution might indicate a clear transition between two brain states that may not be related to epileptogenic activity but other phenomena, e.g. the sleep-wake cycle.

**Figure 6:**
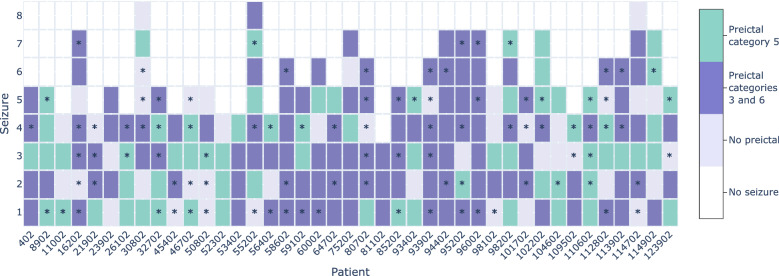
Results for preictal interval identification. The preictal interval was found for 37 patients (90%) and 116 seizures (51%). These results correspond to the evidence of preictal interval found for categories 3 and 6 (together) and category 5, for all groups of features. The results for category 5 were presented for a given seizure when categories 3 or 6 have not been previously assigned in any of the group of features. Asterisks indicate seizures for which preictal patterns have also been identified in a study using ECG data concurrent with the EEG data under analysis (considering that these preictal intervals started before the SPH)^26^. Preictal patterns were found in both EEG and ECG in 22% of the seizures analysed in this study.

Regarding category 5, we observed that this type of data distribution occurred in at least one-quarter of the seizures in all groups of features. At last, we observed a residual prevalence of category 4 distributions. These distributions are characterised by a gradual and continuous evolution of the samples’ trajectory over the analysed data. Analysis of the distributions obtained for the reduced univariate linear features group resulted in categorising the lowest number of uninformative distributions. Namely, only 16.8% of seizures in the univariate linear group belonged to category 1.

Figure 6 shows information about the existence of preictal activity for each of the analysed seizures and patients. Among the 41 patients selected for this study, there were 37 for whom at least one seizure showed a distinct pattern in the reduced data (either with univariate linear, univariate nonlinear or multivariate) that might indicate a preictal alteration (categories 3 or 6). From the 226 seizures studied, 116 seizures (51%) were categorised as containing distinctive pre-seizure information. Multivariate feature reduction led to the identification of preictal clusters in four seizures for which preictal clusters were not found with univariate features. Category 5 preictal appearance is characterised by data clustered in time, however, with a less distinguishable separation from the remaining samples. If we include category 5 in preictal quantification, preictal patterns increase to a total of 183 seizures (81%) in 41 patients.

We compared this article’s information with the results reported in a previous study regarding the search for preictal patterns in ECG data (acquired simultaneously with EEG in the group of patients selected for the current study)^26^. In that work, preictal clusters were found for 41% of the seizures and 90% of the patients. As shown in Fig. 6, the preictal interval was identified both in EEG and ECG (considering categories 3 and 6) in 50 out of 226 seizures (22%). Additionally, when comparing the starting time before the seizure onset between both modalities (see Supplementary Section 6.5), it was possible to observe that while the preictal intervals started mainly 20–40 min before the onset in the EEG recordings, in the ECG there was also a large number of preictals starting from 70 to 120 min.

**Figure 7:**
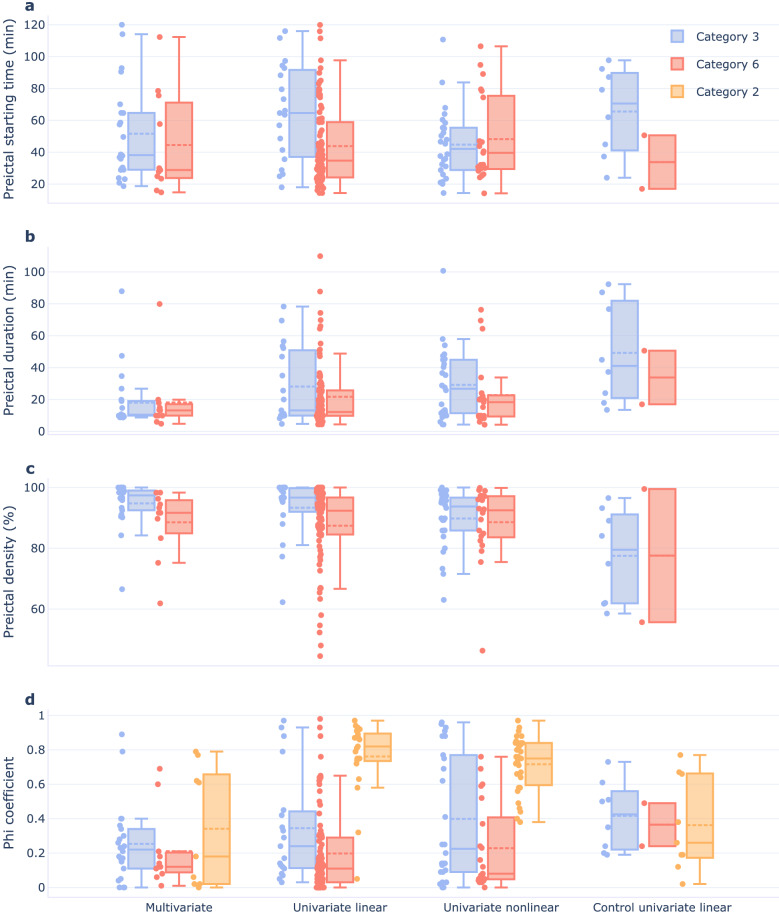
Results for preictal interval characterisation. Preictal interval was characterised for categories 3 and 6, for the three groups of features, according to three characteristics: (**a**) starting time before seizure onset, (**b**) duration, and (**c**) density. (**d**) Phi coefficient was computed between preictal clustering solution and the sleep-wake cycle for categories 3, 6, and 2 for the three groups of features. Dots correspond to one of the three preictal characterising variables or to the phi coefficient. Solid and dashed lines indicate medians and means, respectively. Box’s tops and bottoms correspond to the 75th and 25th percentiles, respectively. Whiskers refer to the span of the preictals characteristics or the phi coefficient after discarding outliers.

Figure 7 presents the statistics of the preictal interval characteristics when it was found and assigned categories 3 or 6. The distributions of preictal (i) starting time before seizure onset, (ii) duration, and (iii) density are depicted. The average preictal’s starting time, computed over all seizures and feature groups, was $47.6\pm 27.3$ min (mean ± standard deviation). It started in the 40 min preceding seizure in 53.0% of the preictal clusters. It lasted for $22.9\pm 21.0$ min (mean ± standard deviation) and was often nearly continuous (90% density observed for 62.4% of preictal clusters found for all groups of features). Contrarily to the other features groups, results show that the vast majority (84.8%) of preictals found for the multivariate group lasted less than 20 min. Additionally, we observed preictal alterations ending at the seizure onset in 45.3% of the preictal clusters identified for categories 3 and 6.

For some seizures, a preictal cluster was found for more than one feature group. To perform a comparison with state-of-the-art studies, it was necessary to select a final preictal interval to compare with. This selection was performed according to the preictal intervals characteristics (refer to Supplementary Section 2). The average of the final preictal intervals’ starting time before seizure onset found in our study ($50.1\pm 28.9$ min) falls in the range of average preictal intervals (28–60 min) obtained by performing grid-search to develop seizure prediction algorithms^13–19,21,22^. Additionally, we compared the starting time of the preictal intervals identified using unsupervised learning with the preictal intervals found using grid-search supervised learning on EEG data from the EPILEPSIAE database (refer to Table S9 and Fig. S18 in Supplementary Section 6.6). Namely, there are two studies^21,22^ documenting results of preictal grid-search, which also report the identification number for each patient. Providing that information allows for a more straightforward comparison, though not ideal, as we obtained seizure-specific preictal intervals only for some seizures (within the same patient) rather than all seizures. The vast majority of patient averaged preictal intervals found in these two studies started between 65 and 40 min before seizure onset. Conversely, using the unsupervised learning approach led to the identification of averaged preictal patterns starting at very distinct times before seizure onset, mainly occurring between 80 and 20 min before seizure onset.

Figure 7d presents the values of the phi coefficient between the obtained putative preictal binary representation and the sleep-wake cycle for categories 3 and 6. We observed more than 80% association between both vectors in 5.9%, 12.8%, and 3.0% of the preictals in univariate linear, univariate nonlinear, and multivariate feature groups, respectively. We also computed the association between category 2 two-cluster solutions and the sleep-wake cycle. Association above 80% was found for 57.1%, 37.5%, and 0.0% of univariate linear, univariate nonlinear, and multivariate data distributions, respectively.

Comparing the results for control intervals and intervals preceding seizures for the univariate linear feature group, it was possible to conclude that the prevalence of category 6 data distributions drastically reduced in the control intervals (decreasing from 36.3% to 4.3%). However, a high prevalence of category 3 data distributions was also observed (increasing from 8.4% to 17.0%). For the eight seizures assigned category 3 in the control intervals, Fig. 7 shows that the putative preictal starting time and duration are more spread compared to the other three feature groups. Additionally, we visually inspected the data distributions of the 4.5 h of data preceding seizure onset and the corresponding control interval (available on the GitHub page) when the same category had been assigned. We observed similar data distributions in three out of five seizures with the same categorisation.

Regarding the metadata analysis (refer to Supplementary Section 7 for more details), the results showed no evident association when inspecting the relationship between four seizure variables (vigilance state at onset, type of seizure, EEG onset hour, and percentage of noise) and the preictal characteristics (starting time, duration, and density).

## Discussion

We aimed to explore the existence of pre-seizure alterations in EEG data collected from patients with drug-resistant epilepsy under presurgical monitoring. Unsupervised learning methods were applied to provide new insights into the complexity of the transition from interictal activity to seizure. The success of seizure prediction models heavily relies on the accurate characterisation of the preictal interval when it manifests in the biosignal under analysis^4^.

We observed clusters suggestive of preictal activity in 51% of the analysed seizures. This percentage increases to 81% if, in addition to categories 3 and 6, we also include category 5 as an indication of possible preictal activity. These findings are in accordance with previous studies that, using statistical and clustering approaches, reported preictal interval identification on EEG data in 38%^25^, 69%^24^ and 70%^18,23^ of seizures, respectively.

Despite the results obtained for category 3 in the control intervals (an increase from 8.4% to 17.0%), the considerable reduction in category 6 and 5 data distributions (from 63.3% to 8.6%) increases our confidence in the results on the presence of preictal activity in the 4.5 hours preceding seizures observed in the three feature groups.

### Preictal changes in light of the nonlinear nature of brain dynamics

The preictal clusters identified in categories 3 and 6 were clearly separated from the remaining samples. For the case of category 6 data distributions, preictal clusters were also often preceded by other similarly sized clusters. We speculate that the presence of these small clusters might reflect the occurrence of distinct separated states of brain activity. This observation might be aligned with early beliefs that “neuronal networks may have bi(multi)-stable states”^51^, depending on which of the different paths of brain activity lead to the seizure state^51–53^. Studies on the basic mechanisms underlying neural network evolution towards a seizure^51,52^, refer to three possible paths that can lead to abnormal ictal dynamics: (i) a continuous sequence of states reflecting a gradual transition from an interictal to an ictal attractor, (ii) an abrupt change caused by a fast trajectory convergence to ictal state, assuming a system having interictal and ictal attractors simultaneously or (iii) a combination of both^8,54^. In the second case, the transition might result from an abrupt random perturbation, making prediction even more difficult. External or endogenous factors can influence the three types of transition to the ictal state. The fast nonlinear dynamic evolution towards a seizure has been described as the crossing of a threshold, or separatrix, between interictal and ictal states^51–54^. The three scenarios above might explain the results regarding the determination of a preictal cluster. For 49% of seizures, the occurrence of a very fast, sharp transition in brain activity may be missed by the EEG^54^ or by the EEG features (e.g., due to the size of the window under analysis^27^), and, therefore, there are no seizure precursors. For the remaining 51%, we found distinguishable clusters that might reflect either a still sharp, but not so fast, transition or a gradual (possibly multistate) preictal transition^51,55^.

Nearly 20 years after Lopes da Silva et al. study^51^, it is especially interesting to note that we still ask the same question: “which of all these measurable dynamical changes in the state of neuronal networks do lead to an epileptic seizure?”. Particularly for the case of category 6 data distributions, we struggled to provide preictal insight, wondering which clusters (within the 120-min interval) would be indicative of ictogenesis or “normal” brain function. We assumed that preictal activity would correspond to the cluster showing closer to the seizure as this assumption more closely relates to the preictal concept^54^. However, it might be possible that both the preictal and interictal intervals could comprise distinct sub-intervals that we cannot classify into normal or abnormal brain activity but rather on multi-classes representative of such sub-intervals in both main classes.

Additionally, we acknowledge the difficulty in interpreting category 3 reduced data distributions. Namely, two scenarios often occurred when observing clusters within the 120 min before onset in category 3: (i) a gradual interictal to ictal transition reflected in a preictal interval ending on the seizure onset and (ii) a fast (but EEG perceptible) preictal interval not ending at the seizure onset (as in Fig. 4). The first case corresponds to an increase in the features’ value until the seizure onset. Supervised learning methods are typically successful when this scenario occurs as it allows for a binary classification of the data into interictal state and subsequent preictal state. In the second scenario, we hypothesised that, even though regulatory mechanisms may have been triggered in the brain towards seizure suppression (hence the decrease in features value before the seizure onset), the seizure threshold may have been crossed, triggering a seizure^8,55^. Interestingly, some studies on seizure risk forecasting analysing long-term EEG data show a similar behaviour preceding seizures. Karoly et al.^27^ noticed the existence of a peak in seizure likelihood that is followed by a gradual decrease until the seizure onset. This evidence is also depicted in a comprehensive survey^56^, where authors present real-time EEG recorded over five days, (from the previously mentioned study^27^), weighted by the prior risk of seizures given the time of day. The corresponding proictal states and seizure timing are also depicted. Some seizures seem to occur shortly after or during a decrease in the circadian-weighted EEG, within the respective proictal state. Regarding the transition between interictal and preictal intervals, it was often possible to observe “jumps” from the main cluster, e.g., representing the interictal state, to another smaller cluster, a putative preictal state. Importantly, these “jumps” unlikely correspond to a trajectory of samples from interictal to preictal and back to interictal again, but rather a trajectory from a main, non-preictal cluster (that may contain mainly interictal samples spread over the three-dimensional feature space) to a preictal state and back to another location in the main cluster.

In our study, each cluster showing in categories 3 or 6 data distribution seems to reflect the existence of a preictal pattern. Unsupervised learning methods may leverage knowledge on the evolution of the EEG time series during the seizure generation process. Simultaneously, the clusters obtained with unsupervised learning methods can further shed light on the rate of false positives hampering the performance of supervised learning prediction models. However, new questions arise: which brain processes explain these clusters? Are those the reflection of normal brain functioning or pathological phenomena? These questions closely relate to the knowledge gap regarding the influence of, for instance, interictal brain processes (e.g., interictal epileptiform activity) during ictogenesis^7,53,54^. Answering such questions requires performing more studies to provide an insightful interpretation of data distributions and subsequent clustering results. Clinicians’ insight would also be crucial to obtain a ground truth to validate the origin of the different clusters^57^.

### Influence of methodological aspects

The results obtained with nonlinear feature reduction methods such as UMAP may be more suitable to reveal the nonlinear dynamical functioning of the brain. Accordingly, most studies propose nonlinear systems for epilepsy EEG modelling^10,12^. Nevertheless, we are aware that using this nonlinear method and the consequent parameter tuning could significantly impact further data interpretations^58^. Additionally, applying UMAP is limiting as it does not allow extracting information on feature importance.

Regarding the different groups of features extracted, we concluded that univariate features were the major source of data distributions containing preictal clusters (and data distribution heterogeneity). The analysis of the reduced multivariate features led to the observation of preictal clusters for only an additional four seizures compared to the univariate features. Specifically, univariate feature extraction has provided preictal information for 96.5% of the seizures.

The multivariate features are single global measures of functional brain connectivity obtained by applying graph measures to bivariate features. As such, these features reflect global changes in brain activity over time. Even though multivariate and bivariate measures have been associated with high prediction performances, some authors reported preictal alterations predominantly showing in specific channels^6,13,23^. In addition, when we identified preictal clusters in both reduced multivariate and univariate data, the vast majority of these intervals would start at the same point in time and have the same duration for most seizures (see Fig. S9 and S10 in Supplementary Section 6.2).

Using unsupervised learning seems to appropriately address the problem of preictal interval identification and characterisation. Given the missing knowledge regarding the sequence of brain activity leading to a seizure, it might be limiting to define fixed intervals of preictal activity for supervised seizure prediction. In fact, the preictal intervals determined in this study started in the range of 14.2–120 min before seizure onset, which is a range difficult to cover with grid-search supervised learning due to the computational load. This range of preictals’ starting time demonstrates how the constraints of a preictal grid-search over a user-defined range of preictal intervals can influence results. Unsupervised approaches allow for a relaxation of these constraints and, therefore, to increase the probability of finding the correct labels of the preictal interval for each seizure. Additionally, applying clustering methods to physiological data collected before a seizure might unravel seizure-specific preictal profiles that do not arise when conducting the standard interictal versus preictal binary classification.

Nevertheless, unsupervised learning methods are not without limitations. A potential pitfall of the unsupervised methodology corresponds to the difficulty in inferring the source of the different observed clusters. We assume that the cluster located near the seizure onset corresponds to preictal activity that causally led to that seizure. However, these pre-seizure oscillations may not correspond to epilepsy manifestations but be produced by other unrelated confounders (discussed in the next section). Even though we tried to address this concern by assessing the association between preictal manifestations and the sleep-wake cycle, we enforce the need for clinical annotations, either obtained by video monitoring or EEG interictal close observation, in future unsupervised learning studies. Such information may be crucial to strengthen the conclusions derived from preictal interval exploration through unsupervised learning.

At last, we also highlight that even though we have attempted to produce a fully automatic framework for preictal interval exploration, such a goal was not fulfilled. In fact, we have explored clustering evaluation indexes in search of a measure that would automatically identify preictal activity. However, due to the high variability observed among the seizures’ three-dimensional representation, we could not select a measure matching expert visual inspection. Such variability also explains the difficulty in categorising some seizures’ data distributions into only one of the six reported categories. The contribution of our research team was the solution found to overcome this problem. Five experts, all working in the epilepsy field, categorised the data distributions. The problematic cases were discussed, and a final categorisation was achieved. Notwithstanding, resorting to expert voting is a major limitation of this study. Increasing the number of experts would allow for more trustworthy voting and less subjectivity in the results. Thus, we enforce the need for new strategies to allow for an automatic and user-independent unsupervised preictal interval search.

### Influence of confounders in seizure susceptibility

Seizure susceptibility can vary depending on the current brain state, sleep-wake cycle, circadian and ultradian rhythms, medication tapering, stress, or other exogenous and endogenous factors^4,8,13,59,60^. The EEG features may also be subjected to a different interpretation depending on the patient’s age^34^ and aetiology^61^. These factors may help explain the variability observed among seizures and patients that support the development of patient-specific approaches^4,8,11^. Additionally, the existence of a large number of epilepsy syndromes (resulting in considerable heterogeneity concerning aetiology and clinical manifestations) and non-cerebral confounders may also contribute to such variability^7,62^. Accordingly, the results in our study may also be heavily influenced by epilepsy-characterising aspects such as aetiology, age, and lateralisation. However, given our study’s seizure-specific nature, we only performed a metadata analysis using information annotated for each seizure. The results indicate that, in the analysed dataset, there is no significant influence of the vigilance state at seizure onset, type of seizure and EEG onset hour on the obtained results. Nevertheless, future studies should continue the search for correlations between preictal characteristics and available metadata. Such analysis can translate into training models for the different concept drifts present in data (e.g., training seizure prediction models for a given epilepsy aetiology, type of medication or type of seizures)^3,8^.

Applying the unsupervised learning methods to control intervals was performed to address the existence of other variables that might be confused with preictal activity. The fact that we could not obtain control intervals for all seizures is a limitation arising from the analysis of data collected during presurgical evaluation characterised by an increase in seizure frequency and a consequent reduction in seizure-free time. Ideally, this analysis would be conducted on the entire seizure-free data. However, as this is a user-dependent analysis that requires a visual inspection, it would be a time-consuming task. Nevertheless, results show that, in contrast to the 4.5 h of data preceding seizures, there is a low prevalence of data distributions with multiple clusters in control intervals. This difference contributes to increased confidence in the reported results regarding the existence of preictal patterns.

Additionally, the scalp EEG data analysed in this article were collected while patients with DRE were in an epilepsy monitoring unit under presurgical evaluation. Consequently, we explored data that are not representative of normal ambulatory brain activity but rather data collected while the patient was hospitalised for several days^30^. During the hospital stay, patients were submitted to antiepileptic drug tapering to precipitate seizures. Thus, interpreting the results might not directly translate to DRE interictal and preictal functioning during real-world conditions^7,8^. On the one hand, medication withdrawal preceding surgery has been associated with increased seizure susceptibility^7^. On the other hand, the administration of certain types of medication, such as benzodiazepines, has been reported to increase beta wave activity in EEG recordings^34,63^.

The possibility of recording long-term EEG data (days to years) has opened a new avenue in exploring circadian rhythms’ influence on seizure occurrence^27,60,64,65^. Baud et al.^60^ found that interictal epileptiform activity fluctuations are governed by circadian and multidien rhythms, which in turn determine seizure risk in some subjects. Improved seizure forecasting was reported in Karoly et al.^27^ study after integrating information about the circadian rhythm of seizures in patient-specific models. Identifying subjects for which seizures tend to occur during specific phases of the circadian rhythm could explain some patterns of data distributions we have found in our research.

The sleep-wake cycle seems to be associated with the pattern of seizure occurrence^5^. We observed that the highest number of category 3 and 6 seizures, verifying a high association between the sleep-wake cycle and the preictal cluster, was found for the univariate nonlinear features (12.8%). The highest association found for category 2 was 57.1% using the reduced univariate linear features. Such observation may motivate the use of sleep-wake cycle information when developing seizure prediction models, at least for some seizures.

Another possible confounder is the influence of postictal activity. A postictal interval of 30 min has been considered in this study and removed in the case of subsequent seizures. Nevertheless, despite EEG slowing or suppression occurring on average about 5 min after seizure offset, it has been reported to occur 40–60 min after the offset of some seizures^39^. Again, this aspect is more evident when analysing data collected in presurgical monitoring due to reduced interseizure interval.

Despite the thorough preprocessing performed on the scalp EEG recordings, it is important to highlight that physiological artefacts such as muscle artefacts may still be present in the data. This study’s limitation results from the difficulty in distinguishing the EEG power in the frequencies of interest from the muscle artefact frequencies^66,67^.

Additionally, in a previous study, conducted in the same group of patients, preictal changes in heart rate variability have been reported in 41% of the seizures and 90% of the patients, evidencing the effect of seizures in the autonomous nervous system^26^. That study, as well as ours, attempted to characterise preictal patterns using unsupervised learning. However, on the former, three-dimensional combinations of ECG features were inspected, and clustering solutions comprising only two clusters were explored. In the present study, we performed feature reduction to obtain a three-dimensional dataset for each seizure and feature group. Then, we applied clustering methods to search for (i) four clusters, for the case of K-means, agglomerative hierarchical, and Gaussian mixture models clustering and (ii) an unlimited number of clusters in the case of HDBSCAN. These methodological differences may partly explain that preictal changes were identified in EEG and ECG in only 22% of the seizures in the analysed group of patients. Contrarily to EEG, a large number of putative preictal intervals found in ECG started between 70 and 120 min before onset. This might indicate that the cardiac changes captured in the ECG might not directly reflect epilepsy-related cardiac manifestations but rather result from medication oscillations and sleep stages, that induce differences in the activation of brain mechanisms (and consequent autonomic modulation) over normal to seizure transition^68,69^. Nevertheless, such changes may still contain predictive potential.

### Future work

We have laid the path for the retrospective identification of pre-seizure patterns using unsupervised learning methods. However, we admit that it can be challenging to envision future prospective applications. The available preictal clusters discovered during our unsupervised learning study require further validation. Specifically, it is now critical to integrate EEG and ECG preictal activity information in seizure prediction models and compare the obtained performance with the performance of a model integrating a preictal interval derived from grid-search. A practical application could be to train machine learning models using the preictal starting time information found using clustering methods. For instance, it might be possible to train individual models for specific types of seizures or seizures that follow a given circadian pattern if similar preictal intervals are found for these groups. This approach, however, is dependent on the analysis of a considerable number of seizures to train each model. Even though we have not found a correlation between the preictal starting time and seizure metadata (vigilance state, seizure type, and EEG onset hour), we believe that further unsupervised learning studies might reveal such a correlation when exploring other long-term databases. In our case, the problem grows more complicated when a different preictal interval is identified for each seizure within a patient, with no apparent pattern among seizures. When training the models, it is necessary to think about a strategy to use a final preictal interval label, considering that no preictal pattern has been found for some seizures. We suggest a possible hybrid solution that consists in defining a final preictal interval to use in training and testing as the average of the clustering preictal intervals and the grid-search preictal intervals for the remaining seizures.

Less invasive procedures, such as subscalp EEG, have been recently developed for ultra-long-term brain monitoring^70–72^. The method involves implanting subscalp (or subcutaneous) electrodes, for example, unilaterally behind the ear^70^. Subscalp EEG and scalp EEG similarly capture background activity with closed and open eyes, showing a similar signal-to-noise ratio. Additionally, despite subscalp EEG may still be affected by artefacts such as muscle activity, these recordings present improved signal quality compared to scalp EEG, particularly during body movements that produce interferences due to the movement of wires^70–72^. Concomitantly, scalp EEG devices able to collect data from a few electrodes (placed, e.g. in the temporal lobe) are emerging as alternatives to conventional scalp EEG by providing patients with more comfort and usability^73^. As such, we encourage the design of new studies that use unsupervised learning to explore the capacity of, e.g., scalp EEG temporal channels to capture preictal activity.

Additionally, our study may provide evidence for a future application of unsupervised learning to obtain proictal annotations. Namely, semi-supervised annotation methods could be envisioned to facilitate the annotation of periods of seizure risk while still requiring the clinician’s input to obtain the final stratification of seizure risk.

Ultimately, considering multimodal approaches might be crucial to understand seizure generation. There are clearly several factors influencing brain activity shift from normal functioning to seizure that EEG alone cannot capture. Monitoring non-neurological biomarkers such as heart rate, blood pressure, galvanic skin response, and movement might provide critical information regarding seizure triggering and driving mechanisms^4,8,59^.

## Supplementary Information

Supplementary Information.

## Data Availability

The dataset used and/or analysed during the current study are available from the corresponding author upon reasonable request.

## References

[1] Traynelis SF (2020). Epilepsy benchmarks area III: Improved treatment options for controlling seizures and epilepsy-related conditions without side effects. Epilepsy Curr..

[2] Kotwas I (2016). Self-control of epileptic seizures by nonpharmacological strategies. Epilepsy Behav..

[3] Meisel C, Loddenkemper T (2020). Seizure prediction and intervention. Neuropharmacology.

[4] Kuhlmann L, Lehnertz K, Richardson MP, Schelter B, Zaveri HP (2018). Seizure prediction—Ready for a new era. Nat. Rev. Neurol..

[5] Cook MJ (2013). Prediction of seizure likelihood with a long-term, implanted seizure advisory system in patients with drug-resistant epilepsy: a first-in-man study. Lancet Neurol..

[6] Mormann F, Andrzejak RG, Elger CE, Lehnertz K (2007). Seizure prediction: The long and winding road. Brain.

[7] Blauwblomme, T., Jiruska, P. & Huberfeld, G. Mechanisms of ictogenesis. In *International Review of Neurobiology* 1st edn, Vol. 114, Chap. 7 (eds Jiruska, P. *et al.*) 155–185 (Academic Press, 2014). 10.1016/B978-0-12-418693-4.00007-8. 10.1016/B978-0-12-418693-4.00007-8 25078502

[8] Freestone DR, Karoly PJ, Cook MJ (2017). A forward-looking review of seizure prediction. Curr. Opin. Neurol..

[9] Litt B (2001). Epileptic seizures may begin hours in advance of clinical onset: A report of five patients. Neuron.

[10] Iasemidis LD (2003). Epileptic seizure prediction and control. IEEE Trans. Biomed. Eng..

[11] Freestone DR (2015). Seizure prediction: Science fiction or soon to become reality?. Curr. Neurol. Neurosci. Rep..

[12] Bou Assi E, Nguyen DK, Rihana S, Sawan M (2017). Towards accurate prediction of epileptic seizures: A review. Biomed. Signal Process. Control.

[13] Mormann F (2005). On the predictability of epileptic seizures. Clin. Neurophysiol..

[14] Valderrama M (2012). Identifying an increased risk of epileptic seizures using a multi-feature EEG–ECG classification. Biomed. Signal Process. Control.

[15] Teixeira CA (2014). Epileptic seizure predictors based on computational intelligence techniques: A comparative study with 278 patients. Comput. Methods Programs Biomed..

[16] Alvarado-Rojas C (2015). Slow modulations of high-frequency activity (40–140 Hz) discriminate preictal changes in human focal epilepsy. Sci. Rep..

[17] Bandarabadi M, Teixeira CA, Rasekhi J, Dourado A (2015). Epileptic seizure prediction using relative spectral power features. Clin. Neurophysiol..

[18] Bandarabadi M, Rasekhi J, Teixeira CA, Karami MR, Dourado A (2015). On the proper selection of preictal period for seizure prediction. Epilepsy Behav..

[19] Direito B, Teixeira CA, Sales F, Castelo-Branco M, Dourado A (2017). A realistic seizure prediction study based on multiclass SVM. Int. J. Neural Syst..

[20] Tsiouris KM (2018). A Long Short-Term Memory deep learning network for the prediction of epileptic seizures using EEG signals. Comput. Biol. Med..

[21] Pinto MF (2021). A personalized and evolutionary algorithm for interpretable EEG epilepsy seizure prediction. Sci. Rep..

[22] Pinto M (2022). Interpretable EEG seizure prediction using a multiobjective evolutionary algorithm. Sci. Rep..

[23] Le Van Quyen M (2005). Preictal state identification by synchronization changes in long-term intracranial EEG recordings. Clin. Neurophysiol..

[24] Li F (2019). Transition of brain networks from an interictal to a preictal state preceding a seizure revealed by scalp EEG network analysis. Cogn. Neurodyn..

[25] Quercia, A. *et al.* Preictal onset detection through unsupervised clustering for epileptic seizure prediction. In *2021 IEEE International Conference on Digital Health (ICDH)* 142–147. 10.1109/ICDH52753.2021.00026 (IEEE, 2021).

[26] Leal A (2021). Heart rate variability analysis for the identification of the preictal interval in patients with drug-resistant epilepsy. Sci. Rep..

[27] Karoly PJ (2017). The circadian profile of epilepsy improves seizure forecasting. Brain.

[28] Schroeder GM (2020). Seizure pathways change on circadian and slower timescales in individual patients with focal epilepsy. Proc. Natl. Acad. Sci..

[29] Schroeder GM (2022). Multiple mechanisms shape the relationship between pathway and duration of focal seizures. Brain Commun..

[30] Klatt J (2012). The EPILEPSIAE database: An extensive electroencephalography database of epilepsy patients. Epilepsia.

[31] Ihle M (2012). EPILEPSIAE—A European epilepsy database. Comput. Methods Programs Biomed..

[32] Lopes F (2021). Automatic electroencephalogram artifact removal using deep convolutional neural networks. IEEE Access.

[33] Lopes F (2022). Ensemble deep neural network for automatic classification of EEG independent components. IEEE Trans. Neural Syst. Rehabil. Eng..

[34] Mecarelli, O. Normal awake adult EEG. In *Clinical Electroencephalography*, chap. 9 (ed. Mecarelli, O.) 131–152 (Springer, 2019). 10.1007/978-3-030-04573-9_9

[35] Feldwisch-Drentrup H (2011). Identification of preseizure states in epilepsy: A data-driven approach for multichannel EEG recordings. Front. Comput. Neurosci..

[36] Lehnertz K, Dickten H, Porz S, Helmstaedter C, Elger CE (2016). Predictability of uncontrollable multifocal seizures—Towards new treatment options. Sci. Rep..

[37] So NK, Blume WT (2010). The postictal EEG. Epilepsy Behav..

[38] Payne DE (2018). Postictal suppression and seizure durations: A patient-specific, long-term iEEG analysis. Epilepsia.

[39] Pottkämper JCM, Hofmeijer J, van Waarde JA, van Putten MJAM (2020). The postictal state—What do we know?. Epilepsia.

[40] Winterhalder M (2003). The seizure prediction characteristic: A general framework to assess and compare seizure prediction methods. Epilepsy Behav..

[41] Schelter B (2007). Seizure prediction: The impact of long prediction horizons. Epilepsy Res..

[42] Meisel C, Bailey KA (2019). Identifying signal-dependent information about the preictal state: A comparison across ECoG, EEG and EKG using deep learning. EBioMedicine.

[43] McInnes, L., Healy, J. & Melville, J. UMAP: Uniform Manifold Approximation and Projection for Dimension Reduction (2018).

[44] Wang Y, Huang H, Rudin C, Shaposhnik Y (2021). Understanding how dimension reduction tools work: An empirical approach to deciphering T-SNE, UMAP, TriMap, and PaCMAP for data visualization. J. Mach. Learn. Res..

[45] Yang Y (2021). Dimensionality reduction by UMAP reinforces sample heterogeneity analysis in bulk transcriptomic data. Cell Rep..

[46] Ali M, Borgo R, Jones MW (2021). Concurrent time-series selections using deep learning and dimension reduction. Knowl. Based Syst..

[47] Xiong, H. & Li, Z. Clustering validation measures. In *Data Clustering: Algorithms and Applications* 1st edn, chap. 23 (eds Aggarwal, C. C. & Reddy, C. K.) 572–606 (Chapman & Hall/CRC, 2014).

[48] Oliveira, A. C. R. *Sleep-Awake Cycle Evaluation from Long-Term EEG Data: Assessing the Impact in Epilepsy Seizure Prediction*. Ph.D. thesis, University of Coimbra (2021).

[49] Brusco M, Cradit JD, Steinley D (2021). A comparison of 71 binary similarity coefficients: The effect of base rates. PLoS One.

[50] Chicco D, Jurman G (2020). The advantages of the Matthews correlation coefficient (MCC) over F1 score and accuracy in binary classification evaluation. BMC Genomics.

[51] da Silva FHL (2003). Dynamical diseases of brain systems: Different routes to epileptic seizures. IEEE Trans. Biomed. Eng..

[52] da Silva FL (2003). Epilepsies as dynamical diseases of brain systems: Basic models of the transition between normal and epileptic activity. Epilepsia.

[53] Jirsa VK, Stacey WC, Quilichini PP, Ivanov AI, Bernard C (2014). On the nature of seizure dynamics. Brain.

[54] Baud MO, Proix T, Rao VR, Schindler K (2020). Chance and risk in epilepsy. Curr. Opin. Neurol..

[55] Moraes MFD, de Castro Medeiros D, Mourao FAG, Cancado SAV, Cota VR (2021). Epilepsy as a dynamical system, a most needed paradigm shift in epileptology. Epilepsy Behav..

[56] Karoly PJ (2021). Cycles in epilepsy. Nat. Rev. Neurol..

[57] Müller J (2022). Coherent false seizure prediction in epilepsy, coincidence or providence?. Clin. Neurophysiol..

[58] Cunningham JP, Yu BM (2014). Dimensionality reduction for large-scale neural recordings. Nat. Neurosci..

[59] Baud MO, Rao VR (2018). Gauging seizure risk. Neurology.

[60] Baud MO (2018). Multi-day rhythms modulate seizure risk in epilepsy. Nat. Commun..

[61] Mecarelli, O. Pathological EEG patterns. In *Clinical Electroencephalography*, chap. 13 (ed. Mecarelli, O.) 223–235 (Springer, 2019). 10.1007/978-3-030-04573-9_13.

[62] Beck H, Elger CE (2008). Epilepsy research: A window onto function to and dysfunction of the human brain. Dialogues Clin. Neurosci..

[63] Sanei, S. & Chambers, J. A. EEG waveforms. In *EEG Signal Processing and Machine Learning* 2nd edn (eds Sanei, S. & Chambers, J. A.) 15–46 (Wiley, 2021). 10.1002/9781119386957.ch2

[64] Khan S (2018). Circadian rhythm and epilepsy. Lancet Neurol..

[65] Karoly PJ (2020). Forecasting cycles of seizure likelihood. Epilepsia.

[66] Nunez, P. L. & Srinivasan, R. Fallacies in EEG. In *Electric Fields of the Brain* 2nd edn, chap. 2 (eds Nunez, P. L. & Srinivasan, R.) 56–98 (Oxford University Press, 2006). 10.1093/acprof:oso/9780195050387.003.0002.

[67] Wennberg, R. Introduction to EEG for Nonepileptologists Working in Seizure Prediction and Dynamics. In *Epilepsy: The Intersection of Neurosciences, Biology, Mathematics, Engineering, and Physics* 1st edn, chap. 2 (eds Osorio, I., Zaveri, H. P., Frei, M. G. & Arthurs, S.) 23–39 (CRC Press, 2011).

[68] Jansen K, Lagae L (2010). Cardiac changes in epilepsy. Seizure.

[69] Delamont RS, Walker MC (2011). Pre-ictal autonomic changes. Epilepsy Res..

[70] Duun-Henriksen J (2020). A new era in electroencephalographic monitoring? Subscalp devices for ultra-long-term recordings. Epilepsia.

[71] Stirling RE (2021). Seizure forecasting using a novel sub-scalp ultra-long term EEG monitoring system. Front. Neurol..

[72] Hubbard I, Beniczky S, Ryvlin P (2021). The challenging path to developing a mobile health device for epilepsy: The current landscape and where we go from here. Front. Neurol..

[73] Biondi A (2022). Noninvasive mobile EEG as a tool for seizure monitoring and management: A systematic review. Epilepsia.

