# Supplementary material for: Unsupervised EEG preictal interval identification in patients with drug-resistant epilepsy
Source: Sci Rep. 2023 Jan 16;13:784. doi: 10.1038/s41598-022-23902-6 (PMC9842648; doi:10.1038/s41598-022-23902-6)
Supplement: Supplementary file 1 — Supplementary Information. [file 41598_2022_23902_MOESM1_ESM.pdf]

Supplementary material for:

Unsupervised EEG Preictal Interval Identification in Patients with Drug-resistant Epilepsy

Adriana Leal, Juliana Curty, Fábio Lopes, Mauro F. Pinto, Ana Oliveira, Francisco Sales, Anna M. Bianchi, Maria G. Ruano, António Dourado, Jorge Henriques, and César A. Teixeira

1 Study assumptions on the analysed interval of data

Fig. S1 depicts examples of analysed data before a given seizure onset. We provide examples of the analysed intervals of data when seizures are separated by more than 4.5 hours and when seizures are exactly 4.5 hours apart. The seizure prediction horizon (SPH) and the postictal interval are also indicated.

To minimise the influence of the postictal state on the EEG trace, we considered a postictal interval of 30 minutes. As such, when two seizures were separated by exactly 4.5 to 5 hours, we discarded data corresponding to a postictal interval of 30 minutes to 1 minute, respectively[1–3].

The duration of the SPH, set to 10 minutes in this study[4–6], was defined according to the future clinical application. Since we are analysing scalp EEG data, the treatment strategies were limited to (i) acute drug administration to prevent an imminent seizure or (ii) the patient taking action to avoid accidents resulting from seizure occurrence. Rescue medication takes effect at most five to ten minutes after administration (as is the case of diazepam rectal gel)[7–9]. Treatment strategies requiring intracranial device implantation to deliver brain electrical stimulation typically define SPH intervals of a few seconds. However, given the considerable differences between scalp and invasive EEG, neurostimulation was not considered in our range of future applications[10,11]. We then defined a longer SPH interval of 10 minutes so that, when

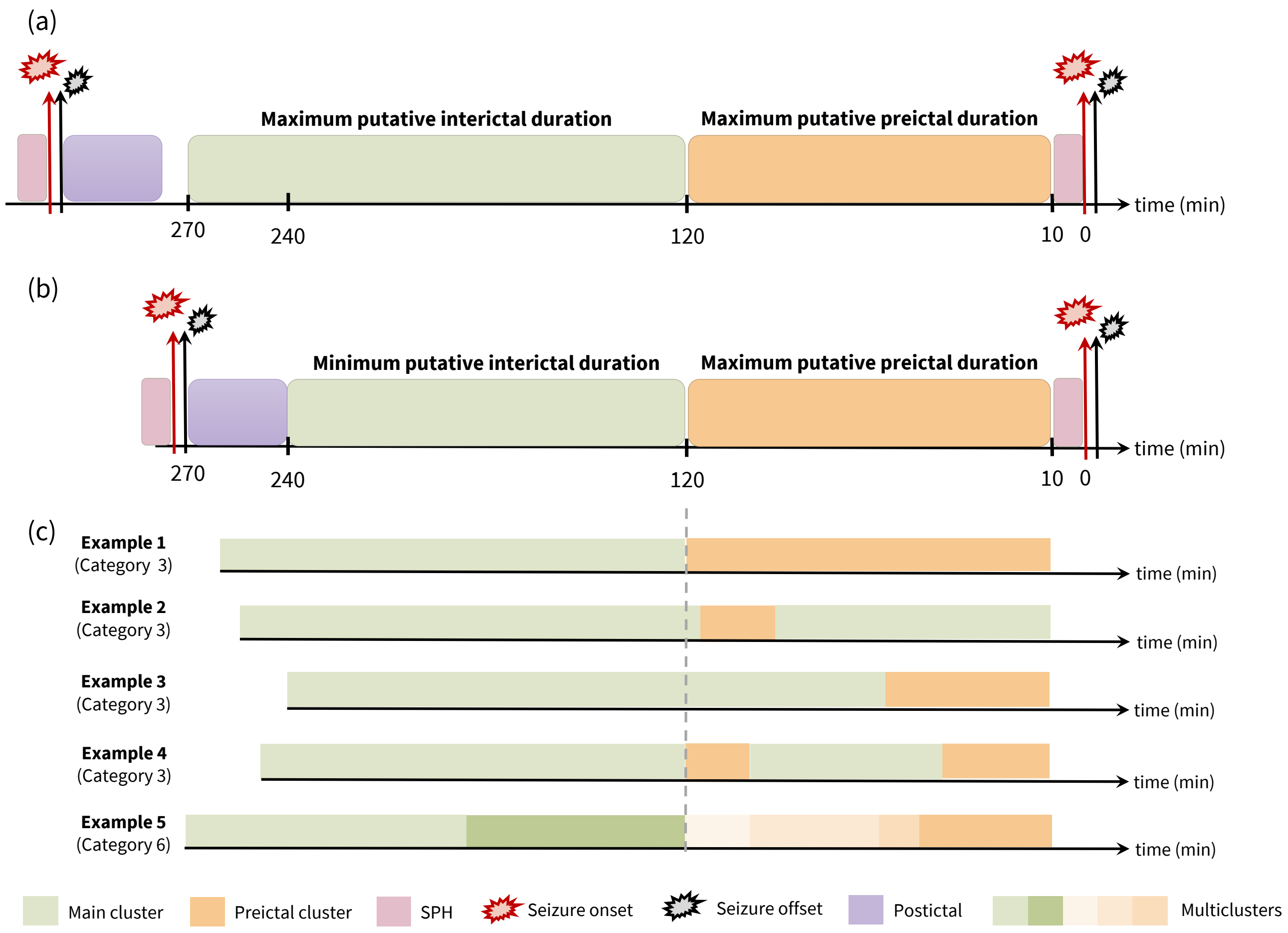

Fig. S1. Examples of preictal location and duration for the specific case of two seizures separated by (a) more than and (b) exactly 4.5 hours (270 minutes). Examples representing clustering solutions containing differently sized preictal clusters are presented in (c).

envisioning future studies, seizure prediction models enable the patient to have enough time, after receiving the alarm, to prepare for an upcoming seizure[11].

The following assumptions were considered when analysing the 4.5 hours of data recorded before an electrographic seizure onset:

(i) Seizures separated by at least 4.5 hours were considered independent events.

(ii) Although 4.5 hours (270 minutes) of data were analysed, only the electrographic changes observed within the 120- to 10-minute interval before the seizure onset were assumed to mainly represent the preictal state. Data locating before the 120-minute interval were considered as enough data to predominantly contain interictal data (see Fig. S1).

(iii) Given the higher probability of a preictal interval lasting less than an interictal interval, the cluster with the smaller number of samples represented the preictal interval (see Fig. S1).

(iv) This interval may not occur strictly near the seizure onset but could be captured as an EEG-related event eventually preceding the seizure onset (see Example 2 in Fig. S1).

2 Patient and seizure metadata

Table S1 contains information regarding the group of patients with temporal lobe drug-resistant epilepsy analysed in this study. Data were recorded while the patients were under presurgical monitoring, being submitted to medication withdrawal as the activation procedure[12]. The table includes information on sex, age at hospital admission and onset age (corresponding to the occurrence of the first epilepsy event), aetiology, epilepsy foci lateralisation, the total number of annotated seizures and the number of seizures analysed for each patient (lead seizures), according to the considered minimum inter-seizure interval of 4.5 hours. Information on aetiology is presented according to the International League Against Epilepsy (ILAE) nomenclature[13,14]. Most data were collected from patients with hippocampal sclerosis; even tough epilepsies with other structural causes, such as tumours and malformations of cortical development, have also been identified. Only one patient has immune aetiology, specifically, inflammation of the autoimmune-mediated central nervous system.

Table S2 contains a description of metadata annotated for each analysed seizure. This information, available in the EPILEPSIAE database[12,15], was annotated by experienced professionals by inspecting both video and EEG data. The table then includes information about seizure type, vigilance state determined 10 seconds before the seizure onset, and EEG onset time over the 24h period. Seizures were classified according to the ILAE nomenclature[16]. The vigilance state corresponds to one of the following states of alertness and responsiveness: wakefulness, non-rapid eye movement sleep (NREM sleep, further subdivided into three sleep stages N1–3) and rapid eye movement (REM) sleep[17]. Information about the seizure type, vigilance state and EEG onset time is also depicted in Fig. S2.

Additionally, Table S2 comprises information regarding the percentage of time during which noisy segments have been identified in each seizure's 4.5 hours of EEG. These noisy segments do not contain neurological information, but rather flat lines, saturated signal or abnormal peaks, for instance, caused by electrode detachment.

Given the extension of this table, we decided to provide here the information regarding the final preictal intervals identified for each seizure (either category 3 or 6), which was later used to perform preictal interval comparison among studies (refer to sections 6.5 and 6.6).

It is important to note that when preictal patterns were observed for more than one group of features, a final interval had to be chosen. The final preictal interval was chosen according to its characteristics. The first criterion was based on the preictal density as a preictal interval with higher density means that the vast majority (or even all) of the samples belong to the preictal state and there are fewer "jumps" to the remaining clusters. A preictal interval associated with a higher density means that a more evident and permanent change occurs before the seizure onset[18]. The second criterion, used when two preictal intervals had the same density, consisted in choosing a preictal interval based on the duration. In other words, for preictal intervals with different duration, we chose the one with the highest duration as it provided more statistical confidence in the presence of a preictal state[18]. Lastly, when after these two criteria, we still had two preictal intervals with the same density and duration; a third criterion was used to select the final interval. Namely, the third criterion consisted of choosing preictal intervals starting near the seizure onset. This means that the patient has to wait less time for the seizure to occur, reducing the impact of larger waiting times on the patient's anxiety levels.

Table S1: Dataset description regarding each patient.

Patient index	Patient identifier	Sex	Onset Age (years)	Admission Age (years)	Aetiology	Lateralisation	#Sz	#LSz
1	402	F	10	55	HS, inflammation	L, R	5	5
2	8902	F	23	67	Unknown	L	5	5
3	11 002	M	21	41	HS	R	8	4
4	16 202	F	43	46	Unknown	L, R	8	7
5	21 902	M	44	47	Malformation	L	6	4
6	23 902	M	36	36	Tumour	L	5	5
7	26 102	M	15	65	HS, malformation	L	8	4
8	30 802	M	28	28	Malformation	L, R	9	8
9	32 702	F	33	62	n. s.	L, R	6	5
10	45 402	F	13	41	HS	L, R	5	4
11	46 702	F	13	15	HS, malformation	R	5	5
12	50 802	M	2	43	HS	L	5	5
13	52 302	F	13	61	HS	L	7	4
14	53 402	M	0	39	HS	L, R	8	4
15	55 202	F	3	17	HS, malformation	R, B	9	8
16	56 402	M	18	47	Malformation	L, R	7	4
17	58 602	M	17	32	Tumour	L	22	6
18	59 102	M	17	47	Malformation	R	7	5
19	60 002	M	47	55	HS	L, R	8	6
20	64 702	M	3	51	Unknown	R	6	5
21	75 202	M	10	13	Tumour	R	8	7
22	80 702	F	14	22	Malformation	B	10	6
23	81 102	M	5	41	Unknown	R	13	3
24	85 202	F	4	54	HS	L	10	5
25	93 402	M	40	67	Unknown	L	7	5
26	93 902	M	43	50	n. s.	R	9	6
27	94 402	F	29	37	HS	R	11	7
28	95 202	F	13	50	HS	L	14	7
29	96 002	M	21	58	HS, malformation	L, R	9	7
30	98 102	M	2	36	Unknown	L	5	5
31	98 202	M	3	39	HS, malformation	R	10	7
32	101 702	M	44	52	HS	L, R	6	5
33	102 202	M	0	17	HS	L	28	7
34	104 602	F	8	17	n. s.	L	5	5
35	109 502	M	40	50	Unknown	L, R	10	4
36	110 602	M	6	56	HS	R	8	5
37	112 802	M	47	52	HS, malformation	L	6	6
38	113 902	F	16	29	HS	R	25	6
39	114 702	F	31	22	HS	R	25	8
40	114 902	F	15	16	HS	L, R	12	7
41	123 902	F	7	25	Tumour	L, R	8	5
Mean			19	41			9	6
SD			15	15			6	1
Total							388	226

Sex: female (F) or male (M). HS: hippocampal sclerosis. n. s.: not specified. Lateralisation: L: left, R: right, B: bilateral. #Sz: total number of seizures annotated per patient. #LSz: number of leading seizures, obtained as a result of the analysis of 4.5 hours of inter-seizure EEG data. SD: standard deviation.

Table S2: Dataset description regarding data preceding each lead seizure.

Seizure index	Patient identifier	EEG onset time	Vigilance state	ILAE Classification	Noise (%)	Starting time* (min)	Duration* (min)	Density* (%)
1	402	22:45:26	W	FOIA	3.1	111.5	17.9	94.91
2	402	21:27:34	W	FBTC	4.0	26.2	8.2	88.00
3	402	02:13:30	W	FOIA	9.3			
4	402	08:53:21	W	FBTC	10.8	73.3	56.5	98.37
5	402	08:57:27	W	FOIA	3.3	56.9	46.9	100.00
6	8902	23:51:14	W	UC	10.7			
7	8902	23:03:23	W	FOIA	5.4	65.1	55.1	98.49
8	8902	05:37:05	W	FOIA	2.1			
9	8902	00:35:56	W	FOIA	7.5			
10	8902	05:10:26	W	FOIA	0.0			
11	11 002	00:00:10	W	UC	7.1			
12	11 002	06:38:01	R	FOIA	0.1			
13	11 002	15:16:42	W	FOIA	19.3			
14	11 002	08:18:49	W	FOIA	9.3			
15	16 202	04:34:07	W	UC	0.0	35.8	25.8	100.00
16	16 202	06:05:10	W	FBTC	0.4			
17	16 202	05:07:14	W	UC	0.3	111.7	35.0	95.24
18	16 202	18:48:33	W	FOIA	11.2	21.4	4.4	100.00
19	16 202	03:34:35	W	FOIA	0.3			
20	16 202	13:50:31	W	FOIA	2.1	94.4	9.9	100.00
21	16 202	19:27:39	W	FOIA	5.9	22.8	9.9	82.50
22	21 902	16:16:43	W	UC	6.1			
23	21 902	08:40:51	W	FOIA	7.2	58.6	34.7	90.16
24	21 902	20:32:56	W	FOIA	9.2	45.4	34.5	84.53
25	21 902	06:50:12	R	FOIA	0.2			
26	23 902	10:18:13	W	FOA	10.9			
27	23 902	20:50:38	W	FOA	12.6	14.2	4.2	96.08
28	23 902	11:18:12	W	FOA	27.9			
29	23 902	16:48:02	W	FOA	6.8	20.1	4.3	94.23
30	23 902	22:17:22	W	FOA	11.0	25.6	7.1	94.25
31	26 102	15:31:37	W	FOIA	2.5			
32	26 102	08:33:50	W	FOIA	2.3			
33	26 102	07:52:54	W	FOIA	4.0			
34	26 102	11:36:45	W	FOIA	4.1	16.1	6.1	86.49
35	30 802	04:33:31	R	FOA	0.0			
36	30 802	04:52:24	W	FOA	3.5			
37	30 802	10:58:12	N2	FOA	10.6	63.5	53.5	95.80
38	30 802	22:58:11	W	FOA	2.5	89.8	9.8	84.75
39	30 802	05:49:34	W	FOA	0.0			
40	30 802	02:48:42	R	FOA	1.4			
41	30 802	07:48:06	N2	FOA	0.1			
42	30 802	03:15:10	N2	FOA	0.3			
43	32 702	08:25:28	W	FOIA	5.2			
44	32 702	10:22:47	W	FOIA	2.6			
45	32 702	10:13:13	W	FOIA	10.8	24.2	8.2	85.86
46	32 702	17:03:16	W	FOIA	20.0			
47	32 702	09:29:02	W	FOIA	4.2	90.5	14.7	98.83
48	45 402	01:48:55	W	FOIA	5.9			

Seizure vigilance state: wakefulness (W), NREM sleep stage I (N1), NREM sleep stage II (N2), REM sleep stage (R). Seizure ILAE classification: focal onset aware (FOA), focal onset impaired awareness (FOIA), focal to bilateral tonic-clonic (FBTC), unclassified (UC). Noise: percentage of time gap between the 10-minute preprocessed segments. *Values for the putative preictal intervals identified with unsupervised learning methods.

Continued on next page

Seizure index	Patient identifier	EEG onset time	Vigilance state	ILAE Classification	Noise (%)	Starting time* (min)	Duration* (min)	Density* (%)
49	45402	08:11:29	W	FOIA	3.8	54.9	44.9	95.00
50	45402	14:56:37	W	FOA	7.3			
51	45402	15:13:34	W	FOIA	4.8	67.8	10.0	95.00
52	46702	15:56:40	W	FOA	2.7			
53	46702	06:16:40	N2	FOIA	0.9			
54	46702	17:06:57	W	FOIA	4.0			
55	46702	02:02:23	N2	FBTC	4.1			
56	46702	06:45:59	W	FOIA	3.6			
57	50802	02:44:39	W	FOIA	0.3			
58	50802	06:37:35	N2	UC	0.2			
59	50802	12:39:04	N2	UC	3.5			
60	50802	22:50:41	N2	FOIA	7.5	30.7	20.4	97.93
61	50802	01:18:38	W	FBTC	0.5			
62	52302	06:29:39	W	UC	1.7			
63	52302	11:31:13	W	FOA	10.2			
64	52302	02:31:34	N1	UC	8.9			
65	52302	09:53:02	W	UC	15.0			
66	53402	08:16:32	W	FOA	2.8	23.8	8.8	100.00
67	53402	05:46:33	N2	FOA	2.9	18.0	4.7	81.03
68	53402	19:02:38	W	FOA	15.8	48.6	9.7	99.15
69	53402	09:17:43	W	FOIA	8.8			
70	55202	07:02:49	W	FOIA	0.2			
71	55202	09:55:11	W	FOIA	9.1			
72	55202	18:15:11	W	FOA	5.4	52.4	9.9	100.00
73	55202	08:09:27	W	UC	1.8	60.4	47.4	95.71
74	55202	17:47:47	W	UC	1.4			
75	55202	09:57:39	W	FOA	17.3	75.6	9.9	91.67
76	55202	15:34:54	W	UC	7.6			
77	55202	14:11:59	W	FOIA	7.8	18.2	8.2	92.93
78	56402	08:17:30	W	UC	3.8	23.1	9.2	100.00
79	56402	21:11:53	W	UC	4.5			
80	56402	09:13:46	W	UC	4.9	64.6	9.9	96.67
81	56402	06:29:39	W	FBTC	0.4			
82	58602	09:11:25	W	FOIA	4.8	28.9	18.9	100.00
83	58602	03:29:21	R	FOIA	3.5	79.5	69.5	96.53
84	58602	19:52:52	W	FOIA	4.3	58.9	9.9	98.33
85	58602	09:01:07	W	FOIA	0.6	29.8	19.8	99.16
86	58602	15:41:02	W	FOIA	7.8	17.5	7.5	63.33
87	58602	02:31:58	N2	FOIA	4.4	74.4	64.4	48.00
88	59102	08:54:51	W	FOA	10.5	110.7	100.7	99.10
89	59102	15:41:55	W	FOIA	27.2	40.6	24.0	80.97
90	59102	09:56:35	W	FOIA	13.6			
91	59102	19:51:41	W	FOIA	4.9			
92	59102	21:12:26	W	FOA	4.7	22.4	11.7	78.01
93	60002	02:45:01	N1	FOIA	0.0	46.6	36.6	92.97
94	60002	02:22:55	W	FOIA	2.7	29.7	19.7	86.50
95	60002	12:21:36	W	FOIA	5.4	43.4	13.5	66.87
96	60002	05:40:53	R	UC	0.4			
97	60002	00:17:54	R	FOIA	3.8			

Seizure vigilance state: wakefulness (W), NREM sleep stage I (N1), NREM sleep stage II (N2), REM sleep stage (R). Seizure ILAE classification: focal onset aware (FOA), focal onset impaired awareness (FOIA), focal to bilateral tonic-clonic (FBTC), unclassified (UC). Noise: percentage of time gap between the 10-minute preprocessed segments. *Values for the putative preictal intervals identified with unsupervised learning methods.

Continued on next page

Seizure index	Patient identifier	EEG onset time	Vigilance state	ILAE Classification	Noise (%)	Starting time* (min)	Duration* (min)	Density* (%)
98	60 002	22:18:46	N1	FOIA	1.5	28.1	11.7	74.65
99	64 702	13:53:39	W	FOA	7.4	41.3	9.9	98.33
100	64 702	04:23:21	W	FBTC	4.5	47.0	9.4	89.47
101	64 702	18:59:43	W	FBTC	6.8	32.3	9.9	90.00
102	64 702	19:50:01	W	FBTC	9.9	18.6	8.6	96.15
103	64 702	03:41:27	N2	FBTC	1.4			
104	75 202	23:37:38	N2	FOA	4.7	28.8	12.1	63.01
105	75 202	01:10:45	N2	FOA	6.9	92.8	5.3	87.69
106	75 202	21:33:44	W	UC	9.6	40.9	14.5	58.00
107	75 202	19:27:00	W	FOA	4.4			
108	75 202	09:46:19	W	FOA	13.3	18.3	7.7	80.65
109	75 202	17:43:46	W	FOA	15.5			
110	75 202	06:25:19	W	FOA	3.2	16.0	6.0	94.44
111	80 702	05:03:56	W	FOIA	0.0			
112	80 702	08:43:22	W	FOIA	0.3	35.9	9.9	98.33
113	80 702	20:43:38	W	UC	4.6	29.3	9.6	89.66
114	80 702	07:46:14	W	FOIA	2.1			
115	80 702	17:54:17	W	FBTC	3.9	97.3	11.1	100.00
116	80 702	08:53:56	W	FOIA	3.7	37.6	27.6	97.28
117	81 102	20:48:50	W	FOIA	3.6	61.3	51.3	98.35
118	81 102	10:44:57	W	FOA	8.3	24.3	11.7	87.14
119	81 102	10:42:15	W	FOIA	8.4	38.8	26.6	93.44
120	85 202	23:37:05	N2	FOIA	3.1			
121	85 202	16:51:04	W	FOIA	6.7	69.3	9.9	95.83
122	85 202	04:24:27	W	UC	0.0	50.3	40.3	96.69
123	85 202	16:08:00	W	UC	2.6	58.8	48.8	91.81
124	85 202	01:51:40	W	UC	1.3	34.8	24.8	98.66
125	93 402	22:17:50	N2	FBTC	4.3			
126	93 402	10:21:34	N2	FOIA	2.3			
127	93 402	23:20:24	N2	FOIA	6.4			
128	93 402	00:59:09	N2	UC	4.2	88.3	78.3	96.38
129	93 402	06:26:26	N2	UC	3.6			
130	93 902	08:39:52	W	FOA	1.4	36.8	26.8	99.03
131	93 902	16:02:21	W	FOIA	4.8	106.5	6.0	97.26
132	93 902	02:31:07	N2	FBTC	3.2	32.5	22.5	91.83
133	93 902	18:48:40	W	FOIA	21.8	25.1	11.9	87.41
134	93 902	04:02:38	N2	FOIA	0.2			
135	93 902	09:21:33	W	UC	10.8	64.7	10.1	98.36
136	94 402	15:29:22	W	FOA	3.9	29.2	6.4	66.67
137	94 402	11:02:56	W	UC	11.5	83.0	20.7	100.00
138	94 402	18:05:40	W	FOIA	5.7	29.7	9.9	100.00
139	94 402	01:36:02	N2	UC	1.8	119.9	109.9	98.63
140	94 402	16:10:53	W	FOA	8.5	18.7	8.7	96.19
141	94 402	02:48:18	N2	UC	4.2	32.8	22.7	98.90
142	94 402	08:16:30	W	FOA	2.9	94.8	76.3	99.89
143	95 202	01:28:09	N2	FBTC	11.0	29.6	19.6	99.15
144	95 202	15:00:18	N2	FOIA	8.7			
145	95 202	01:35:24	N2	FOIA	3.2			
146	95 202	14:13:22	N2	FOIA	3.3	24.5	9.8	88.24

Seizure vigilance state: wakefulness (W), NREM sleep stage I (N1), NREM sleep stage II (N2), REM sleep stage (R). Seizure ILAE classification: focal onset aware (FOA), focal onset impaired awareness (FOIA), focal to bilateral tonic-clonic (FBTC), unclassified (UC). Noise: percentage of time gap between the 10-minute preprocessed segments. *Values for the putative preictal intervals identified with unsupervised learning methods.

Continued on next page

Seizure index	Patient identifier	EEG onset time	Vigilance state	ILAE Classification	Noise (%)	Starting time* (min)	Duration* (min)	Density* (%)
147	95 202	23:30:29	N2	UC	9.1	21.8	11.8	86.62
148	95 202	23:55:21	N2	FOIA	5.9	38.8	28.8	89.88
149	95 202	00:04:20	N2	UC	11.2	52.2	42.2	97.83
150	96 002	17:10:35	W	FOIA	3.9	79.2	30.0	66.67
151	96 002	10:26:53	W	FOIA	11.7	18.5	8.5	98.06
152	96 002	17:46:44	W	FOIA	1.8	30.4	9.9	100.00
153	96 002	00:05:44	W	FOIA	5.7	79.8	69.8	96.06
154	96 002	00:44:10	W	UC	4.3	84.3	74.3	93.62
155	96 002	18:57:18	W	FOIA	1.3	49.6	9.9	100.00
156	96 002	06:20:01	W	FOIA	0.2	25.2	15.2	99.45
157	98 102	07:17:49	W	FOA	3.2			
158	98 102	18:49:53	W	UC	0.4	92.8	9.9	100.00
159	98 102	05:18:58	W	UC	1.3			
160	98 102	06:11:33	W	UC	2.1			
161	98 102	04:07:04	W	FBTC	2.7			
162	98 202	04:50:27	W	FOIA	4.5			
163	98 202	20:38:46	W	FOIA	6.0	45.4	7.6	94.62
164	98 202	07:16:40	W	FOIA	2.2			
165	98 202	12:16:11	W	FBTC	8.7	83.8	9.9	93.28
166	98 202	01:22:11	W	FOIA	3.9			
167	98 202	07:55:06	W	FOIA	6.4	24.9	9.9	99.17
168	98 202	16:57:19	W	UC	5.6			
169	101 702	07:35:40	W	FOIA	2.8			
170	101 702	12:29:53	W	FOIA	5.8	28.9	9.9	100.00
171	101 702	19:33:06	W	FOIA	3.6	120.0	87.9	66.53
172	101 702	07:35:22	N2	FOIA	4.4			
173	101 702	20:26:01	W	FOIA	3.5	115.9	47.1	44.52
174	102 202	22:50:21	N2	FOA	5.9	97.7	87.7	72.61
175	102 202	15:36:30	W	UC	10.5			
176	102 202	05:47:03	N2	FOIA	3.3			
177	102 202	22:14:59	W	UC	10.9	35.6	25.6	77.27
178	102 202	14:07:10	W	FOA	4.5			
179	102 202	06:16:20	N2	FOIA	0.9			
180	102 202	15:54:20	W	UC	7.5			
181	104 602	15:35:45	W	FOIA	18.8			
182	104 602	23:46:07	N2	FBTC	5.5			
183	104 602	06:24:56	N2	FBTC	0.3			
184	104 602	12:30:01	N2	FBTC	6.4			
185	104 602	22:44:07	N2	UC	8.6			
186	109 502	10:00:00	W	FOIA	14.5	23.2	9.0	52.29
187	109 502	19:42:33	W	FOIA	8.4	31.4	17.3	93.78
188	109 502	07:56:09	W	UC	1.6			
189	109 502	10:17:37	W	UC	16.2			
190	110 602	10:20:41	W	FOIA	6.3			
191	110 602	17:39:56	W	FOIA	3.7			
192	110 602	08:30:09	W	FOIA	8.5			
193	110 602	21:34:00	W	FOIA	3.1	29.6	16.3	89.29
194	110 602	11:28:35	W	FOA	15.4			
195	112 802	17:05:49	W	UC	1.6	36.1	9.8	54.62

Seizure vigilance state: wakefulness (W), NREM sleep stage I (N1), NREM sleep stage II (N2), REM sleep stage (R). Seizure ILAE classification: focal onset aware (FOA), focal onset impaired awareness (FOIA), focal to bilateral tonic-clonic (FBTC), unclassified (UC). Noise: percentage of time gap between the 10-minute preprocessed segments. *Values for the putative preictal intervals identified with unsupervised learning methods.

Continued on next page

Seizure index	Patient identifier	EEG onset time	Vigilance state	ILAE Classification	Noise (%)	Starting time* (min)	Duration* (min)	Density* (%)
196	112802	07:49:43	W	FOIA	6.5			
197	112802	15:36:04	W	UC	4.7			
198	112802	06:52:41	W	FOIA	0.0			
199	112802	11:54:45	W	FOIA	13.5			
200	112802	08:39:39	W	UC	6.5	38.6	19.9	98.75
201	113902	23:32:27	W	UC	6.4	37.1	25.2	89.97
202	113902	16:55:50	W	FOIA	6.6	23.4	9.9	98.33
203	113902	05:17:05	N2	FOIA	1.4			
204	113902	13:46:12	W	FOIA	9.2	27.1	17.1	66.99
205	113902	22:40:46	N2	UC	5.0	14.8	4.8	98.31
206	113902	16:53:42	W	FOIA	7.6	76.0	66.0	99.09
207	114702	20:52:30	W	FOIA	6.9			
208	114702	14:45:03	W	FOIA	14.6	29.9	10.4	93.60
209	114702	04:09:15	W	UC	0.0			
210	114702	09:50:10	W	FOIA	14.2	41.4	20.0	100.00
211	114702	14:27:45	W	FOIA	7.6			
212	114702	11:03:08	W	FOIA	4.8	116.0	9.9	96.67
213	114702	13:27:36	W	FOIA	3.5	77.6	11.6	91.43
214	114702	21:04:57	W	FOIA	2.3			
215	114902	08:30:29	W	FOA	9.0	43.8	33.8	75.48
216	114902	14:42:32	W	FOIA	2.1			
217	114902	19:42:40	W	FOIA	1.8			
218	114902	05:59:33	N2	FBTC	1.5			
219	114902	17:18:54	W	UC	6.8			
220	114902	11:52:26	W	FOIA	6.5			
221	114902	09:27:30	W	FOIA	9.2			
222	123902	02:52:47	N2	FBTC	0.0			
223	123902	01:38:19	N2	FBTC	1.3	45.3	35.3	94.58
224	123902	02:11:22	R	FOIA	3.0			
225	123902	18:57:10	W	FOIA	1.0	85.0	9.9	86.67
226	123902	15:22:45	W	FOIA	3.2			

Seizure vigilance state: wakefulness (W), NREM sleep stage I (N1), NREM sleep stage II (N2), REM sleep stage (R). Seizure ILAE classification: focal onset aware (FOA), focal onset impaired awareness (FOIA), focal to bilateral tonic-clonic (FBTC), unclassified (UC). Noise: percentage of time gap between the 10-minute preprocessed segments. *Values for the putative preictal intervals identified with unsupervised learning methods.

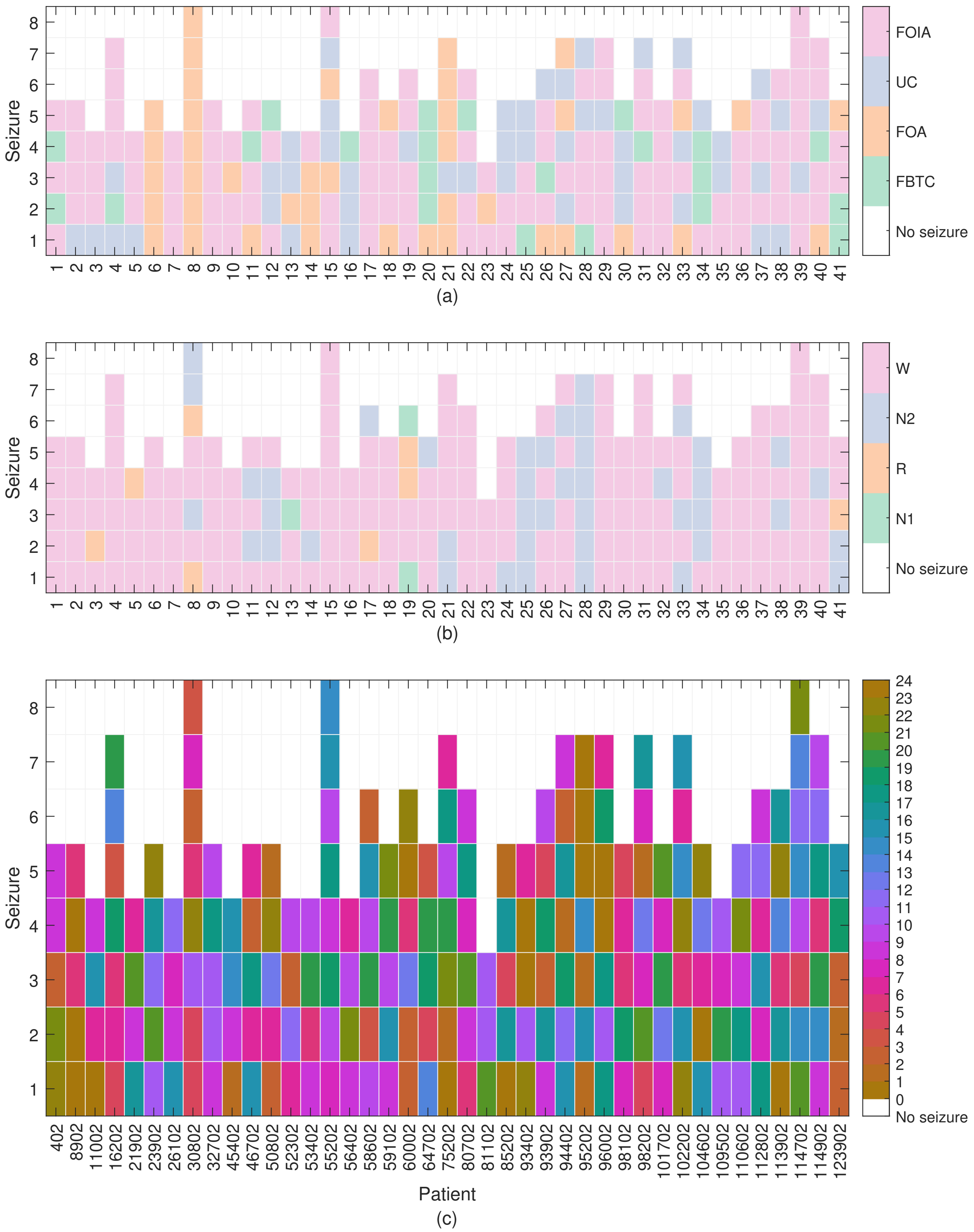

Fig. S2. Information regarding each seizure in the group of patients selected for this study. (a) Seizure ILAE classification: focal onset aware (FOA), focal onset impaired awareness (FOIA), focal to bilateral tonic-clonic (FBTC) and unclassified (UC). (b) Seizure vigilance state: W: wakefulness, R: REM sleep stage, N1: NREM sleep stage I, N2: NREM sleep stage II. (c) Seizure onset hour across the 24-hour day.

3 EEG feature engineering

There is a vast amount of literature spanning the different groups of features typically extracted from EEG. The next subsections describe the univariate linear, univariate nonlinear and multivariate features considered in this study according to the state of the art[19,20]. The frequency bands considered in univariate linear and multivariate feature extraction comprise delta (0.5 to <4 Hz), theta (4 to <8 Hz), alpha (8 to <13 Hz), beta (13 to <30 Hz), and gamma (30 to <47 Hz)[19–21]. The upper limit of the gamma band was defined to mitigate the impact of muscle artefacts in the analysis. Contrarily to intracranial EEG, with scalp EEG, it is more difficult to accurately capture lower amplitude faster oscillations characteristic of beta and gamma bands. Additionally, the large number of muscle artefacts in scalp EEG overshadows the real quantification of gamma rhythms due to the overlapping frequency spectrum[22,23]. During the EEG preprocessing step, there were 10-minute segments for which brain information was maintained at the cost of not removing some of the artefacts (mainly muscle artefacts). As such, spectral information above 47 Hz was left out of the analysis to reduce the impact of muscle artefacts in the results.

3.1 Univariate linear features

A total of 42 features were extracted from each 5-second window, and each EEG channel[19–21].

3.1.1 Time-domain univariate linear features

Statistical measures

Five statistical measures were considered in this study: normalised and non-normalised (with respect to the maximum value in each window) mean amplitude[5], standard deviation, skewness, and kurtosis[19,24].

These measures are meant to capture information regarding the amplitude distribution of a given time series. Symmetric and asymmetric amplitude distributions translate to zero and non-zero skewness, respectively. The relative flatness (or peakedness) of the amplitude distribution is reflected in the value of kurtosis[20].

Hjorth parameters

Hjorth parameters, activity, mobility and complexity, were conceptualised as clinically useful tools to quantitatively describe the EEG[20]. Activity is given by the variance of a given time series, $y(t)$:

$$Activity = var(y(t)) \tag{1}$$

Mobility corresponds to the variance of the slopes of a time series normalised by the variance of that time series:

$$Mobility = \sqrt{\frac{var(y'(t))}{var(y(t))}} \tag{2}$$

Complexity quantifies the variance of the rate of slope changes of a time series with reference to an ideal sine curve. The more similar the time series is to a pure sine wave, the more approximate will be the value of complexity to 1.

$$Complexity = \frac{Mobility(y'(t))}{Mobility(y(t))} \tag{3}$$

Decorrelation time

The decorrelation time corresponds to the time at which the first zero-crossing of the autocorrelation function occurs. The samples in a time series are less correlated as the time of the first zero-crossing approaches zero. The decorrelation time is, therefore, an indicator of signal periodicity. Considering the extreme case of a white noise signal, it theoretically presents a zero value of decorrelation time[25,26].

3.1.2 Frequency-domain univariate linear features

The frequency spectrum was computed using Welch's power spectral density estimate (see Fig. S3 (c)-(d)). Five frequency bands were considered: delta (0.5-4 Hz), theta (4-8 Hz), alpha (8-13 Hz), beta (13-30 Hz), and gamma (30-47 Hz)[19]. We extracted the power and the relative power in each of those frequency bands. The relative power corresponds to dividing the power in each of these frequency bands by the total power of the time series[19,25–27].

- Spectral power in each frequency band (delta, theta, alpha, beta, and gamma).
- Total power.

- Relative spectral power in each frequency band (delta, theta, alpha, beta, and gamma).
- Alpha peak frequency.
- Spectral edge frequency and power (at 50%).
- Mean frequency.
- Power ratios between frequency bands [delta/alpha, delta/beta, delta/gamma, delta/theta, theta/alpha, theta/beta, theta/gamma, alpha/beta, alpha/gamma, beta/gamma, beta/(alpha+theta), and theta/(alpha+beta)].
- Energy of wavelet coefficients obtained using discrete wavelet transform and Daubechies (db4) mother wavelet at five levels of decomposition (detail coefficients D1: 64-128 Hz, D2: 32-64 Hz, D3: 16-32 Hz, D4: 8-16 Hz, D5: 4-8 Hz, and approximation coefficient A5: 0-4 Hz).

Spectral edge frequency and power

Typically, the power spectrum of an EEG signal is characterised by a predominance of power in the 0 to 40 Hz frequency band. The spectral edge frequency, f_{50}, is a measure of the power spectrum distribution that consists of the minimum frequency for which it is possible to obtain 50% of the spectral power up to 40 Hz, P_{40Hz}[20].

$$f_{50} = min\left\{ f* \,\middle|\, \sum_{f=0Hz}^{f*} p_f > P_{40Hz} \cdot 0.50 \right\} \tag{4}$$

The spectral edge power corresponds to the power spectrum area below the spectral edge frequency[25,26,28].

Energy of wavelet coefficients

The wavelet transform has been used as an alternative to the FFT analysis as it allows for multiresolution time-frequency decomposition. Particularly, the discrete wavelet transform (DWT) decomposes a given time series into approximation and detail coefficients yielding the first level of decomposition. The approximation coefficients in every level are further decomposed into the next level of approximation and detail coefficients. The DWT coefficients are obtained by applying the mother wavelet to a given time series at different translations and scales. The first levels correspond to the time series' high-frequency content, whereas the last levels contain low frequencies[25,29–31]. Given that the dataset under analysis contains data sampled at 256 Hz, we applied the DWT decomposition using Daubechies (db4)[25,26,30] mother wavelet at five decomposition levels: detail coefficients D1 (64-128 Hz), D2 (32-64 Hz), D3 (16-32 Hz), D4 (8-16 Hz), D5 (4-8 Hz), and approximation coefficient A5 (0-4 Hz)[32]. At last, we computed the energy of each decomposition level and of the last approximation level[25].

3.2 Univariate nonlinear features

A total of 29 univariate nonlinear features were extracted from each 5-second window and EEG channel:

Higuchi's fractal dimension

Higuchi's fractal dimension (HFD) is a fractal measure of the irregularity and self-similarity of a given signal[31,33,34]. HFD corresponds to the slope of the linear fit between a log-log plot of the length and different scales of a given 5-second EEG window. This feature requires the definition of a free parameter (k_{max}) corresponding to the maximum number of scales that have been analysed. In our study, this parameter was set to 100 as a result of an estimation process that evaluated a range of k_{max} values and assessed when the corresponding values of fractal dimension reached a plateau[33–35].

Monofractal detrended fluctuation analysis

Monofractal detrended fluctuation analysis (DFA) was performed to explore the extent of long-range correlations in the EEG 5-second windows for different time scales[36,37]. Two scaling exponents were returned from DFA analysis: (i) DFA slope α_1 ($DFA\ \alpha_1$) corresponding to short-term fluctuations, within the 10-32 sample range and (ii) DFA slope α_2 ($DFA\ \alpha_2$) corresponding to long-term fluctuations, over the 32-128 sample range[36].

Multifractal detrended fluctuation analysis

Multifractal detrended fluctuation analysis (MFDFA) is a generalisation of the DFA method, which is computed for a single scale or fractal dimension. This method explores the possibility that different fractal patterns may describe the fractal structure of the EEG segments. Three measures were obtained from the multifractal spectrum: width, the abscissa value of the apex, and the asymmetry parameter[36,38,39]. The asymmetry parameter measures the multifractal spectrum symmetry: a symmetric spectrum corresponds to a zero value of the asymmetry parameter; an asymmetric spectrum that is left- or right-skewed yields a positive or negative value of the asymmetry parameter[40].

Multifractal 1-D Wavelet Leader estimates

Multifractal 1-D Wavelet Leader estimates is an alternative method based on wavelet analysis to estimate the multifractal spectrum[41]. We extracted 11 measures characterising the multifractal spectrum,[41–44] as can be seen in Fig. S3 (e)-(f). An asymmetrical spectrum is obtained when the structure of the time series is not sensitive to the local fluctuations with (i) large magnitudes (long right tail) or (ii) small magnitudes (left long tail). When the time series contains high and low fluctuation components presenting a similar scaling complexity, the multifractal spectrum takes a symmetrical shape[36,43].

Approximate and sample entropies

Approximate and sample entropies quantify the regularity and the complexity of a time series[45–47]. By inspecting these features, it is possible to know the likelihood that similar sequences found for m points, within a tolerance, r, will also be found for $m+1$ points. Both measures require the definition of the number of points, m, comprised in the sequences further compared, and the tolerance value, r, for which matches are accepted. Parameter m is equal to 2[46]. The tolerance, taken as the similarity criterion, was set to $0.2 \times SD$, SD corresponding to the standard deviation of the 5-second EEG window (of length N)[46,48–50].

Correlation dimension and largest Lyapunov exponent

The correlation dimension (CD) and the largest Lyapunov exponent (LLE) were obtained from the reconstruction of the underlying m-dimensional dynamical system. The former quantifies the complexity of a system, whereas the latter provides information regarding the overall predictability of that system, i.e., the evolution of the trajectories in the phase space. Periodic signals are associated with null values of LLE, whereas chaotic systems display increased values of the LLE[51]. Similarly, an increase in complexity corresponds to an increase in the value of CD[51]. These measures were obtained through the reconstruction of the underlying system's phase space. In this study, the two parameters required for phase space reconstruction, the embedding dimension, m, and the time delay, τ, were estimated (for each 5-second window) using the False Nearest Neighbour algorithm and the first local minimum of the average mutual information method, respectively (refer to Fig. S4)[52–54]. The LLE was then obtained using the Rosenstein *et al.* (1993)[55] method, which is one of the most widely used for this purpose, whereas CD was computed based on the Grassberger & Procaccia (1983)[56] method.

Recurrence quantification analysis

A recurrence quantification analysis (RQA) provides information regarding hidden periodicities in the aforementioned phase space trajectory. Such information can be accessed by computing the recurrence plot (RP) of a given time series[57]. The first step to obtaining such representation is to compute the square matrix with dimensions $N \times N$ (known as the colour recurrence plot), containing the pairwise Euclidean distance (given by the norm $||\cdot||$) between all samples of the trajectory, N, in the m-dimensional space. Afterwards, a threshold distance ε is used to define a sphere centred at the state x_i. If x_j falls within that sphere, then the Heaviside function $\Theta(\cdot)$ decides for $R_{i,j} = 1$, meaning the states are close to each other. Otherwise, $R_{i,j} = 0$. This binary matrix, symmetric along the identity line, can therefore be visualised in a black ($R_{i,j} = 1$) and white plot ($R_{i,j} = 0$), the RP. In this study, the value of ε was estimated for each 5-second non-overlapping window, corresponding to 10% of the maximum phase space diameter[58]. The RQA returned seven RP measures of complexity, which quantify recurrence point density and indicate the existence of diagonal and/or vertical lines in the RP[58,59]. The following RQA features were computed:

- Recurrence rate (REC).
- Determinism (DET).
- Average diagonal line length (L).
- Length of the longest diagonal line (L_{max}).
- Laminarity (LAM).

- Trapping time (TT).
- Shannon entropy (ENT).

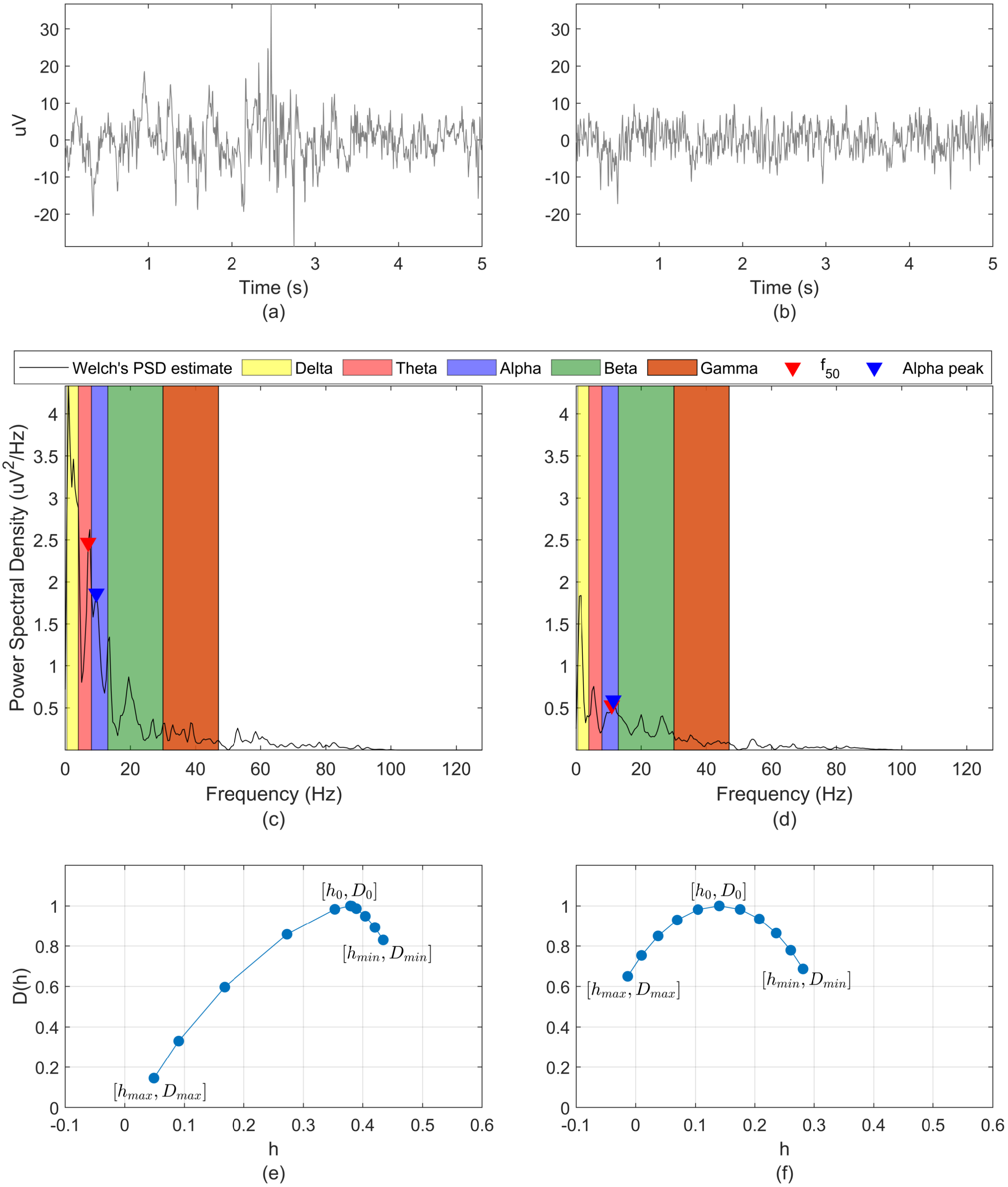

Fig. S3. Example of two 5-second EEG windows located (a) 4.5 hours and (b) 5 minutes before the onset of the first seizure of patient 402. The frequency spectrum (c)-(d) and the multifractal spectrum (e)-(f) have been obtained for both windows. The multifractal spectrum has been computed using the `dwtleader` Matlab function. Eleven measures were saved for each 5-second window: h_{min}, h_{max}, D_{min}, D_{max}, h_0, spectrum width $\Delta h = h_{max} - h_{min}$, $\Delta D = D_{max} - D_{min}$, $h_0 - h_{min}$, $h_{max} - h_0$, $D_0 - D_{max}$, and $D_0 - D_{min}$.

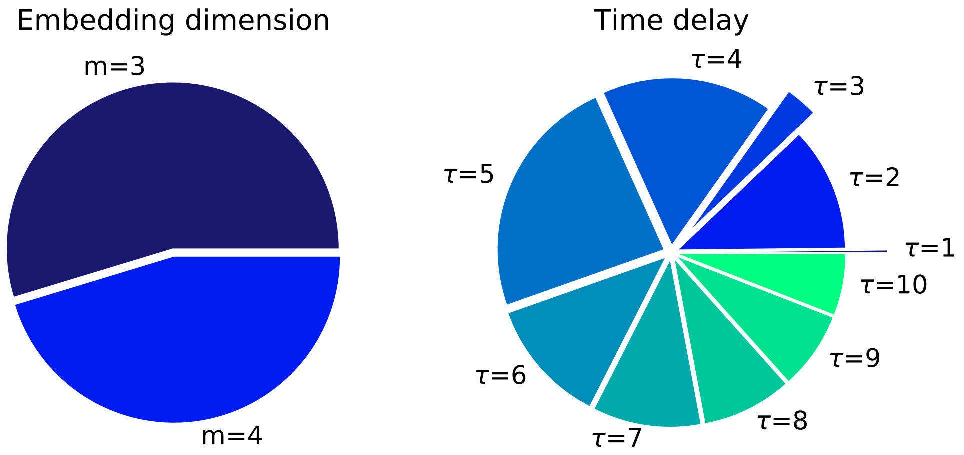

Fig. S4. Frequency of phase space reconstruction parameters in the present study. The embedding dimension, m, and the time delay, τ, were estimated for each 5-second window using the False Nearest Neighbour algorithm and the first local minimum of the average mutual information method.

3.3 Multivariate features

A total of 495 multivariate features were computed and are described below. First, we computed bivariate measures from all EEG channel pairs. Then we extracted graph measures from the obtained connectivity matrices.

Undirect connectivity measures (per frequency band)

- Circular omega complexity[60].
- Circular correlation[60].
- Intersite phase clustering[61,62].
- Phase lag index[61–64].
- Weighted phase lag index[62–64].
- Debiased weighted phase lag index[62,63].
- Spearman's correlation coefficient[62].
- Spearman's correlation coefficient for instantaneous power[62].
- Normalised cross-correlation[24,64–66].
- Normalised cross-correlation for instantaneous power[24,65,66].

Direct connectivity measures (per frequency band)

- Phase slope index[62,63].

Graph indexes for undirect connectivity measures

With the exception of circular omega complexity (which is already a multivariate measure of connectivity), all the remaining bivariate features were analysed using the following graph measures. These are measures of local and global connectivity[62,64,67,68]:

- Assortativity (A).
- Characteristic path length (CPL).
- Global efficiency (GE).
- Modularity (M).

- Mean network degree (MD).
- Mean strength (MS).
- Mean closeness centrality (MCC).
- Mean betweenness centrality (MBC).
- Transitivity (T).
- Mean weighted clustering coefficient (WGCC).

Graph indexes for direct connectivity measures

The following graphs measures were extracted from the connectivity matrix of the phase slope index bivariate feature[67]:

- Assortativity (A).
- Characteristic path length (CPL).
- Global efficiency (GE).
- Modularity (M).
- Mean strength (MS).
- Mean betweenness centrality (MBC).
- Mean incloseness centrality (MCIC).
- Mean outcloseness centrality (MCOC).

3.4 Feature significance in seizure prediction studies

Most of the univariate linear features described above have been widely used in the context of seizure prediction. For instance, statistical measures have been shown to significantly change during the preictal period compared to the interictal state[19,24,27,28]. The preictal interval has been associated with a decrease in the variance, and an increase in the kurtosis[19]. Hjorth parameters (mobility and complexity) were also reported to increase during the preictal interval[24]. The decorrelation time was documented to decrease near the seizure onset[19]. Pinto *et al.* concluded that Hjorth mobility and skewness were highly discriminating features, often selected by their evolutionary seizure prediction model[6].

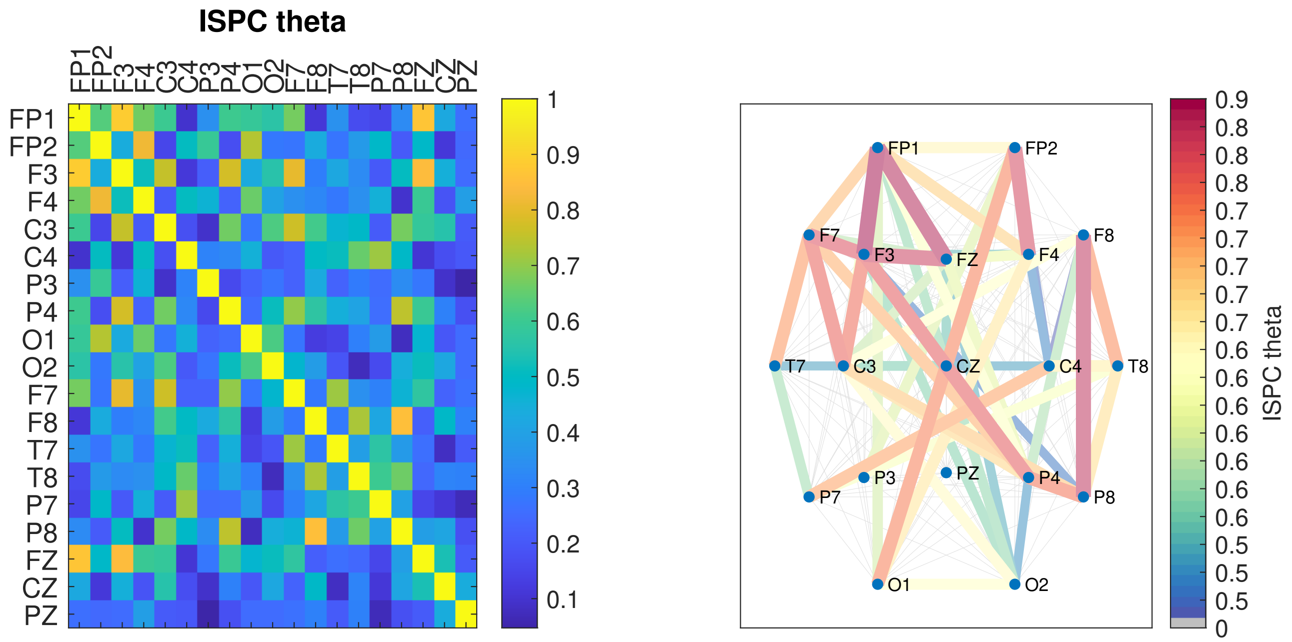

Fig. S5. Connectivity matrix obtained for theta band intersite phase clustering for the 5-second EEG window located 4.5 hours before the onset of the first seizure of patient 402. Grey edges indicate non-significant interaction strengths.

Extracting frequency-domain univariate linear features has been extensively performed in seizure prediction[19]. Mormann *et al.*[24] showed a decrease in delta band power during the preictal period, accompanied by a relative increase of power in the other subbands. Park *et al.* documented gamma frequency bands to be the most discriminating features when classifying interictal and preictal samples[69]. Pinto *et al.* reported theta band relative power and mean normalised frequency as the most frequently extracted features by their seizure prediction model. Spectral edge frequency at 75% of the spectrum was later reported by the same authors to arise as a highly discriminating feature in seizure prediction[6].

Additionally, univariate nonlinear features have been investigated in seizure prediction. The largest Lyapunov exponent, correlation dimension, fractal dimension, recurrence quantification analysis, and approximate and sample entropies are frequently considered to develop prediction models[19,20,31,47,70,71]. Mormann *et al.*[24] observed an increase of the largest Lyapunov exponent 30 minutes before seizure onset. In this study, we extracted different measures of fractal dimension. Higuchi's fractal dimension has already been used in the context of seizure prediction[31,35]. The detrended fluctuation analysis (DFA) has been mainly used to measure the long-range correlation of the electrocardiogram[72]. In epilepsy, DFA has often been considered to identify the disease characteristics[73,74] and for the noninvasive localisation of the epileptic zone before presurgical evaluation[75]. More recently, some attempts have been documented on the use of DFA in seizure prediction studies[39,76]. Initially, the monofractal perspective on the EEG oscillations was typically addressed by a monofractal DFA analysis. However, the nonstationary nature of EEG demands a multifractal DFA that contemplates the existence of large and small fluctuations[40,73]. Besides adding a multifractal DFA, we also considered the method of multifractal 1-D Wavelet Leader estimates. This decision was supported by the simplicity of the methods' implementation (we used the `dwtleader` Matlab function) associated with the potential of simultaneously characterising multifractal properties and exploring wavelet self-similarity structures[41].

Even though uncertainty exists as to whether nonlinear features bring discriminatory power or not, there are some studies indicating that higher prediction performance can be obtained by combining linear and nonlinear features[77]. Moreover, a method that is able to capture the nonlinear nature of EEG signals may provide valuable insight into brain dynamics[31].

The inspection of multivariate features in this study is supported by some studies reporting increased prediction performance when using bivariate features, in comparison with univariate linear features[24,78]. Although we are analysing multivariate features, these are global measures computed from graphs of bivariate measures. It is important to note that using bivariate measures as input to the feature reduction methods would result in a considerable increase in the feature reduction computational time (as a total of 7695 bivariate features would be analysed).

Ultimately, when studies conducted a feature selection step in the seizure prediction methodology, high inter-patient variability is often observed[28,78]. This further motivates the inclusion of different types of features in our study.

4 EEG feature data preparation

After feature extraction, we inspected the feature datasets obtained for each feature group (univariate linear, univariate nonlinear, and multivariate) and each seizure. Specifically, we searched for constant and quasi-constant features in the three feature groups. Constant (corresponding to features that have the same value for all 5-second windows) and quasi-constant features (corresponding to features for which more than half of the values are equal) were discarded from further analysis.

Given that we searched for constant and quasi-constant features for each seizure independently, the feature dataset after discarding constant and quasi-constant features could differ among seizures. That was only verified for the multivariate feature group. In fact, no constant features were found for univariate linear and univariate nonlinear features. Alpha peak frequency, decorrelation time and spectral edge frequency (at 50%) were the quasi-constant features removed over all channels and seizures from the univariate linear feature group. The remaining univariate linear features were selected (not discarded) over all channels and seizures. RQA feature L_{max} was the only quasi-constant feature found across all channels for the univariate nonlinear feature group. The remaining univariate nonlinear features were selected over all channels and seizures.

For the multivariate feature group, we observed that the feature dataset (resulting after discarding constant and quasi-constant features) would differ from seizure to seizure. Based on that, we provide information regarding the constant, quasi-constant and features that showed across all seizures in Table S3. We counted a total of 15 (3.0%), 111 (22,4%), and 216 (43.6%) constant, quasi-constant, and selected (over all channels and seizures) multivariate features, respectively.

Table S3: Information regarding the constant, quasi-constant and selected (not discarded over all channels and seizures) multivariate features.

Feature	Graph index	Delta	Theta	Alpha	Beta	Gamma
Circular omega complexity	—					
Circular correlation	A					
	CPL					
	GE					
	M					
	MBC					
	MCC					
	MD					
	MS					
	T					
	WGCC					
Spearman's correlation coefficient	A					
	CPL					
	GE					
	M					
	MBC					
	MCC					
	MD					
	MS					
	T					
	WGCC					
Spearman's correlation coefficient for instantaneous power	A					
	CPL					
	GE					
	M					
	MBC					
	MCC					
	MD					
	MS					
	T					
	WGCC					
Normalised cross-correlation	A					
	CPL					
	GE					
	M					
	MBC					
	MCC					
	MD					
	MS					
	T					
	WGCC					
Normalised cross-correlation for instantaneous power	A					
	CPL					
	GE					
	M					
	MBC					
	MCC					
	MD					
	MS					
	T					
	WGCC					

Continued on next page

Feature	Graph index	Delta	Theta	Alpha	Beta	Gamma
Phase lag index	A	selected				
	CPL	selected	selected	selected	selected	selected
	GE	selected				
	M	selected				
	MBC	quasi-constant	quasi-constant	quasi-constant		
	MCC	quasi-constant				
	MD	quasi-constant	quasi-constant	quasi-constant		
	MS	selected				
	T	quasi-constant	quasi-constant			
	WGCC	quasi-constant	quasi-constant			
Weighted phase lag index	A	selected	selected	selected		
	CPL	selected	selected	selected	selected	selected
	GE	selected	selected	selected		
	M	selected	selected			quasi-constant
	MBC	quasi-constant	quasi-constant	quasi-constant	quasi-constant	quasi-constant
	MCC	selected				quasi-constant
	MD	quasi-constant	quasi-constant	quasi-constant	quasi-constant	quasi-constant
	MS	selected	selected	selected		
	T	selected	selected		quasi-constant	quasi-constant
	WGCC	selected	selected		quasi-constant	quasi-constant
Debiased weighted phase lag index	A	selected			quasi-constant	quasi-constant
	CPL	selected	selected	selected	selected	selected
	GE	selected			quasi-constant	quasi-constant
	M	selected			quasi-constant	quasi-constant
	MBC	quasi-constant	quasi-constant	quasi-constant	quasi-constant	quasi-constant
	MCC	selected			quasi-constant	quasi-constant
	MD	quasi-constant	quasi-constant	quasi-constant	quasi-constant	quasi-constant
	MS	selected			quasi-constant	quasi-constant
	T	selected				
	WGCC	selected				
Intersite phase clustering	A	selected	selected	selected	selected	selected
	CPL	selected	selected	selected	selected	selected
	GE	selected	selected	selected	selected	selected
	M		selected	selected	selected	selected
	MBC	quasi-constant	quasi-constant	quasi-constant	quasi-constant	quasi-constant
	MCC	quasi-constant	selected	selected	selected	
	MD	quasi-constant	quasi-constant	quasi-constant	quasi-constant	quasi-constant
	MS	selected	selected	selected	selected	selected
	T	selected	selected	selected	selected	
	WGCC	selected	selected	selected	selected	
Phase slope index	A	selected	selected	selected	selected	selected
	CPL	selected	selected	selected	selected	selected
	GE	selected	selected	selected	selected	selected
	M	selected	selected	selected	selected	selected
	MS	selected	selected	selected	selected	selected
	MBC	constant	constant	constant	constant	constant
	MCIC	constant	constant	constant	constant	constant
	MCOC	constant	constant	constant	constant	constant

Constant (■), quasi-constant (■) and selected (■) multivariate features.

5 Unsupervised learning methods

The following methods were chosen to search for the preictal interval in the EEG-based feature dataset:

1. K-means clustering, a widely used clustering partitioning method, is better suited to detect well-separated and similarly sized and shaped clusters[79,80]. The initial cluster centroids were selected using the *k-means++* method[81] and the distances between points and centroids were computed using the Euclidean distance. The algorithm was run for k clusters, with $k = 2,3,4$.

2. Agglomerative hierarchical clustering can identify structured clusters. We used the Ward linkage method and the Euclidean distance as the dissimilarity measure[82]. The algorithm was run for k clusters, with $k = 2,3,4$.

3. Hierarchical density-based spatial clustering of applications with noise (HDBSCAN) performs DBSCAN over varying values of ε, which is an input argument that defines the maximum distance that can exist between two points within the same cluster[83]. HDBSCAN automatically selects the optimal clustering solution, requiring the definition of the number of samples in a neighbourhood, $MinPts$, for a point to be considered a core point[84,85]. This parameter was set to six, corresponding to twice the dimensionality of the feature space[86]. An optional input parameter, minimum cluster size, $MinSz$, was set to 20 samples[18]. Density-based clustering algorithms can be used to identify arbitrarily shaped clusters[83,85,86].

4. Expectation-maximisation clustering using Gaussian mixture models[87], besides successfully identifying round clusters, is also used to find elongated clusters that follow Gaussian distributions. Mean and standard deviation are estimated using the expectation-maximisation algorithm. The algorithm was run for k mixture components (or underlying Gaussian distributions), with $k = 2,3,4$.

6 Results for unsupervised learning

This section presents the results obtained after performing feature dimensionality reduction and clustering tasks.

6.1 Results for clustering solution categorisation

Categorisation performed by each of the five members comprising our epilepsy research team is presented for multivariate (Table S4), univariate linear (Table S5), univariate nonlinear (Table S6), and control univariate linear reduced data (Table S7).

Fig. S6 depicts the number of seizures for which less than 3, 3, 4, or 5 votes have been observed after the categorisation task. When less than 3 votes would be obtained for a given seizure, the expert team would gather and discuss over the category that should be assigned to that seizure. Importantly, the figure also shows the number of seizures that were assigned a given category with the vote of Expert 1, who performed the first visual inspection. Fig. S7 depicts the number of categories that resulted from this team discussion.

Table S4: Multivariate reduced data categorisation by the five experts.

Expert	Category 1	Category 2	Category 3	Category 4	Category 5	Category 6
1	106 (46.9%)	11 (4.9%)	23 (10.2%)	1 (0.4%)	77 (34.1%)	8 (3.5%)
2	89 (39.4%)	8 (3.5%)	22 (9.7%)	0 (0.0%)	94 (41.6%)	13 (5.8%)
3	84 (37.2%)	8 (3.5%)	24 (10.6%)	10 (4.4%)	86 (38.1%)	14 (6.2%)
4	106 (46.9%)	8 (3.5%)	23 (10.2%)	2 (0.9%)	68 (30.1%)	19 (8.4%)
5	66 (29.2%)	37 (16.4%)	24 (10.6%)	0 (0.0%)	52 (23.0%)	47 (20.8%)
**Final categorisation**	**104 (46.0%)**	**9 (4.0%)**	**22 (9.7%)**	**1 (0.4%)**	**79 (35.0%)**	**11 (4.9%)**

Table S5: Univariate linear reduced data categorisation by the five experts.

Expert	Category 1	Category 2	Category 3	Category 4	Category 5	Category 6
1	34 (15.0%)	27 (11.9%)	18 (8.0%)	4 (1.8%)	58 (25.7%)	85 (37.6%)
2	42 (18.6%)	21 (9.3%)	25 (11.1%)	7 (3.1%)	58 (25.7%)	73 (32.3%)
3	34 (15.0%)	16 (7.1%)	30 (13.3%)	10 (4.4%)	66 (29.2%)	70 (31.0%)
4	38 (16.8%)	25 (11.1%)	32 (14.2%)	5 (2.2%)	54 (23.9%)	72 (31.9%)
5	14 (6.7%)	10 (4.8%)	15 (7.2%)	3 (1.4%)	32 (15.4%)	134 (64.4%)
**Final categorisation**	**38 (16.8%)**	**21 (9.3%)**	**19 (8.4%)**	**5 (2.2%)**	**61 (27.0%)**	**82 (36.3%)**

Table S6: Univariate nonlinear reduced data categorisation by the five experts.

Expert	Category 1	Category 2	Category 3	Category 4	Category 5	Category 6
1	81 (35.8%)	40 (17.7%)	23 (10.2%)	4 (1.8%)	58 (25.7%)	20 (8.8%)
2	87 (35.8%)	25 (11.1%)	26 (11.5%)	13 (5.8%)	56 (24.8%)	19 (8.4%)
3	67 (29.6%)	28 (12.4%)	25 (11.1%)	18 (8.0%)	69 (30.5%)	19 (8.4%)
4	85 (37.6%)	27 (11.9%)	31 (13.7%)	6 (2.7%)	54 (23.9%)	23 (10.2%)
5	53 (23.5%)	50 (22.1%)	19 (8.4%)	3 (1.3%)	48 (21.2%)	53 (23.5%)
**Final categorisation**	**79 (35.0%)**	**32 (14.2%)**	**26 (11.5%)**	**5 (2.2%)**	**63 (27.9%)**	**21 (9.3%)**

Table S7: Control univariate linear reduced data categorisation by the five experts.

Expert	Category 1	Category 2	Category 3	Category 4	Category 5	Category 6
1	18 (38.3%)	13 (27.7%)	5 (10.6%)	4 (8.5%)	5 (10.6%)	2 (4.3%)
2	24 (51.1%)	10 (21.3%)	6 (12.8%)	2 (4.3%)	0 (0.0%)	5 (10.6%)
3	19 (40.4%)	10 (21.3%)	9 (18.1%)	4 (8.5%)	2 (4.3%)	3 (6.4%)
4	19 (40.4%)	10 (21.3%)	10 (21.3%)	4 (8.5%)	2 (4.3%)	2 (4.3%)
5	25 (53.2%)	6 (12.8%)	9 (19.1%)	2 (4.3%)	3 (6.4%)	2 (4.3%)
**Final categorisation**	**21 (44.7%)**	**10 (21.3%)**	**8 (17.0%)**	**4 (8.5%)**	**2 (4.3%)**	**2 (4.3%)**

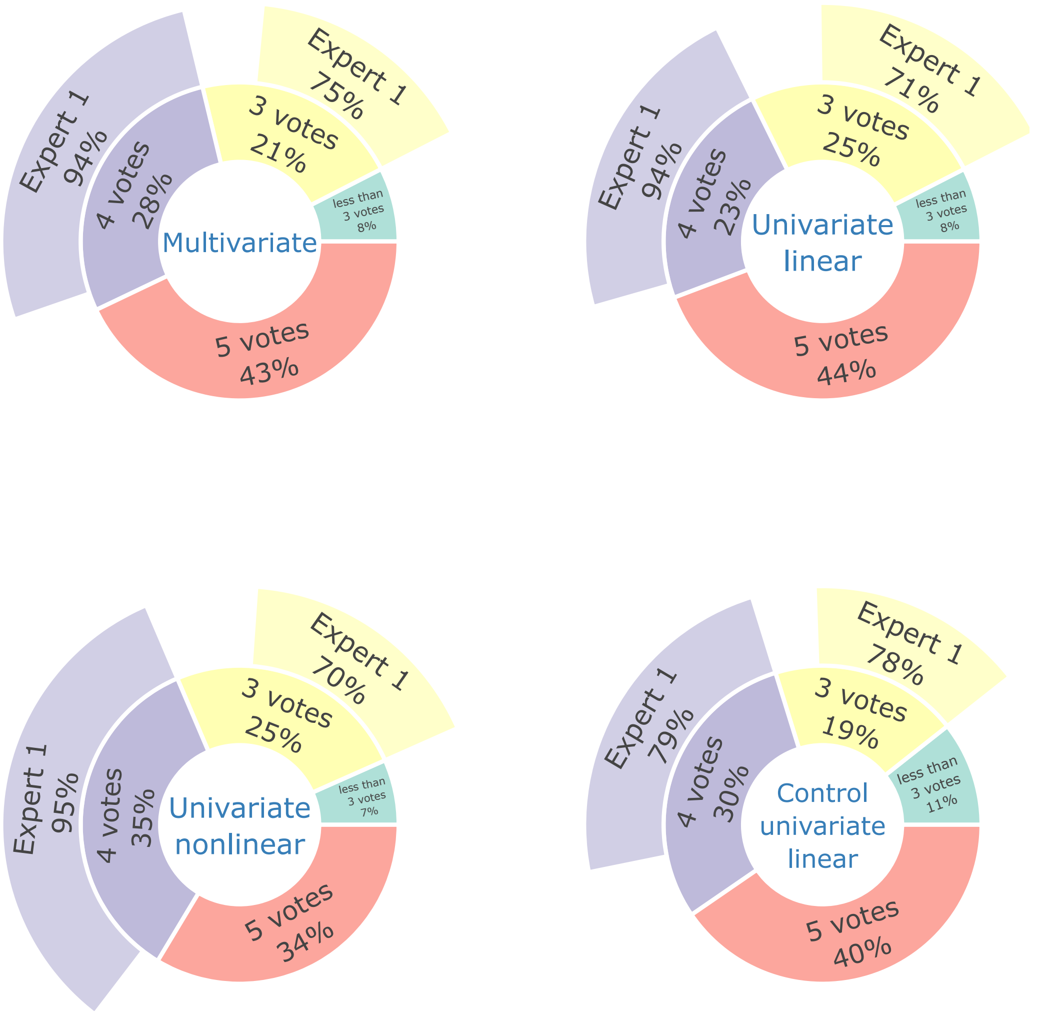

Fig. S6. Number of votes obtained for each seizure and each feature group after the categorisation task. The number of seizures for which the final category was assigned with the vote of Expert 1 (involved in the first inspection of the data) is depicted.

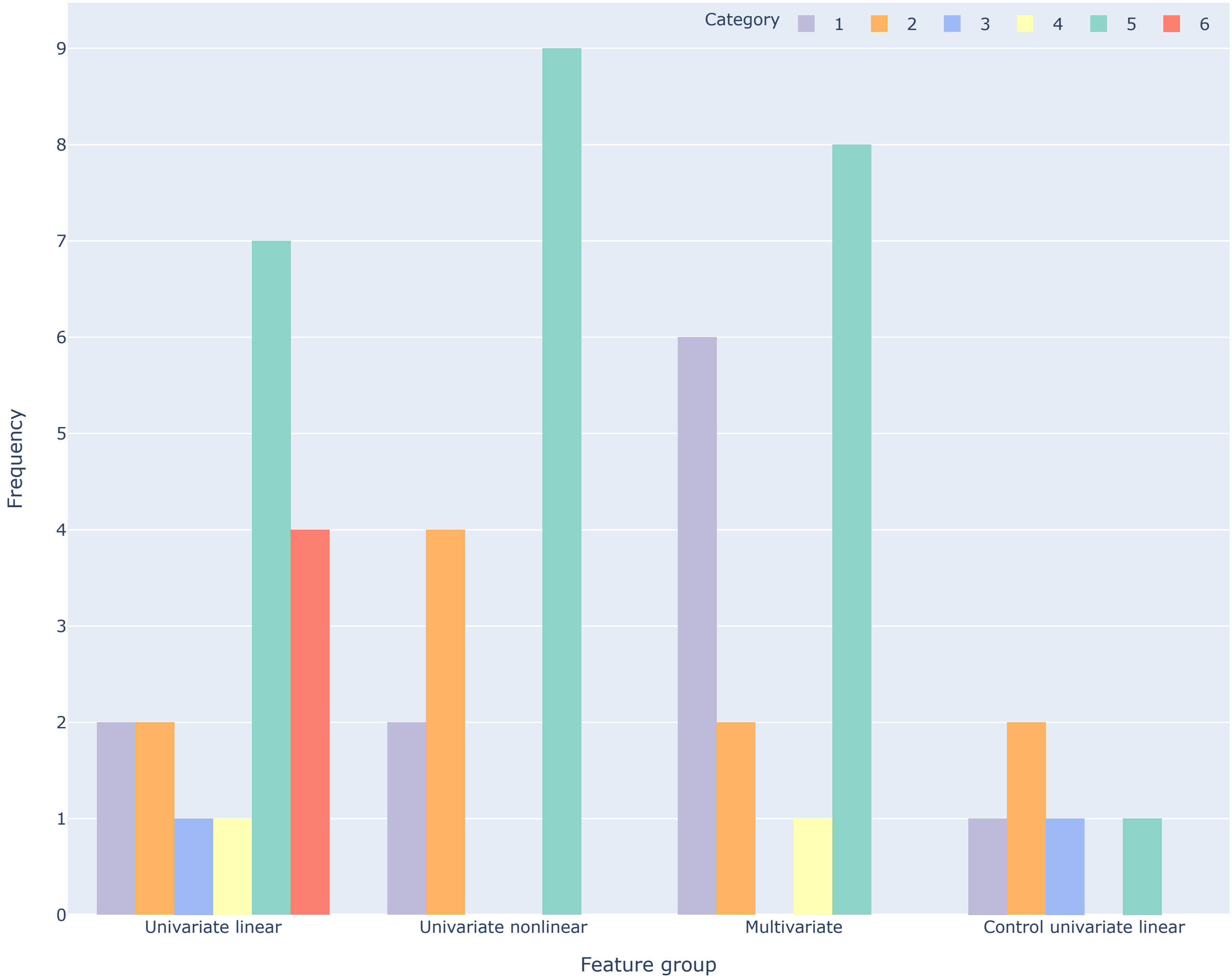

Fig. S7. Number of categories assigned after team member discussion over the categorisation for which less than three votes were obtained.

6.2 Results for control intervals

In this section we present the results for the analysis of control intervals. Figures S8 present some examples of seizure distribution para certain patients. The lead seizures, the analysed 4.5 hours of data preceding seizures and the control intervals are depicted. Figures for all patients are provided on the GitHub page.

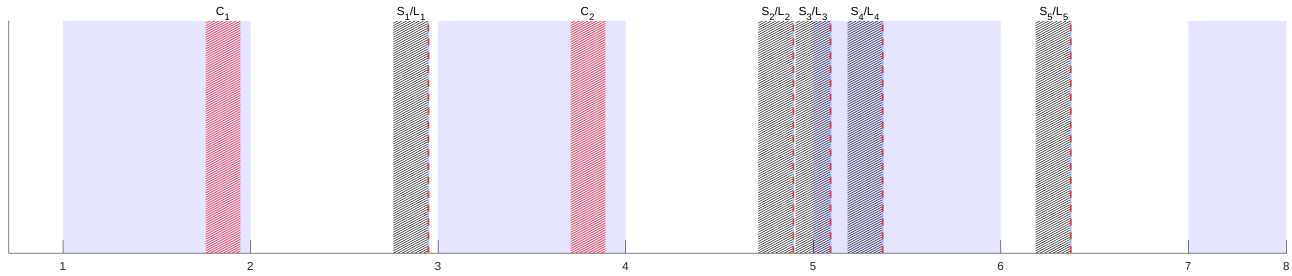

(a)

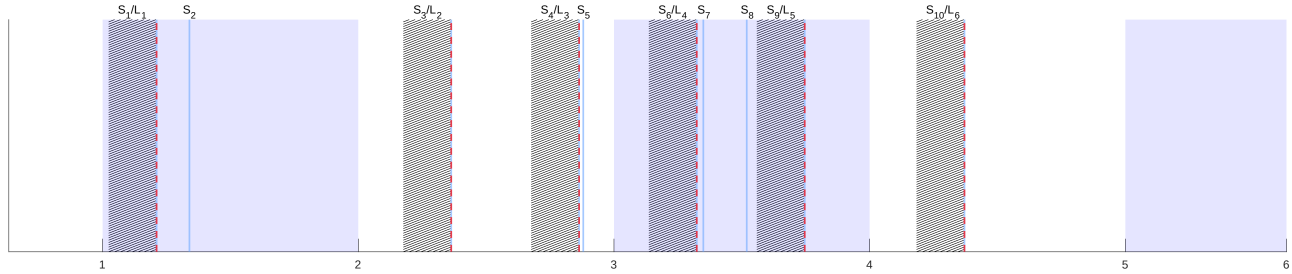

(b)

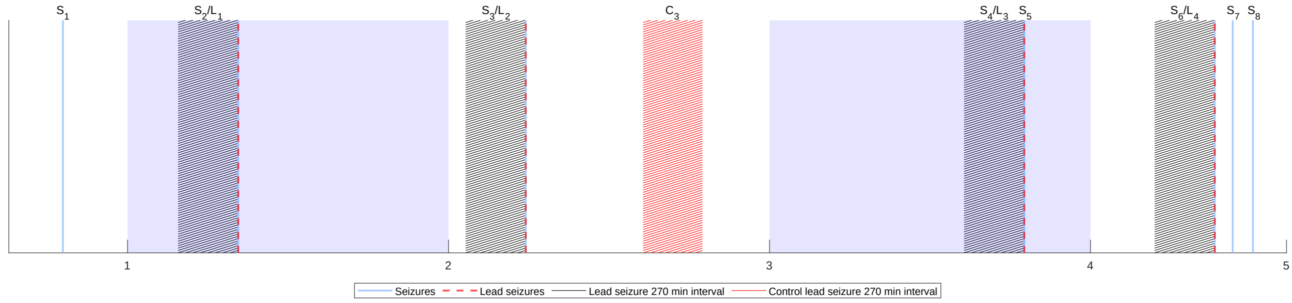

(c)

Fig. S8. Seizure distribution for patient (a) 402, (b) 80702 and (c) 53402. White and purple rectangles identify each subsequent day. In (a) the 4.5-hour interval was analysed for all seizures while only two 4.5-hour control intervals were analysed. In (b) the 4.5-hour interval was analysed for six seizures while no 4.5-hour control intervals were analysed. In (c) the 4.5-hour interval was analysed for four seizures while only one 4.5-hour control interval was analysed.

Table S8 shows the categories assigned to the two 4.5-hour intervals analysed for each seizure: the one preceding the onset and the control interval. This analysis was conducted for the univariate linear feature group. The seizures categorised as 3 or 6 in the 4.5-hour interval preceding the onset are indicated in bold.

Table S8: Categorisation results for the 4.5 hours of data before seizure and for the corresponding control interval.

	Patient index	Seizure index	Category	Category control		Patient index	Seizure index	Category	Category control
**1**	**402**	**1**	**6**	**1**	25	93902	1	5	2
**2**	**402**	**2**	**3**	**1**	26	93902	2	1	1
3	8902	1	5	4	**27**	**93902**	**3**	**6**	**3**
4	11002	1	5	1	**28**	**94402**	**1**	**6**	**1**
5	21902	1	5	1	**29**	**94402**	**2**	**6**	**2**
6	23902	3	2	1	**30**	**95202**	**1**	**6**	**2**
7	30802	1	5	2	**31**	**95202**	**7**	**6**	**3**
8	32702	1	5	1	**32**	**96002**	**7**	**6**	**6**
9	45402	1	1	4	**33**	**98102**	**2**	**3**	**3**
10	50802	1	1	1	34	98102	3	5	1
11	50802	3	5	1	35	98202	1	4	1
12	52302	1	5	1	**36**	**98202**	**2**	**6**	**1**
**13**	**53402**	**3**	**3**	**5**	**37**	**102202**	**1**	**6**	**6**
14	56402	2	1	1	**38**	**109502**	**1**	**6**	**3**
**15**	**56402**	**3**	**3**	**2**	39	109502	4	5	2
**16**	**58602**	**1**	**6**	**3**	40	110602	1	5	1
**17**	**58602**	**2**	**3**	**3**	41	110602	3	4	2
**18**	**59102**	**5**	**6**	**1**	**42**	**112802**	**1**	**6**	**1**
**19**	**60002**	**1**	**6**	**1**	43	112802	4	1	5
**20**	**64702**	**2**	**6**	**2**	44	112802	6	4	3
**21**	**75202**	**1**	**3**	**3**	**45**	**113902**	**1**	**6**	**4**
22	85202	1	5	4	46	123902	1	2	1
23	93402	1	5	1	47	**123902**	**2**	**6**	**4**
**24**	**93402**	**4**	**3**	**2**					

6.3 Visual representation of preictal characteristics

Figures S9, S10, S11 and S12 display a visual representation of the results for the preictal identification using unsupervised learning.

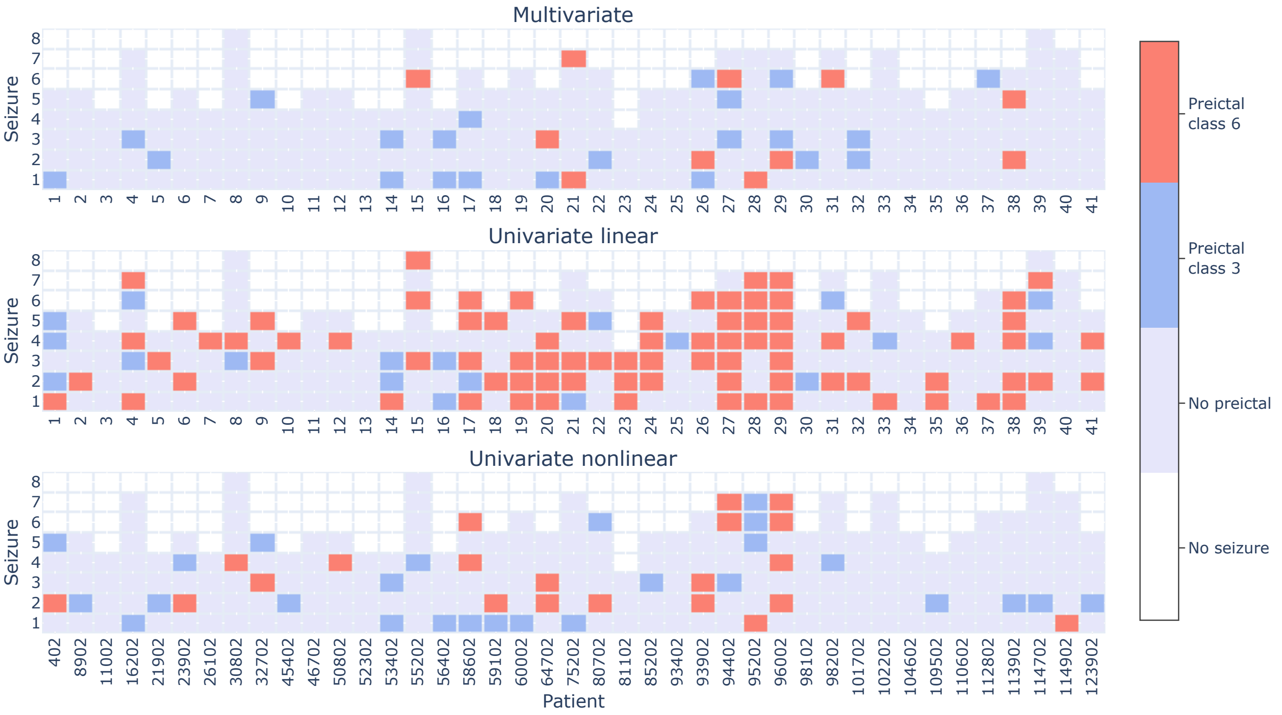

Fig. S9. The results correspond to the evidence of preictal interval found for classes 3 and 6, for the three groups of features. Regarding multivariate features, the preictal interval was found for 20 patients (49%) and for 33 seizures (15%). Regarding univariate linear features, the preictal interval was found for 36 patients (88%) and for 101 seizures (45%). Regarding univariate nonlinear features, the preictal interval was found for 29 patients (71%) and for 47 seizures (21%). Multivariate features provided evidence for preictal interval for an additional four seizures, when comparing to univariate features.

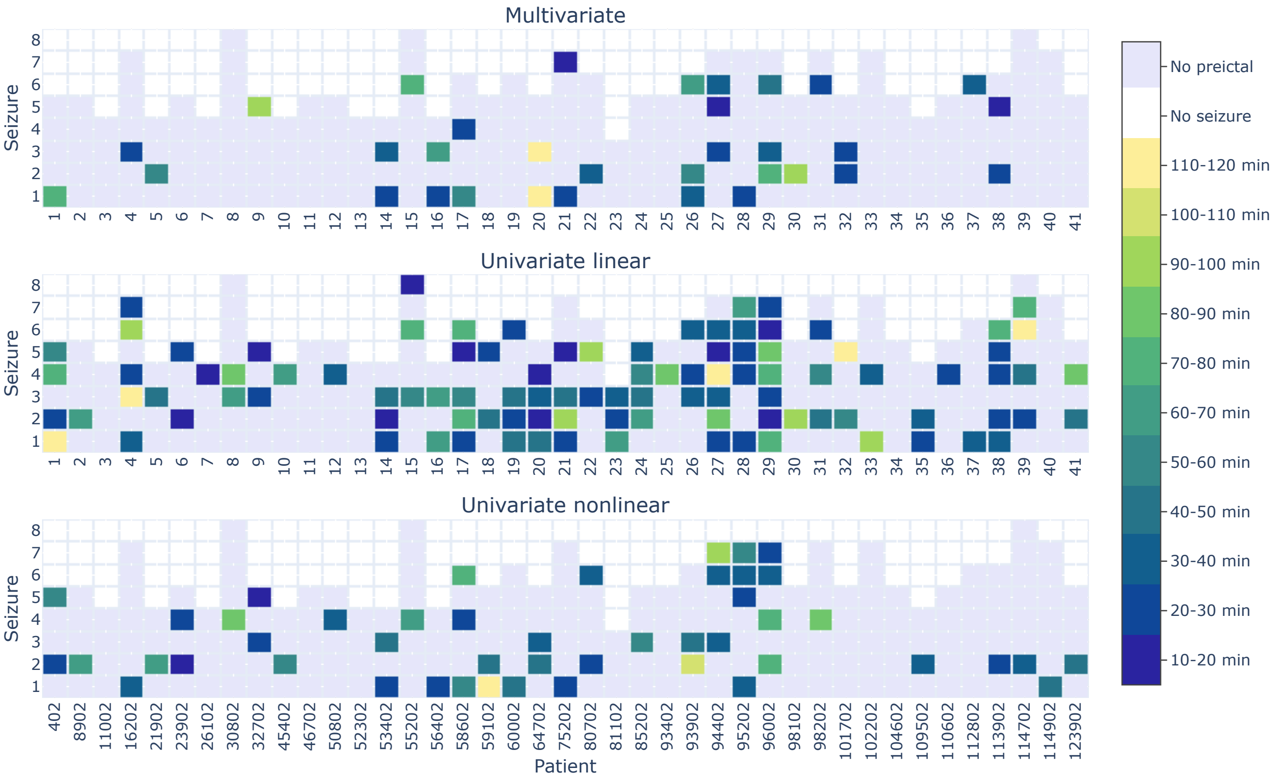

Fig. S10. Preictal starting time before seizure onset across patients and seizures.

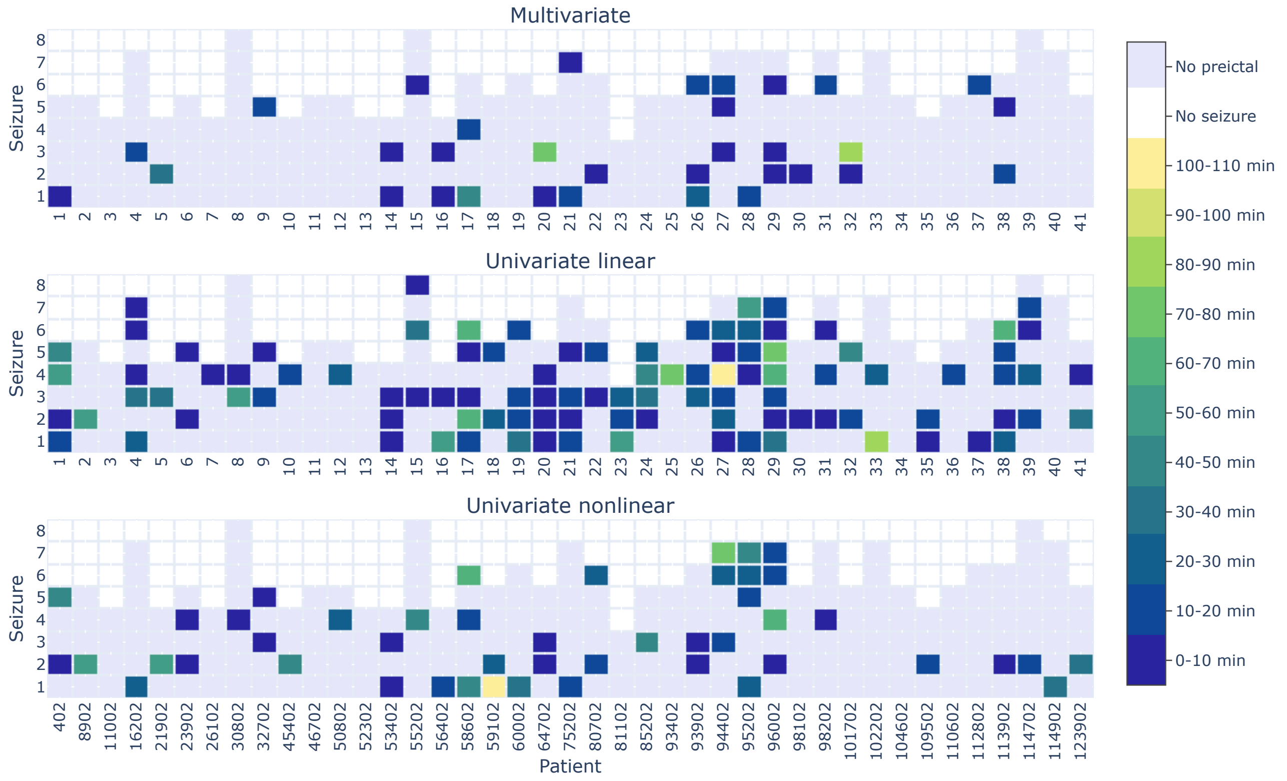

Fig. S11. Preictal duration across patients and seizures.

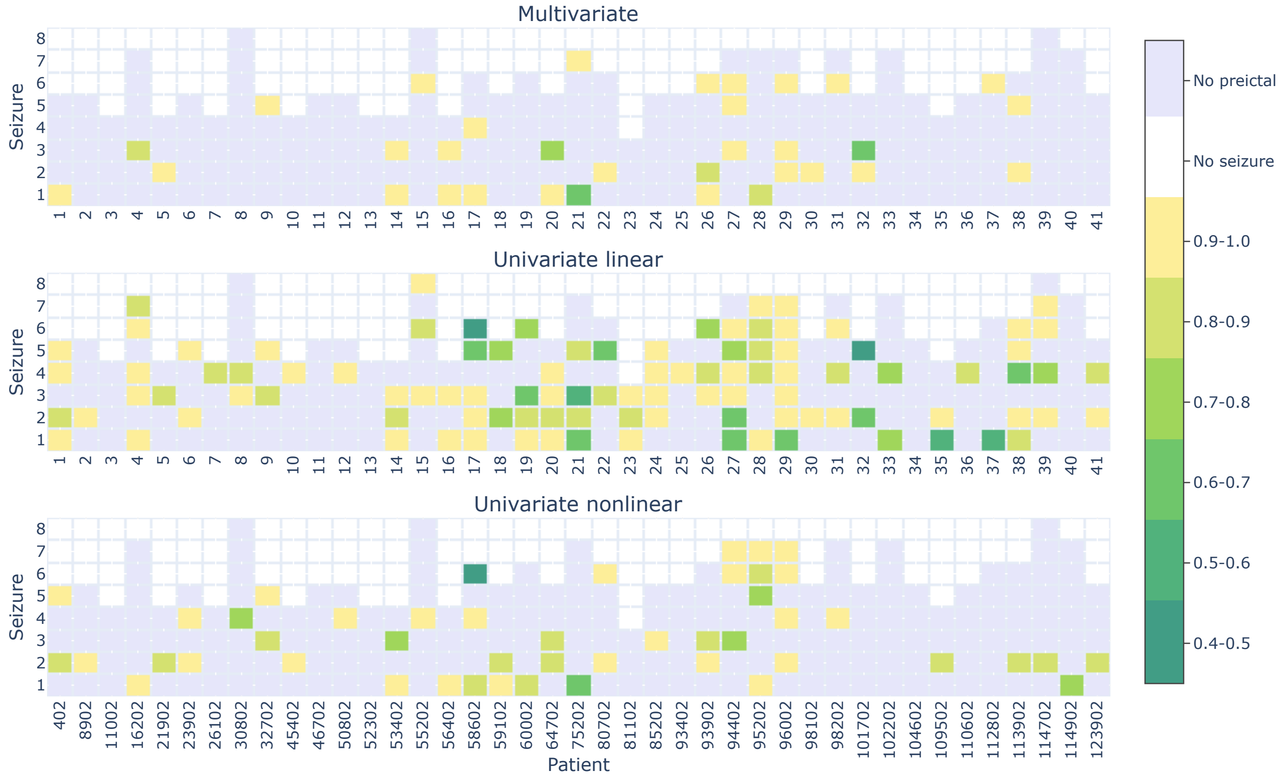

Fig. S12. Preictal cluster density across patients and seizures.

6.4 Prevalence of clustering methods

Fig. S13 presents the prevalence of the clustering methods explored in this study. We assessed the frequency of each method for each group of features. The analysis was conducted considering all categories and only categories 3 and 6 (categories representing putative preictal activity).

Fig. S14 presents the mean and standard deviation of the Dunn's index computed across seizures, for each feature group, category, and clustering method. We also present the values for all categories. Fig. S15 presents a similar analysis, this time, for the number of clusters (mean and standard deviation across seizures) for each feature group, category, and clustering method.

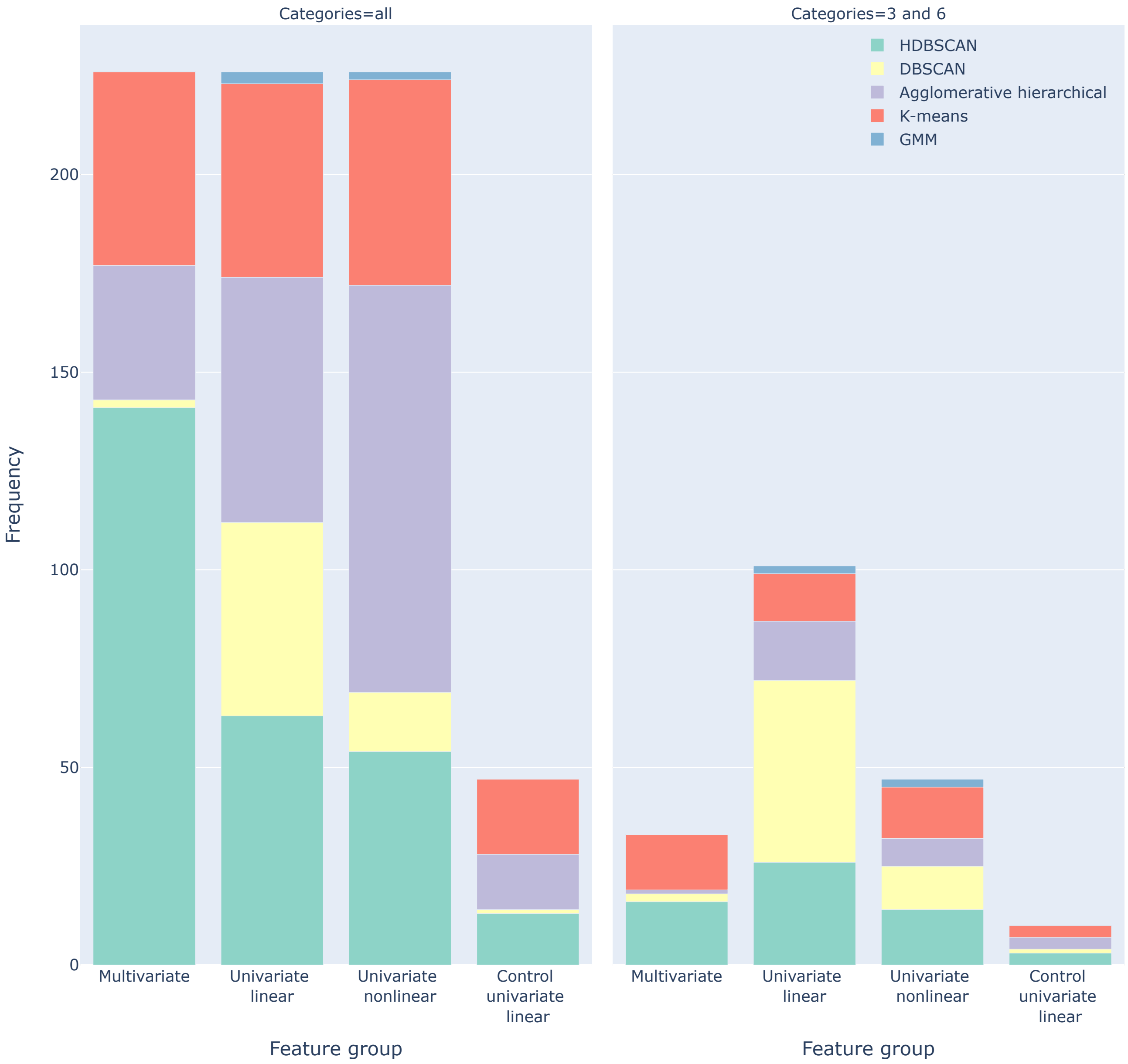

Fig. S13. Frequency of each clustering method computed for each feature group, for all categories and for only categories 3 and 6.

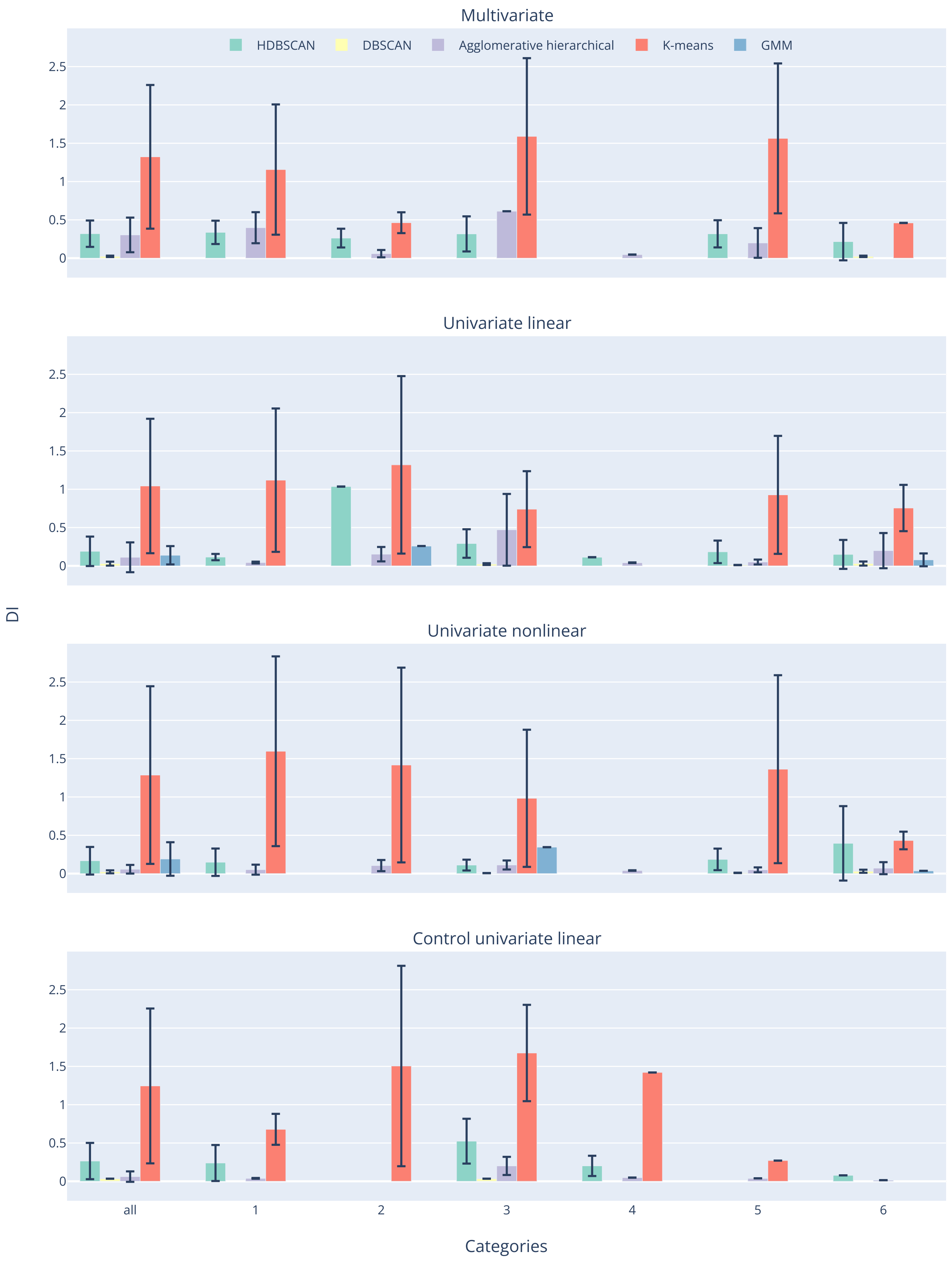

Fig. S14. Mean and standard deviation of Dunn's index computed for each feature group, each clustering method, and for all categories and each category.

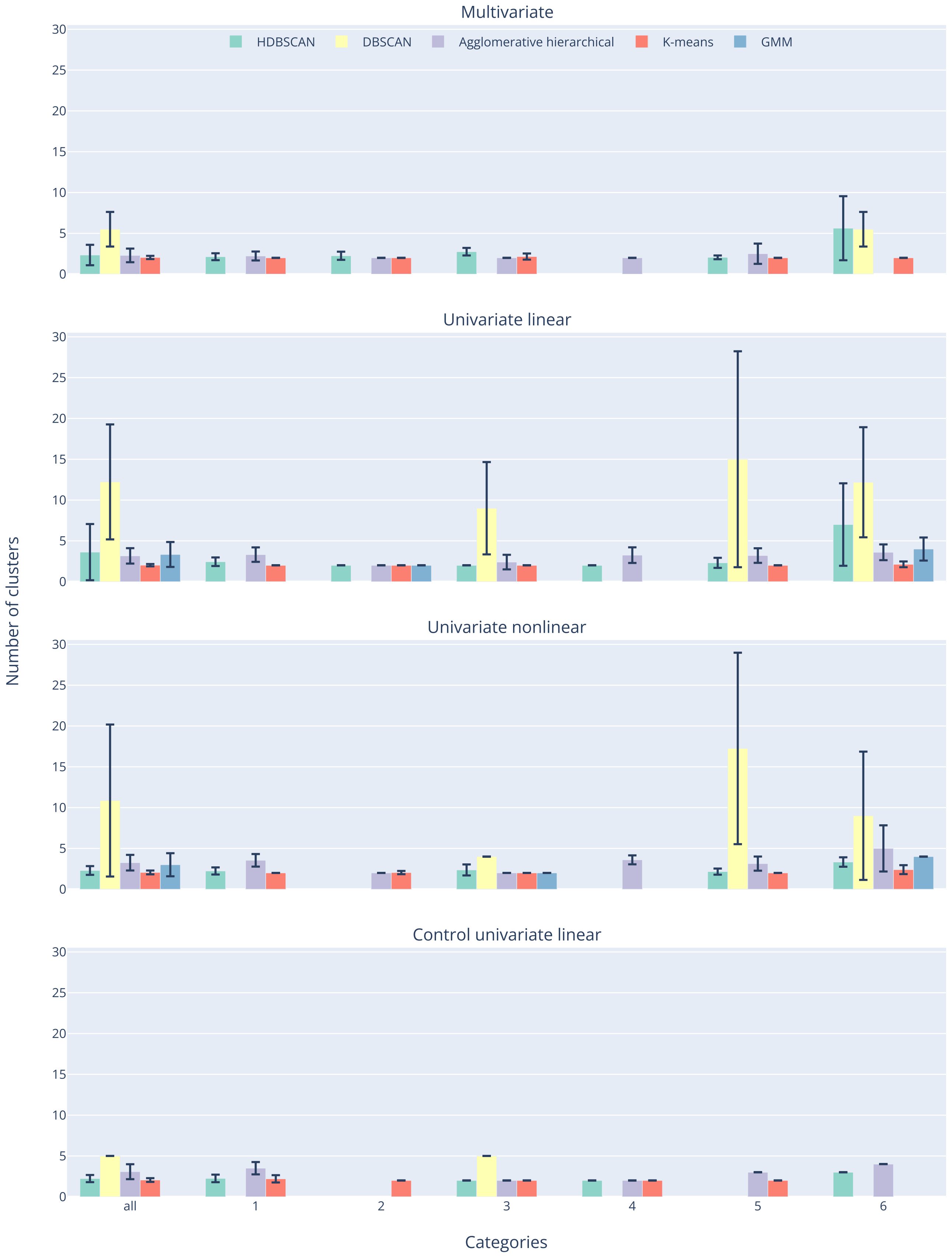

Fig. S15. Mean and standard deviation of the number of clusters in the clustering solutions analysed for each feature group, each clustering method, and for all categories and each category.

6.5 Preictal comparison between EEG and ECG

Fig. S16 presents information regarding the existence of a putative preictal state in EEG (reported in the present study) and ECG (reported in[18]) data. Additionally, the identified preictal intervals were also characterised in terms of starting time before seizure onset for each modality (see Fig. S17).

The preictal intervals identified in the ECG data were selected according to the time continuity and duration (as reported in Leal *et al.*[18]).

In the case of EEG, the preictal intervals represented in this section were found for either category 3 or 6. When preictal patterns were observed for more than one group of features a final interval was chosen according to the density, duration and starting time before seizure onset (as explained in section 2).

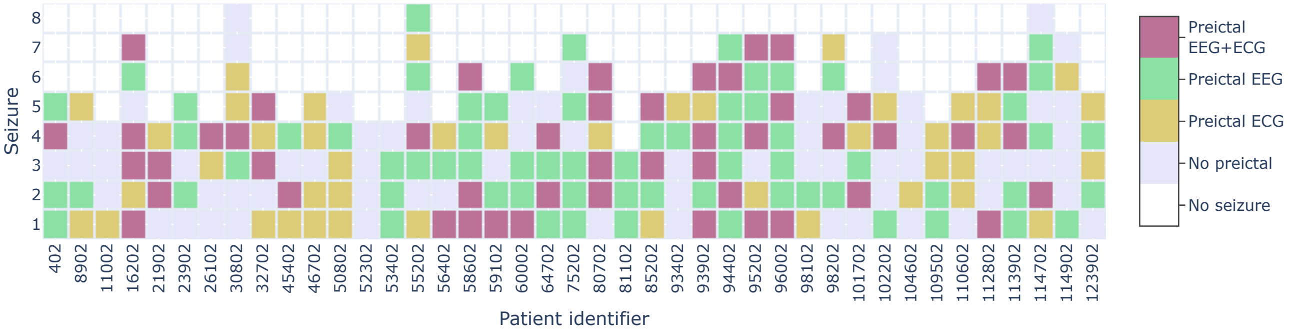

Fig. S16. Results for preictal interval identification in the EEG lead seizures. The preictal interval was found for (i) 37 patients (90%) and 116 seizures (51%) in EEG data and (ii) 36 patients (88%) and 92 seizures (41%) in ECG data. Preictal patterns were found in both EEG and ECG in 22% of the analysed lead seizures (seizures separated from 4.5 hours from the preceding seizure).

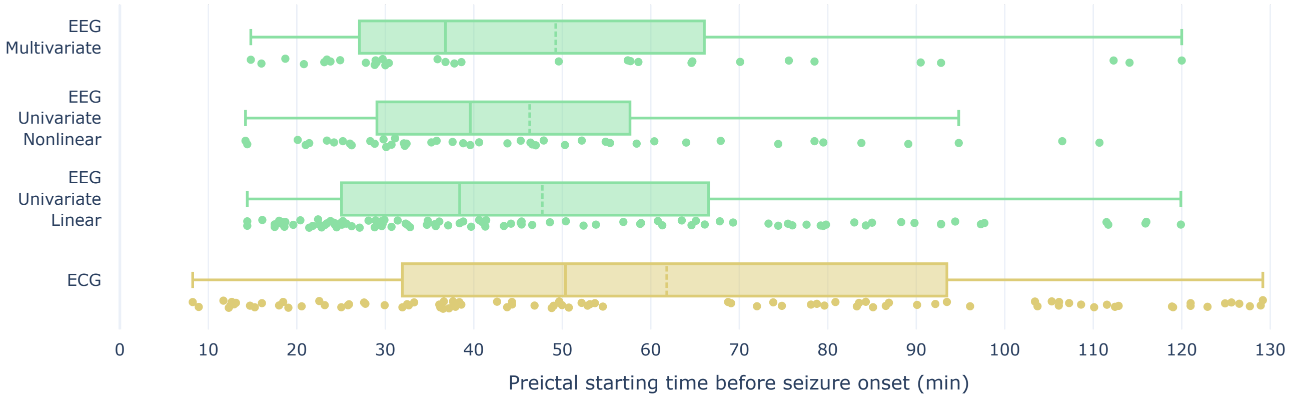

Fig. S17. Results for preictal interval characterisation in terms of starting time before seizure EEG onset, when preictal patterns were found in the analysed EEG lead seizures. Dots correspond to the values of preictal starting time. Solid and dashed lines indicate medians and means, respectively. Box's tops and bottoms indicate the 75th and 25th percentiles, respectively. Whiskers refer to the span of preictal starting time after discarding outliers.

6.6 State-of-the-art preictal comparison

Fig. S18 and Table S9 present the comparison between the preictal intervals obtained using unsupervised learning and the preictal intervals obtained using grid-search supervised learning. This comparison was made for two studies[5,6] reporting preictal grid-search during seizure prediction model training using the EPILEPSIAE database. The authors provided identification numbers for each patient, allowing for a patient-wise comparison of preictal starting time. In Pinto *et al.* 2021 study[5], the authors present the training results for different values of SOP in their supplementary material. The values we used to perform the study comparison correspond to the average preictal (SOP plus 10 minutes SPH) interval with the highest value of training fitness. Additionally, when the fitness values were equal for different average preictal intervals, we chose the average preictal interval that starts closer to the seizure onset, as it means that the patient has to wait less time for a seizure to occur.

The average preictal interval found using unsupervised learning was obtained for each patient by averaging over the starting time of the identified preictal intervals (which were often not found for all seizures of a patient). The criteria to get a final preictal interval when preictal intervals were found for more than one group of features was clarified in section 2.

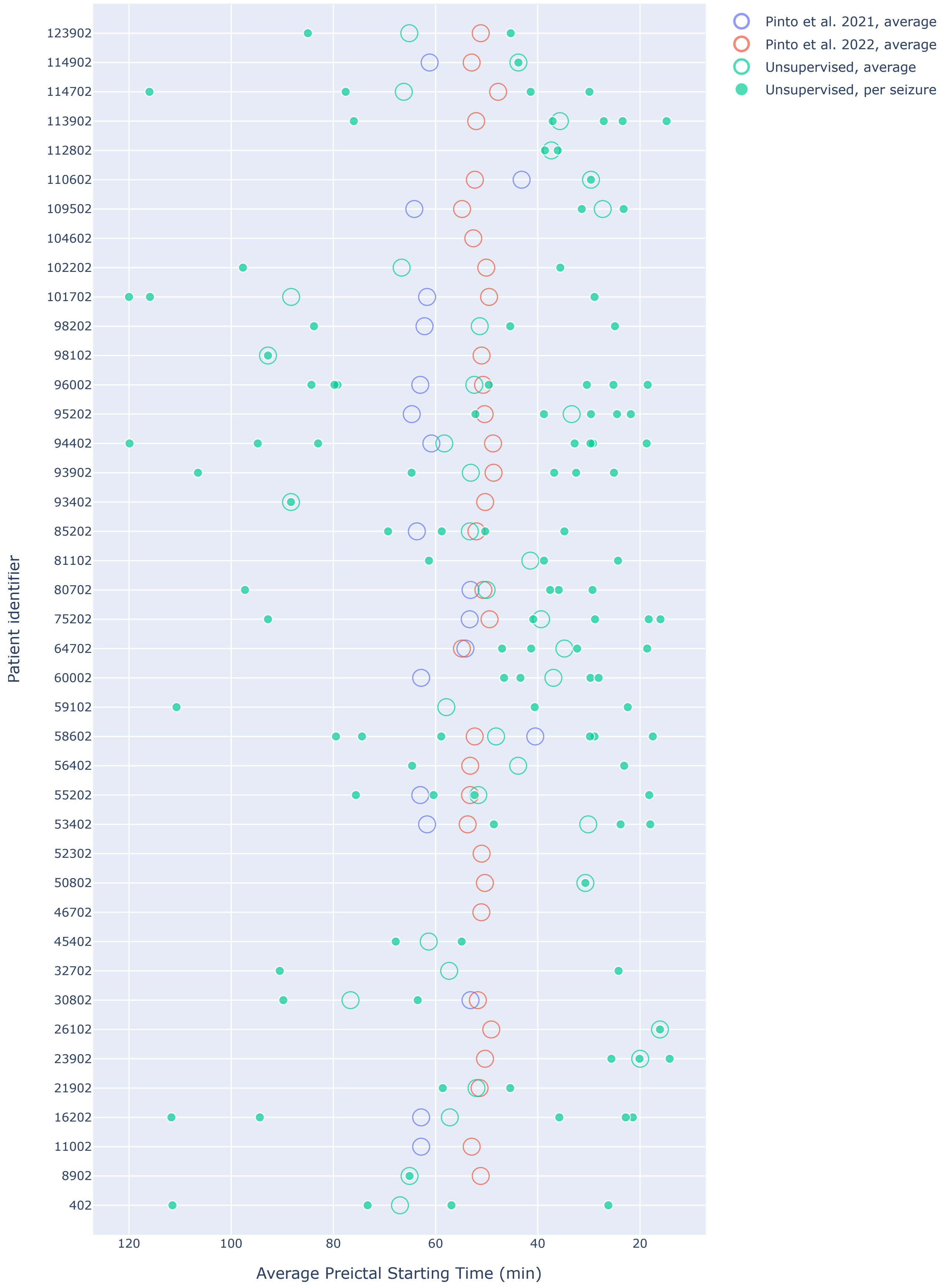

Fig. S18. Representation of the preictal starting time before onset found using unsupervised learning (classes 3 and 6) and using preital grid-search in the two studies by Pinto *el al.*[5,6].

Table S9: Comparison of the preictal starting time before seizure onset found in the unsupervised learning study with the preictal intervals obtained using grid-search supervised learning in two studies[5,6].

Patient index	Patient identifier	Pinto *et al.* 2021[5]		Pinto *et al.* 2022[6]		Unsupervised preictal learning			
		Mean	SD	Mean	SD	Mean	SD	#LSz	#PLSz
1	402					56.63	21.49	5	4
2	8902			51.17	9.08	65.10		5	1
3	11 002	62.83	3.34	52.94	10.65			4	0
4	16 202	62.83	3.58			39.02	31.56	7	5
5	21 902			51.40	9.55	54.70	13.15	4	2
6	23 902			50.33	10.39	19.97	5.70	5	3
7	26 102			49.11	7.34	16.10		4	1
8	30 802	53.17	3.02	51.73	8.31	76.65	18.60	8	2
9	32 702					19.30	6.93	5	2
10	45 402					61.35	9.12	4	2
11	46 702			51.05	8.08			5	0
12	50 802			50.36	7.44	30.70		5	1
13	52 302			51.00	8.17			4	0
14	53 402	61.67	2.69	53.73	7.61	26.53	10.18	4	3
15	55 202	63.00	3.56	53.26	9.34	51.63	24.25	8	4
16	56 402			53.23	6.88	45.30	27.29	4	2
17	58 602	40.50	1.50	52.35	11.14	48.17	26.21	6	6
18	59 102					57.90	46.62	5	3
19	60 002	62.83	2.79			36.95	9.41	6	4
20	64 702	54.17	3.67	54.85	7.57	27.43	11.44	5	4
21	75 202	53.33	2.98	49.45	8.64	45.20	33.05	7	4
22	80 702	53.17	2.41	50.65	8.26	48.13	33.05	6	4
23	81 102					41.47	18.64	3	3
24	85 202	63.67	2.56	52.02	11.45	51.78	15.30	5	4
25	93 402			50.31	8.29	88.30		5	1
26	93 902			48.66	7.89	49.70	38.09	6	4
27	94 402	60.83	1.86	48.76	9.00	57.90	40.35	7	7
28	95 202	64.67	2.87	50.41	10.48	32.94	12.48	7	5
29	96 002	63.00	3.06	50.72	7.60	46.71	32.27	7	7
30	98 102			51.01	8.16	92.80		5	1
31	98 202	62.17	2.79			41.37	14.87	7	3
32	101 702	61.67	2.69	49.54	8.12	72.40	61.52	5	2
33	102 202			50.09	8.40	66.65	43.91	7	2
34	104 602			52.63	8.68			5	0
35	109 502	64.17	3.44	54.82	8.58	27.30	5.80	4	2
36	110 602	43.17	3.98	52.32	8.01	29.60		5	1
37	112 802					36.10		6	1
38	113 902			52.06	7.22	35.68	23.92	6	5
39	114 702			47.77	7.98	66.23	38.91	8	4
40	114 902	61.17	2.48	52.95	8.07	43.80		7	1
41	123 902			51.17	9.63	65.15	28.07	5	2
Mean		58.53		51.31		50.06			
SD		7.08		1.73		28.92			

SD: standard deviation. Empty cells indicate that patients were not analysed in Pinto *et al.* studies[5,6]. Empty cells found in unsupervised preictal learning column correspond to patients for which no preictal interval was identified. #LSz: number of lead seizures analysed in study. #PLSz: number of lead seizures with unsupervised preictal interval.

7 Metadata analysis

The metadata analysis was performed for each feature group to infer about the influence of seizure-characterising variables on the preictal identification results. As shown in Table S2, vigilance state, onset hour, and ILAE seizure classification are categorical variables, whereas the noise variable is a continuous numerical variable.

First, we computed the Kruskal-Wallis statistical test between the pairs of categorical and numerical variables indicated in Table S10. The returned p-values indicated that the Kruskal-Wallis test rejected the null hypothesis that the pairs of variables came from the same distribution at a 1% significance level.

The bias-corrected Cramér's V measure was computed to verify the association between each pair of categorical variables. Cramér's V values vary from 0 (corresponding to no association between the variables) to 1 (complete association). The Cramér's V measure corresponds to the absolute value of the phi coefficient when the two variables under study are binary variables. The results in Table S10 show that no pair of categorical variables verifies any association.

Table S10: Metadata analysis for the output of the preictal study for each seizure.

		**Preictal**	**Multivariate**	**Univariate linear**	**Univariate nonlinear**
		0: No preictal; 1: Preictal	0: No preictal; 1: Category 3 preictal; 2: Category 6 preictal		
**ILAE classification**	0: FOIA; 1: FOA; 2: FBTC; 3: UC	0.14†	0.09†	0.03†	0.00†
**Vigilance state**	0: W; 1: N1; 2: N2; 3: R	0.00†	0.00†	0.00†	0.06†
**Onset hour**	24 categories	0.38†	0.20†	0.26†	0.22†
**Noise**		1.21e-56*	1.09e-72*	2.05e-51*	5.44e-68*

Seizure vigilance state: wakefulness (W), NREM sleep stage I (N1), NREM sleep stage II (N2), REM sleep stage (R). Seizure ILAE classification: focal onset aware (FOA), focal onset impaired awareness (FOIA), focal to bilateral tonic-clonic (FBTC), unclassified (UC). *Cramér's V association measure. †p-value of the Kruskal-Wallis statistical test.

A second analysis (see Table S11) was performed to evaluate the possible association between the continuous preictal characteristics (duration, density, and starting time) and the categorical (vigilance state, ILAE classification, and onset hour) and continuous (percentage of noise) metadata variables.

The results for the Kruskal-Wallis statistical test between the pairs of categorical and continuous variables indicate that the null hypothesis that the pairs of variables came from the same distribution was rejected for all but one pair of variables, at a 5% significance level. Even though the null hypothesis has not been rejected when Kruskal-Wallis statistical test was applied to the pair onset hour and multivariate preictal duration, it is important to note that this group contains 33 samples (preictal was found for the data of 33 seizures) and 16 categories (16 discrete hours from the 24 hour discretisation period where seizure onset occurred).

Finally, we computed the Pearson's correlation coefficient between the continuous variables: the percentage of noise and each of the preictal characteristics (starting time, duration, and density) found for the seizures assigned categories 3 and 6 (see Table S11). The results indicate that no correlation was found between the obtained preictal characteristics and the percentage of noise in the EEG signals.

Table S11: Metadata analysis for the seizures for which a preictal interval has been observed (categories 3 and 6).

Metadata variable	Feature group	Preictal duration	Preictal density	Preictal starting time
**Vigilance state**	Multivariate (W and N2)	2.80e-13†	2.84e-13†	2.88e-13†
	Univariate linear (all categories)	7.31e-37†	9.91e-37†	7.35e-37†
	Univariate nonlinear (W, N1, and N2)	7.80e-18†	8.08e-18†	7.81e-18†
**ILAE classification**	Multivariate (all categories)	1.92e-12†	3.03e-12†	1.97e-12†
	Univariate linear (all categories)	0.29e-34†	1.23e-34†	0.29e-34†
	Univariate nonlinear (all categories)	3.47e-17†	4.33e-17†	3.47e-17†
**Onset hour**	Multivariate (16 categories)	**0.14**†	0.34e-11†	4.95e-11†
	Univariate linear (all categories)	0.02†	2.33e-29†	6.00e-29†
	Univariate nonlinear (18 categories)	3.89e-05†	2.15e-14†	0.18e-14†
**Noise**	Multivariate	4.51‡	2.88‡	15.05‡
	Univariate linear	11.27‡	9.00‡	22.94‡
	Univariate nonlinear	9.46‡	23.74‡	0.97‡

Seizure vigilance state: wakefulness (W), NREM sleep stage I (N1), NREM sleep stage II (N2), REM sleep stage (R). Seizure ILAE classification: focal onset aware (FOA), focal onset impaired awareness (FOIA), focal to bilateral tonic-clonic (FBTC), unclassified (UC). †*p*-value of the Kruskal-Wallis statistical test (values above the 5% level of significance are in bold). ‡ Pearson's correlation coefficient. The preictal was found for 33, 101, and 47 seizures for the multivariate, univariate linear, and univariate nonlinear feature groups.

References

1. Pottkämper JCM, Hofmeijer J, van Waarde JA, van Putten MJAM. The postictal state — What do we know? Epilepsia. 2020 6;61(6):1045-61. Available from: https://doi.org/10.1111/epi.16519.
2. Payne DE, Karoly PJ, Freestone DR, Boston R, D'Souza W, Nurse E, et al. Postictal suppression and seizure durations: A patient-specific, long-term iEEG analysis. Epilepsia. 2018 5;59(5):1027-36. Available from: https://doi.org/10.1111/epi.14065.
3. So NK, Blume WT. The postictal EEG. Epilepsy & Behavior. 2010 10;19(2):121-6. Available from: https://doi.org/10.1016/j.yebeh.2010.06.033.
4. Meisel C, Bailey KA. Identifying signal-dependent information about the preictal state: A comparison across ECoG, EEG and EKG using deep learning. EBioMedicine. 2019 7;45:422-31. Available from: https://doi.org/10.1016/j.ebiom.2019.07.001.
5. Pinto MF, Leal A, Lopes F, Dourado A, Martins P, Teixeira CA. A personalized and evolutionary algorithm for interpretable EEG epilepsy seizure prediction. Scientific Reports. 2021 12;11(1):3415. Available from: https://doi.org/10.1038/s41598-021-82828-7.
6. Pinto M, Coelho T, Leal A, Lopes F, Dourado A, Martins P, et al. Interpretable EEG seizure prediction using a multiobjective evolutionary algorithm. Scientific Reports. 2022 12;12(1):4420. Available from: https://doi.org/10.1038/s41598-022-08322-w.
7. Wolf P, Lin K, Nikanorova M. Non-Pharmacological Therapy of Epilepsy. In: Shorvon S, Guerrini R, Cook M, Lhatoo S, editors. Oxford Textbook of Epilepsy and Epileptic Seizures. Oxford University Press; 2012. p. 135-43. Available from: http://oxfordmedicine.com/view/10.1093/med/9780199659043.001.0001/med-9780199659043-chapter-012.
8. Boddu SHS, Kumari S. A Short Review on the Intranasal Delivery of Diazepam for Treating Acute Repetitive Seizures. Pharmaceutics. 2020 11;12(12):1167.
9. Bouw MR, Chung SS, Gidal B, King A, Tomasovic J, Wheless JW, et al. Clinical pharmacokinetic and pharmacodynamic profile of midazolam nasal spray. Epilepsy Research. 2021 3;171:106567. Available from: https://doi.org/10.1016/j.eplepsyres.2021.106567.
10. Schelter B, Winterhalder M, Drentrup HFg, Wohlmuth J, Nawrath J, Brandt A, et al. Seizure prediction: The impact of long prediction horizons. Epilepsy Research. 2007 2;73(2):213-7. Available from: https://doi.org/10.1016/j.eplepsyres.2006.10.002.
11. Winterhalder M, Maiwald T, Voss HU, Aschenbrenner-Scheibe R, Timmer J, Schulze-Bonhage A. The seizure prediction characteristic: a general framework to assess and compare seizure prediction methods. Epilepsy & Behavior. 2003 6;4(3):318-25. Available from: https://doi.org/10.1016/S1525-5050(03)00105-7.
12. Klatt J, Feldwisch-Drentrup H, Ihle M, Navarro V, Neufang M, Teixeira C, et al. The EPILEPSIAE database: An extensive electroencephalography database of epilepsy patients. Epilepsia. 2012 9;53(9):1669-76. Available from: https://doi.org/10.1111/j.1528-1167.2012.03564.x.
13. Berg AT, Berkovic SF, Brodie MJ, Buchhalter J, Cross JH, van Emde Boas W, et al. Revised terminology and concepts for organization of seizures and epilepsies: Report of the ILAE Commission on Classification and Terminology, 2005-2009. Epilepsia. 2010 4;51(4):676-85. Available from: https://doi.org/10.1111/j.1528-1167.2010.02522.x.
14. Scheffer IE, Berkovic S, Capovilla G, Connolly MB, French J, Guilhoto L, et al. ILAE classification of the epilepsies: Position paper of the ILAE Commission for Classification and Terminology. Epilepsia. 2017 4;58(4):512-21. Available from: http://doi.wiley.com/10.1111/epi.13709.
15. Ihle M, Feldwisch-Drentrup H, Teixeira CA, Witon A, Schelter B, Timmer J, et al. EPILEPSIAE – A European epilepsy database. Computer Methods and Programs in Biomedicine. 2012 6;106(3):127-38. Available from: https://doi.org/10.1016/j.cmpb.2010.08.011.
16. Fisher RS, Cross JH, French JA, Higurashi N, Hirsch E, Jansen FE, et al. Operational classification of seizure types by the International League Against Epilepsy: Position Paper of the ILAE Commission for Classification and Terminology. Epilepsia. 2017 4;58(4):522-30. Available from: https://doi.org/10.1111/epi.13670.
17. Karoly PJ, Rao VR, Gregg NM, Worrell GA, Bernard C, Cook MJ, et al. Cycles in epilepsy. Nature Reviews Neurology. 2021 5;17(5):267-84. Available from: https://www.nature.com/articles/s41582-021-00464-1.
18. Leal A, Pinto MF, Lopes F, Bianchi AM, Henriques J, Ruano MG, et al. Heart rate variability analysis for the identification of the preictal interval in patients with drug-resistant epilepsy. Scientific Reports. 2021 12;11(1):5987. Available from: https://doi.org/10.1038/s41598-021-85350-y.

19. Bou Assi E, Nguyen DK, Rihana S, Sawan M. Towards accurate prediction of epileptic seizures: A review. Biomedical Signal Processing and Control. 2017 4;34:144-57. Available from: https://doi.org/10.1016/j.bspc.2017.02.001.

20. Mormann F, Andrzejak RG, Elger CE, Lehnertz K. Seizure prediction: the long and winding road. Brain. 2007 2;130(2):314-33. Available from: https://doi.org/10.1093/brain/awl241.

21. Valderrama M, Alvarado C, Nikolopoulos S, Martinerie J, Adam C, Navarro V, et al. Identifying an increased risk of epileptic seizures using a multi-feature EEG–ECG classification. Biomedical Signal Processing and Control. 2012 5;7(3):237-44. Available from: https://doi.org/10.1016/j.bspc.2011.05.005.

22. Wennberg R. Introduction to EEG for Nonepileptologists Working in Seizure Prediction and Dynamics. In: Osorio I, Zaveri HP, Frei MG, Arthurs S, editors. Epilepsy: The Intersection of Neurosciences, Biology, Mathematics, Engineering, and Physics. 1st ed. Boca Raton, FL: CRC Press; 2011. p. 23-39. Available from: https://doi.org/10.1201/b10866.

23. Nunez PL, Srinivasan R. Fallacies in EEG. In: Nunez PL, Srinivasan R, editors. Electric Fields of the Brain. 2nd ed. Oxford University Press; 2006. p. 56-98. Available from: https://doi.org/10.1093/acprof:oso/9780195050387.003.0002.

24. Mormann F, Kreuz T, Rieke C, Andrzejak RG, Kraskov A, David P, et al. On the predictability of epileptic seizures. Clinical Neurophysiology. 2005 3;116(3):569-87. Available from: https://doi.org/10.1016/j.clinph.2004.08.025.

25. Teixeira CA, Direito B, Bandarabadi M, Le Van Quyen M, Valderrama M, Schelter B, et al. Epileptic seizure predictors based on computational intelligence techniques: A comparative study with 278 patients. Computer Methods and Programs in Biomedicine. 2014 5;114(3):324-36. Available from: https://doi.org/10.1016/j.cmpb.2014.02.007.

26. Direito B, Teixeira CA, Sales F, Castelo-Branco M, Dourado A. A Realistic Seizure Prediction Study Based on Multiclass SVM. International Journal of Neural Systems. 2017 5;27(03):1-15. Available from: https://doi.org/10.1142/S012906571750006X.

27. Nasehi S, Pourghassem H. Seizure Detection Algorithms Based on Analysis of EEG and ECG Signals: a Survey. Neurophysiology. 2012 6;44(2):174-86. Available from: http://link.springer.com/10.1007/s11062-012-9285-x.

28. Rasekhi J, Mollaei MRK, Bandarabadi M, Teixeira CA, Dourado A. Preprocessing effects of 22 linear univariate features on the performance of seizure prediction methods. Journal of Neuroscience Methods. 2013 7;217(1-2):9-16. Available from: https://doi.org/10.1016/j.jneumeth.2013.03.019.

29. Sheng Y. Wavelet Transform. In: Poularikas AD, editor. Transforms and Applications Handbook. 3rd ed. CRC Press; 2010. p. 1-10.

30. Faust O, Acharya UR, Adeli H, Adeli A. Wavelet-based EEG processing for computer-aided seizure detection and epilepsy diagnosis. Seizure. 2015;26:56-64. Available from: http://www.sciencedirect.com/science/article/pii/S1059131115000138.

31. Acharya UR, Vinitha Sree S, Swapna G, Martis RJ, Suri JS. Automated EEG analysis of epilepsy: A review. Knowledge-Based Systems. 2013 6;45:147-65. Available from: https://doi.org/10.1016/j.knosys.2013.02.014.

32. Rasekhi J, Mollaei MK, Bandarabadi M, Teixeira C, Dourado A. Epileptic seizure prediction based on ratio and differential linear univariate features. Journal of Medical Signals & Sensors. 2015 1;5(1):1. Available from: http://www.jmssjournal.net/text.asp?2015/5/1/1/150371.

33. Ruiz-Padial E, Ibáñez-Molina AJ. Fractal dimension of EEG signals and heart dynamics in discrete emotional states. Biological Psychology. 2018 9;137:42-8. Available from: https://doi.org/10.1016/j.biopsycho.2018.06.008.

34. Kawe TNJ, Shadli SM, McNaughton N. Higuchi's fractal dimension, but not frontal or posterior alpha asymmetry, predicts PID-5 anxiousness more than depressivity. Scientific Reports. 2019 12;9(1):19666. Available from: https://doi.org/10.1038/s41598-019-56229-w.

35. Kesić S, Spasić SZ. Application of Higuchi's fractal dimension from basic to clinical neurophysiology: A review. Computer Methods and Programs in Biomedicine. 2016 9;133:55-70. Available from: https://doi.org/10.1016/j.cmpb.2016.05.014.

36. Ihlen EAF. Introduction to Multifractal Detrended Fluctuation Analysis in Matlab. Frontiers in Physiology. 2012 6;3:141. Available from: https://doi.org/10.3389/fphys.2012.00141.

37. Bryce RM, Sprague KB. Revisiting detrended fluctuation analysis. Scientific Reports. 2012 12;2(1):315. Available from: https://doi.org/10.1038/srep00315.

38. Morales Martínez JL, Segovia-Domínguez I, Rodríguez IQ, Horta-Rangel FA, Sosa-Gómez G. A modified Multifractal Detrended Fluctuation Analysis (MFDFA) approach for multifractal analysis of precipitation. Physica A: Statistical Mechanics and its Applications. 2021 3;565:125611. Available from: https://doi.org/10.1016/j.physa.2020.125611.

39. Tang L, Xie N, Zhao M, Wu X. Seizure Prediction Using Multi-View Features and Improved Convolutional Gated Recurrent Network. IEEE Access. 2020 9;8:172352-61. Available from: https://ieeexplore.ieee.org/document/9200354/.

40. Sikdar D, Roy R, Mahadevappa M. Epilepsy and seizure characterisation by multifractal analysis of EEG subbands. Biomedical Signal Processing and Control. 2018 3;41:264-70. Available from: https://linkinghub.elsevier.com/retrieve/pii/S1746809417302896.

41. Serrano E, Figliola A. Wavelet Leaders: A new method to estimate the multifractal singularity spectra. Physica A: Statistical Mechanics and its Applications. 2009 7;388(14):2793-805. Available from: https://doi.org/10.1016/j.physa.2009.03.043.

42. França LGS, Montoya P, Miranda JGV. On multifractals: a non-linear study of actigraphy data. Physica A: Statistical Mechanics and its Applications. 2017 2;514:612-9. Available from: http://arxiv.org/abs/1702.03912http://dx.doi.org/10.1016/j.physa.2018.09.122.

43. Bose R, Pratiher S, Chatterjee S. Detection of epileptic seizure employing a novel set of features extracted from multifractal spectrum of electroencephalogram signals. IET Signal Processing. 2019 4;13(2):157-64. Available from: https://onlinelibrary.wiley.com/doi/10.1049/iet-spr.2018.5258.

44. Freitas Cruz I, Sampaio J. Multifractal Analysis of Movement Behavior in Association Football. Symmetry. 2020 8;12(8):1287. Available from: https://www.mdpi.com/2073-8994/12/8/1287/htmhttps://www.mdpi.com/2073-8994/12/8/1287.

45. Acharya UR, Molinari F, Sree SV, Chattopadhyay S, Ng KH, Suri JS. Automated diagnosis of epileptic EEG using entropies. Biomedical Signal Processing and Control. 2012 7;7(4):401-8. Available from: https://doi.org/10.1016/j.bspc.2011.07.007.

46. Acharya UR, Fujita H, Sudarshan VK, Bhat S, Koh JEW. Application of entropies for automated diagnosis of epilepsy using EEG signals: A review. Knowledge-Based Systems. 2015 11;88:85-96. Available from: https://doi.org/10.1016/j.knosys.2015.08.004.

47. Acharya UR, Hagiwara Y, Adeli H. Automated seizure prediction. Epilepsy & Behavior. 2018 11;88:251-61. Available from: https://doi.org/10.1016/j.yebeh.2018.09.030.

48. Pincus SM. Approximate entropy as a measure of system complexity. Proceedings of the National Academy of Sciences. 1991 3;88(6):2297-301. Available from: https://doi.org/10.1073/pnas.88.6.2297.

49. Richman JS, Moorman JR. Physiological time-series analysis using approximate entropy and sample entropy. American Journal of Physiology-Heart and Circulatory Physiology. 2000 6;278(6):H2039-49. Available from: https://doi.org/10.1152/ajpheart.2000.278.6.H2039.

50. Delgado-Bonal A, Marshak A. Approximate Entropy and Sample Entropy: A Comprehensive Tutorial. Entropy. 2019 5;21(6):1-37. Available from: https://doi.org/10.3390/e21060541.

51. Varsavsky A, Mareels I, Cook M. Epileptic seizure and the EEG: Measurement, Models, Detection and Prediction. Boca Raton, FL: CRC Press; 2011. Available from: https://books.google.pt/books?id=fw7LBQAAQBAJ.

52. Lekscha J, Donner RV. Phase space reconstruction for non-uniformly sampled noisy time series. Chaos: An Interdisciplinary Journal of Nonlinear Science. 2018 8;28(8):1-12. Available from: https://doi.org/10.1063/1.5023860.

53. Kennel MB, Brown R, Abarbanel HDI. Determining embedding dimension for phase-space reconstruction using a geometrical construction. Physical Review A. 1992 3;45(6):3403-11. Available from: https://doi.org/10.1103/PhysRevA.45.3403.

54. Fraser AM, Swinney HL. Independent coordinates for strange attractors from mutual information. Physical Review A. 1986 2;33(2):1134-40. Available from: https://doi.org/10.1103/PhysRevA.33.1134.

55. Rosenstein MT, Collins JJ, De Luca CJ. A practical method for calculating largest Lyapunov exponents from small data sets. Physica D: Nonlinear Phenomena. 1993 5;65(1-2):117-34. Available from: https://doi.org/10.1016/0167-2789(93)90009-P.

56. Grassberger P, Procaccia I. Characterization of Strange Attractors. Physical Review Letters. 1983 1;50(5):346-9. Available from: https://doi.org/10.1103/PhysRevLett.50.346.

57. Eckmann JP, Kamphorst SO, Ruelle D. Recurrence Plots of Dynamical Systems. Europhysics Letters (EPL). 1987 11;4(9):973-7. Available from: https://doi.org/10.1209/0295-5075/4/9/004.

58. MARWAN N, CARMENROMANO M, THIEL M, KURTHS J. Recurrence plots for the analysis of complex systems. Physics Reports. 2007 1;438(5-6):237-329. Available from: https://doi.org/10.1016/j.physrep.2006.11.001.

59. Marwan N, Webber CL. Mathematical and Computational Foundations of Recurrence Quantifications. In: Webber JC, Marwan N, editors. Recurrence Quantification Analysis. Understanding Complex Systems. Springer, Cham; 2015. p. 3-43. Available from: https://doi.org/10.1007/978-3-319-07155-8_1.

60. Shahsavari Baboukani P, Azemi G, Boashash B, Colditz P, Omidvarnia A. A novel multivariate phase synchrony measure: Application to multichannel newborn EEG analysis. Digital Signal Processing. 2019 1;84:59-68. Available from: https://doi.org/10.1016/j.dsp.2018.08.019.

61. Niso G, Bruña R, Pereda E, Gutiérrez R, Bajo R, Maestú F, et al. HERMES: Towards an Integrated Toolbox to Characterize Functional and Effective Brain Connectivity. Neuroinformatics. 2013 10;11(4):405-34. Available from: https://doi.org/10.1007/s12021-013-9186-1.

62. Cohen MX. Analyzing neural time series data: Theory and practice. No. 1 in Issues in clinical and cognitive neuropsychology. MIT Press; 2014. Available from: https://books.google.pt/books?id=rDKkAgAAQBAJ.

63. Kida T, Tanaka E, Kakigi R. Multi-Dimensional Dynamics of Human Electromagnetic Brain Activity. Frontiers in Human Neuroscience. 2016 1;9(JAN2016):713. Available from: https://doi.org/10.3389/fnhum.2015.00713.

64. Anastasiadou MN, Christodoulakis M, Papathanasiou ES, Papacostas SS, Hadjipapas A, Mitsis GD. Graph Theoretical Characteristics of EEG-Based Functional Brain Networks in Patients With Epilepsy: The Effect of Reference Choice and Volume Conduction. Frontiers in Neuroscience. 2019 3;13:221. Available from: https://doi.org/10.3389/fnins.2019.00221.

65. Mormann F, Andrzejak RG, Kreuz T, Rieke C, David P, Elger CE, et al. Automated detection of a preseizure state based on a decrease in synchronization in intracranial electroencephalogram recordings from epilepsy patients. Physical Review E. 2003 2;67(2):021912. Available from: https://doi.org/10.1103/PhysRevE.67.021912.

66. Mirowski P, Madhavan D, LeCun Y, Kuzniecky R. Classification of patterns of EEG synchronization for seizure prediction. Clinical Neurophysiology. 2009 11;120(11):1927-40. Available from: https://doi.org/10.1016/j.clinph.2009.09.002.

67. Rubinov M, Sporns O. Complex network measures of brain connectivity: Uses and interpretations. NeuroImage. 2010 9;52(3):1059-69. Available from: https://doi.org/10.1016/j.neuroimage.2009.10.003.

68. Lehnertz K, Geier C, Rings T, Stahn K. Capturing time-varying brain dynamics. EPJ Nonlinear Biomedical Physics. 2017 6;5:2. Available from: https://doi.org/10.1051/epjnbp/2017001.

69. Park Y, Luo L, Parhi KK, Netoff T. Seizure prediction with spectral power of EEG using cost-sensitive support vector machines. Epilepsia. 2011;52(10):1761-70. Available from: https://doi.org/10.1111/j.1528-1167.2011.03138.x.

70. Teixeira CA, Direito B, Feldwisch-Drentrup H, Valderrama M, Costa RP, Alvarado-Rojas C, et al. EPILAB: A software package for studies on the prediction of epileptic seizures. Journal of Neuroscience Methods. 2011 9;200(2):257-71. Available from: https://doi.org/10.1016/j.jneumeth.2011.07.002.

71. Ramgopal S, Thome-Souza S, Jackson M, Kadish NE, Sánchez Fernández I, Klehm J, et al. Seizure detection, seizure prediction, and closed-loop warning systems in epilepsy. Epilepsy & Behavior. 2014 8;37:291-307. Available from: https://doi.org/10.1016/j.yebeh.2014.06.023.

72. Henriques T, Ribeiro M, Teixeira A, Castro L, Antunes L, Costa-Santos C. Nonlinear Methods Most Applied to Heart-Rate Time Series: A Review. Entropy. 2020 3;22(3):1-39. Available from: https://www.mdpi.com/1099-4300/22/3/309.

73. Malekzadeh A, Zare A, Yaghoobi M, Alizadehsani R. Automatic Diagnosis of Epileptic Seizures in EEG Signals Using Fractal Dimension Features and Convolutional Autoencoder Method. Big Data and Cognitive Computing. 2021 12;5(4):78. Available from: https://www.mdpi.com/2504-2289/5/4/78/htmhttps://www.mdpi.com/2504-2289/5/4/78.

74. Malekzadeh A, Zare A, Yaghoobi M, Kobravi HR, Alizadehsani R. Epileptic Seizures Detection in EEG Signals Using Fusion Handcrafted and Deep Learning Features. Sensors. 2021 11;21(22):7710. Available from: https://www.mdpi.com/1424-8220/21/22/7710/htmhttps://www.mdpi.com/1424-8220/21/22/7710.

75. Auno S, Lauronen L, Wilenius J, Peltola M, Vanhatalo S, Palva JM. Detrended fluctuation analysis in the presurgical evaluation of parietal lobe epilepsy patients. Clinical Neurophysiology. 2021 7;132(7):1515-25. Available from: https://linkinghub.elsevier.com/retrieve/pii/S1388245721005319.

76. Frolov NS, Grubov VV, Maksimenko VA, Lüttjohann A, Makarov VV, Pavlov AN, et al. Statistical Properties and Predictability of Extreme Epileptic Events. Scientific Reports. 2019 12;9(1):7243. Available from: https://www.nature.com/articles/s41598-019-43619-3http://www.nature.com/articles/s41598-019-43619-3.

77. Gadhoumi K, Lina JM, Mormann F, Gotman J. Seizure prediction for therapeutic devices: A review. Journal of Neuroscience Methods. 2016;260(029):270-82. Available from: https://doi.org/10.1016/j.jneumeth.2015.06.010.

78. Bandarabadi M, Teixeira CA, Rasekhi J, Dourado A. Epileptic seizure prediction using relative spectral power features. Clinical Neurophysiology. 2015 2;126(2):237-48. Available from: https://doi.org/10.1016/j.clinph.2014.05.022.

79. Ester M. Density-Based Clustering. In: Aggarwal CC, Reddy CK, editors. Data Clustering: Algorithms and Applications. 1st ed. Chapman & Hall/CRC; 2014. p. 111-26. Available from: https://www.crcpress.com/Data-Clustering-Algorithms-and-Applications/Aggarwal-Reddy/p/book/9781466558212.

80. Tan PN, Steinbach M, Karpatne A, Kumar V. Cluster Analysis: Basic Concepts and Algorithms. In: Introduction to Data Mining. 2nd ed. Pearson Education; 2019. p. 866.

81. Wierzchoń ST, Kłopotek MA. Algorithms of Combinatorial Cluster Analysis. In: Modern Algorithms of Cluster Analysis. Studies in Big Data. vol. 34. Springer, Cham; 2018. p. 67-161. Available from: http://link.springer.com/10.1007/978-3-319-69308-8_3.

82. Ferreira L, Hitchcock DB. A Comparison of Hierarchical Methods for Clustering Functional Data. Communications in Statistics - Simulation and Computation. 2009 10;38(9):1925-49. Available from: http://www.tandfonline.com/doi/abs/10.1080/03610910903168603.

83. Ester M, Kriegel HP, Sander J, Xu X. A Density-Based Algorithm for Discovering Clusters in Large Spatial Databases with Noise. In: Proceedings of the 2nd International Conference on Knowledge Discovery and Data Mining; 1996. p. 226-31. Available from: www.aaai.org.

84. Campello RJGB, Moulavi D, Sander J. Density-Based Clustering Based on Hierarchical Density Estimates. In: J P, V S T, L C, H M, G X, editors. Advances in Knowledge Discovery and Data Mining. PAKDD 2013. Lecture Notes in Computer Science. vol. 7819. Springer, Berlin, Heidelberg; 2013. p. 160-72. Available from: https://link.springer.com/chapter/10.1007/978-3-642-37456-2_14http://link.springer.com/10.1007/978-3-642-37456-2_14.

85. Campello RJGB, Moulavi D, Zimek A, Sander J. Hierarchical Density Estimates for Data Clustering, Visualization, and Outlier Detection. ACM Transactions on Knowledge Discovery from Data. 2015 7;10(1):1-51. Available from: https://dl.acm.org/doi/abs/10.1145/2733381https://dl.acm.org/doi/10.1145/2733381.

86. Sander J, Ester M, Kriegel HP, Xu X. Density-based clustering in spatial databases: The algorithm GDBSCAN and its applications. Data Mining and Knowledge Discovery. 1998;2(2):169-94.

87. Deng H, Han J. Probabilistic Models for Clustering. In: Aggarwal CC, Reddy CK, editors. Data Clustering: Algorithms and Applications. 1st ed. Chapman and Hall/CRC; 2014. p. 61-86.
